# Supplementary material for: Parental socioeconomic composition of birth cohorts changed during the COVID-19 pandemic
Source: Nat Commun. 2025 Dec 13;16:11477. doi: 10.1038/s41467-025-66264-z (PMC12749473; doi:10.1038/s41467-025-66264-z)
Supplement: Supplementary file 1 — Supplementary Information [file 41467_2025_66264_MOESM1_ESM.pdf]

SUPPLEMENTARY INFORMATION FOR:

Parental socioeconomic composition of birth cohorts changed during the COVID-19 pandemic

Authors: Moritz Oberndorfer[1,2,3], Juha Luukkonen[1,2], Hanna Remes[1,2], Thomas Waldhör[4], Lizbeth Burgos-Ochoa[5,6], Márta K. Radó[7], Jasper V. Been[6,8], Olof Östergren[9,10], Peter Fallesen[11,12], Alicia Montgomerie[13,14], Rhiannon Megan Pilkington[13,14], John Lynch[13,14,15], Enny S. Paixao[16,17], Ila R. Falcão[16], Pekka T. Martikainen[1,2,18,19]

[1] Helsinki Institute for Demography and Population Health, Faculty of Social Sciences, University of Helsinki, Helsinki, Finland

[2] Max Planck - University of Helsinki Center for Social Inequalities in Population Health, University of Helsinki, Helsinki, Finland

[3] MRC/CSO Social and Public Health Sciences Unit, University of Glasgow, Glasgow, United Kingdom

[4] Department of Epidemiology, Center for Public Health, Medical University of Vienna, Vienna, Austria

[5] Tilburg School of Social and Behavioural Sciences, Tilburg University, Tilburg, Netherlands

[6] Department of Obstetrics and Gynaecology, Erasmus MC Sophia Children's Hospital, University Medical Centre Rotterdam, Rotterdam, Netherlands

[7] Department of Medical Epidemiology and Biostatistics, Karolinska Institutet, Stockholm, Sweden

[8] Division of Neonatology, Department of Neonatal and Paediatric Intensive Care, Erasmus MC Sophia Children's Hospital, University Medical Centre Rotterdam, Rotterdam, Netherlands

[9] Department of Public Health Sciences, Stockholm University, Stockholm, Sweden

[10] Aging Research Center, Karolinska Insitutet, Solna, Sweden

[11] The Rockwool Foundation, Copenhagen, Denmark

[12] Swedish Institute for Social Research, Stockholm University, Stockholm, Sweden

[13] School of Public Health, The University of Adelaide, Australia

[14] Robinson Research Institute, The University of Adelaide, Australia

[15] Population Health Sciences, University of Bristol, United Kingdom

[16] Center for Data and Knowledge Integration for Health (CIDACS), Instituto Gonçalo Moniz, Fiocruz Bahia, Fundação Oswaldo Cruz, Salvador, Brazil

[17] Faculty of Epidemiology and Population Health, London School of Hygiene and Tropical Medicine, London, United Kingdom

[18] Max Planck Institute for Demographic Research, Rostock, Germany

[19] Department of Public Health Sciences, Stockholm University, Stockholm, Sweden

Contents

Supplementary methods 9

Supplementary discussion 10

Austria 18

- Data 18
- Results 18

Brazil 25

- Data 25
- Results 26

Colombia 31

- Data 31
- Results 32

Denmark 39

- Data 39
- Results 39

Ecuador 46

- Data 46
- Results 47

England 51

- Data 54
- Results 54

Finland 57

- Data 57
- Results 58

Mexico 63

- Data 63
- Results 64

Netherlands 71

- Data 71
- Results 72

Scotland 76

- Data 76
- Results 77

South Australia 80

- Data 80
- Results 81

Spain 85
Data 85
Results 86
Sweden 93
Data 93
Results 94
United States 99
Data 99
Results 100
Wales 107
Data 107
Results 107
References 110

Supplementary Table 1: Point estimates of differences between the observed and counterfactual socioeconomic composition of the December 2020 – December 2021 birth cohort. Counterfactual numbers of live births were estimated by interrupted time series Poisson regression models (see methods section). 95% confidence intervals for estimates are visually shown in Figures 4, and S2. Data for Spain only includes births among women aged older than 25 due to availability of maternal education (see methods and suppl. material)

Country	Characteristic	Observed Number	Counterfactual Number	Observed - Counterfactual Number	% more/less births observed than expected	Observed Proportion of Births in %	Counterfactual Proportion of Births in %	Observed - Counterfactual Proportion in %
**Austria**	**Maternal Education**							
Austria	Compulsory School	15481	14991	490	3.3	16.7	17.0	-0.3
Austria	Apprenticeship	20347	19332	1015	5.2	21.9	21.9	0.0
Austria	Technical; Vocational School	11336	11004	332	3.0	12.2	12.5	-0.3
Austria	Academic Upper Secondary	6304	5713	591	10.3	6.8	6.5	0.3
Austria	Post-Secondary; Tertiary	37847	35318	2529	7.2	40.7	40.0	0.8
Austria	Unknown	1633	2012	-379	-18.8	1.8	2.3	-0.5
Austria	Total	92948	88371	4577	5.2	100.0	100.0	0.0
**Brazil**	**Area Deprivation**							
Brazil	Q1 - most deprived	295939	281980	13959	5.0	10.6	10.3	0.4
Brazil	Q2	323640	312788	10852	3.5	11.6	11.4	0.2
Brazil	Q3	334608	327174	7434	2.3	12.0	11.9	0.1
Brazil	Q4	557568	549166	8402	1.5	20.0	20.0	0.0
Brazil	Q5 - least deprived	1275824	1278324	-2500	-0.2	45.8	46.5	-0.7
Brazil	Unknown	801	813	-12	-1.5	0.0	0.0	0.0
Brazil	Total	2788380	2750247	38133	1.4	100.0	100.0	0.0
**Colombia**	**Maternal Education**							
Colombia	Primary/Lower Secondary	239433	226444	12989	5.7	35.8	33.3	2.6
Colombia	Upper Secondary	246595	259256	-12661	-4.9	36.9	38.1	-1.2
Colombia	Post-Secondary; Tertiary	159921	167149	-7228	-4.3	23.9	24.5	-0.6
Colombia	Unknown	22415	28176	-5761	-20.4	3.4	4.1	-0.8

Colombia	Total	668364	681025	-12661	-1.9	100.0	100.0	0.0
**Denmark**	**Household Income**							
Denmark	Lowest 20%	9096	8135	961	11.8	13.9	13.4	0.5
Denmark	Q2	11303	10601	702	6.6	17.2	17.4	-0.2
Denmark	Q3	12937	12267	670	5.5	19.7	20.2	-0.4
Denmark	Q4	14813	13847	966	7.0	22.6	22.8	-0.2
Denmark	Highest 20%	15867	15011	856	5.7	24.2	24.7	-0.5
Denmark	Missing income information	1518	991	527	53.2	2.3	1.6	0.7
Denmark	Total	65534	60851	4683	7.7	100.0	100.0	0.0
**Ecuador**	**Area Deprivation**							
Ecuador	Q1 - most deprived	21861	20113	1748	8.7	8.4	7.5	0.9
Ecuador	Q2	27866	26015	1851	7.1	10.7	9.7	1.0
Ecuador	Q3	33220	32359	861	2.7	12.7	12.0	0.7
Ecuador	Q4	32902	32728	174	0.5	12.6	12.2	0.4
Ecuador	Q5 - least deprived	145294	157756	-12462	-7.9	55.6	58.7	-3.0
Ecuador	Total	261143	268972	-7829	-2.9	0.0	0.0	0.0
**England**	**Area Deprivation**							
England	Q1 - most deprived	157752	158360	-608	-0.4	24.6	25.6	-1.0
England	Q2	140452	138217	2235	1.6	21.9	22.3	-0.4
England	Q3	125637	120142	5495	4.6	19.6	19.4	0.2
England	Q4	114553	107398	7155	6.7	17.9	17.4	0.5
England	Q5 - least deprived	102394	94354	8040	8.5	16.0	15.3	0.7
England	Total	640788	618472	22316	3.6	100.0	100.0	0.0
**Finland**	**Household Income**							
Finland	Lowest 20%	6809	6189	620	10.0	14.2	14.4	-0.2
Finland	Q2	8106	7514	592	7.9	16.9	17.5	-0.6
Finland	Q3	9083	8122	961	11.8	18.9	18.9	0.1
Finland	Q4	10720	9820	900	9.2	22.3	22.8	-0.5
Finland	Highest 20%	12642	10969	1673	15.3	26.3	25.5	0.9
Finland	Missing income information	624	432	192	44.4	1.3	1.0	0.3

Finland	Total	47984	43047	4937	11.5	100.0	100.0	0.0
**Mexico**	**Maternal Education**							
Mexico	Elementary	248081	250220	-2139	-0.9	15.1	14.8	0.3
Mexico	Lower Secondary	585510	596123	-10613	-1.8	35.7	35.3	0.3
Mexico	Upper Secondary	715098	739122	-24024	-3.3	43.5	43.8	-0.3
Mexico	Unknown	93635	102284	-8649	-8.5	5.7	6.1	-0.4
Mexico	Total	1642324	1687750	-45426	-2.7	100.0	100.0	0.0
**Netherlands**	**Household Income**							
Netherlands	Poorest 20%	23674	22919	755	3.3	12.3	12.5	-0.2
Netherlands	Q2	21178	20426	752	3.7	11.0	11.2	-0.2
Netherlands	Q3	34168	32033	2135	6.7	17.8	17.5	0.2
Netherlands	Q4	49488	47197	2291	4.9	25.7	25.8	-0.1
Netherlands	Richest 20%	59846	55745	4101	7.4	31.1	30.5	0.6
Netherlands	Missing income information	3955	4348	-393	-9.0	2.1	2.4	-0.3
Netherlands	Total	192309	182669	9640	5.3	100.0	100.0	0.0
**Scotland**	**Area Deprivation**							
Scotland	Q1 - most deprived	11455	11605	-150	-1.3	23.2	24.3	-1.1
Scotland	Q2	10119	10153	-34	-0.3	20.5	21.3	-0.7
Scotland	Q3	8965	8698	267	3.1	18.2	18.2	0.0
Scotland	Q4	10124	9344	780	8.4	20.5	19.6	1.0
Scotland	Q5 - least deprived	8566	7828	738	9.4	17.4	16.4	1.0
Scotland	Unknown	94	116	-22	-18.8	0.2	0.2	-0.1
Scotland	Total	49323	47745	1578	3.3	100.0	100.0	0.0
**South Australia**	**Area Deprivation**							
South Australia	Q1 - most deprived	6056	5682	374	6.6	29.1	29.5	-0.4
South Australia	Q2	3667	3453	214	6.2	17.6	17.9	-0.3
South Australia	Q3	5121	4688	433	9.2	24.6	24.3	0.2
South Australia	Q4	4077	3617	460	12.7	19.6	18.8	0.8
South Australia	Q5 - least deprived	1924	1834	90	4.9	9.2	9.5	-0.3
South Australia	Total	20845	19275	1570	8.1	100.0	100.0	0.0

**Spain**	**Maternal Education**							
Spain	Primary/Lower Secondary	80993	82900	-1907	-2.3	25.2	25.3	0.0
Spain	Upper Secondary	55956	56812	-856	-1.5	17.4	17.3	0.1
Spain	Post-Secondary; Tertiary	176475	171982	4493	2.6	55.0	52.4	2.5
Spain	Unknown	7507	16213	-8706	-53.7	2.3	4.9	-2.6
Spain	Total	320931	327907	-6976	-2.1	100.0	100.0	0.0
**Sweden**	**Household Income**							
Sweden	Lowest 20%	21197	20668	529	2.6	18.1	18.3	-0.2
Sweden	Q2	22594	21621	973	4.5	19.3	19.1	0.2
Sweden	Q3	25082	23996	1086	4.5	21.4	21.2	0.2
Sweden	Q4	23068	22580	488	2.2	19.7	20.0	-0.3
Sweden	Highest 20%	21053	20294	759	3.7	18.0	17.9	0.0
Sweden	Missing income information	4143	3914	229	5.9	3.5	3.5	0.1
Sweden	Total	117137	113073	4064	3.6	100.0	100.0	0.0
**United States**	**Maternal Education**							
United States	No Highschool	433594	444395	-10801	-2.4	11.0	11.4	-0.4
United States	Highschool	1752085	1745654	6431	0.4	44.3	44.7	-0.4
United States	Post-Secondary; Tertiary	1707681	1647083	60598	3.7	43.2	42.2	1.0
United States	Unknown	60810	64144	-3334	-5.2	1.5	1.6	-0.1
United States	Total	3954170	3901276	52894	1.4	100.0	100.0	0.0
**Wales**	**Area Deprivation**							
Wales	Q1 - most deprived	7512	7631	-119	-1.6	24.3	25.3	-1.0
Wales	Q2	6437	6184	253	4.1	20.8	20.5	0.3
Wales	Q3	6064	5892	172	2.9	19.6	19.5	0.1
Wales	Q4	5735	5359	376	7.0	18.5	17.7	0.8
Wales	Q5 - least deprived	5221	5141	80	1.6	16.9	17.0	-0.2
Wales	Total	30969	30208	761	2.5	100.0	100.0	0.0

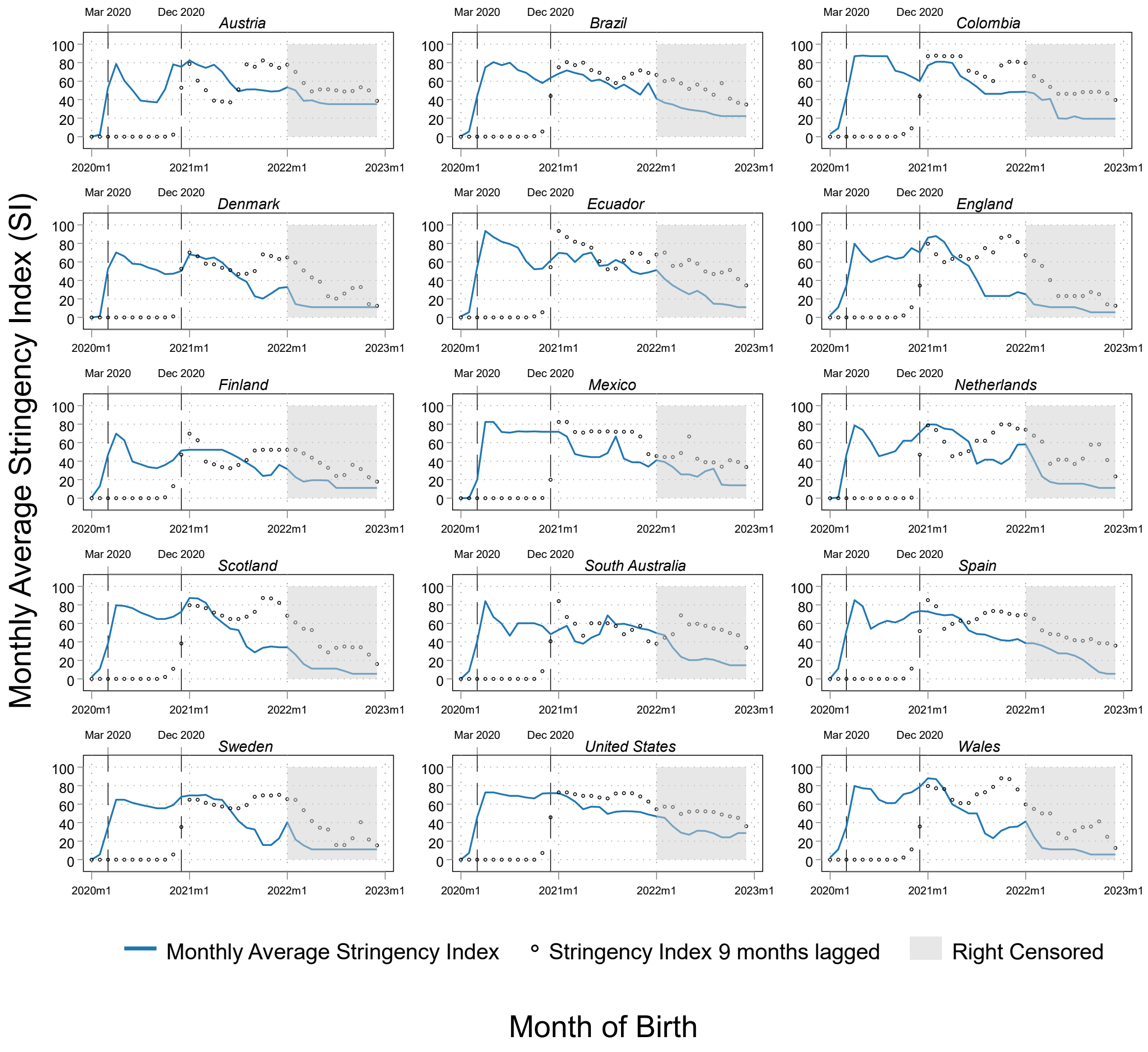

Supplementary Figure 1: Monthly average Stringency Index (SI) of government's national lockdown policies for all included countries indicated by solid lines. Circles show the 9 months lagged SI value to indicate the SI level around conception (9 months prior to the month of birth). The grey shaded area highlights the SI levels during the months of birth and conception for live births not included in our data.

Supplementary methods

For all country profiles, we used the analytical strategy presented in the main manuscript. Thus, all results can be interpreted in the same way.

Every country profile contains information on the data source used as well as figures showing the difference between the observed and counterfactual number of live births for each subgroup of the main socioeconomic variable. In addition, we show the same results for available alternative socioeconomic parental characteristics (e.g., taking the highest education of parents instead of just maternal education), maternal age, and parity. As the availability and quality of these parental characteristics varies by country, the country profiles below include a different range of variables.

At the end of each country profile, we present a table that summarises differences between the observed and counterfactual cohort of babies born between December 2020 and December 2021 for each analysed parental characteristic.

Before we present the country profiles, we show comparative figures of i) the percentage point differences in the proportion of live births and ii) the relative difference in the number of live births between the observed and counterfactual birth cohort December 2020 – December 2021 for each subgroup within available parental characteristics (an alternative indicator for parental socioeconomic circumstances, maternal age, and parity). As the percentage point differences in the cohort composition for the primary indicator of parental socioeconomic circumstances is presented in the main manuscript, we only present the relative differences for this indicator below. For the relative differences, the group with missing information is omitted due to visual distortion but the results are presented in the tables of the country profiles. Note that our chosen model specification sometimes fails to capture the erratic behaviour in the weekly/monthly number of live births with missing information on parental characteristics. This yields a less plausible estimate of the counterfactual number of live births with missing information on parental characteristics. A careful interpretation is warranted.

A short description of these results is given in the main manuscript.

Analyses were carried out in Stata v18[1] and all code for the statistical analysis is openly available at https://github.com/MoritzOberndorfer/COVID-19-pandemic-changed-the-socioeconomic-composition-of-parents and at https://doi.org/10.5281/zenodo.15585751.

Supplementary discussion

Comparative Results for Socioeconomic, Age, and Parity Composition

Complementary to differences between observed and counterfactual proportions in the main manuscript, we show the relative differences between the observed and counterfactual number of births by socioeconomic group and country in Supplementary Figure 2. The same differences in the proportions of live births can be the result of different underlying changes in the number of live births. Supplementary Figure 2 shows that, for example, compositional differences in Brazil were driven by a socioeconomic gradient in the surplus in the number of live births whereby the least disadvantaged show no change and the more disadvantaged, the higher was the relative increase in the number of births (Supplementary Figure 2).

In Supplementary Figure 3, we show the differences in observed and counterfactual proportions of live births along alternative or more detailed indicators for parental socioeconomic circumstances available for 12 out of 15 countries. Here, compositional change is less evident for Brazil and Finland where we used maternal education instead of area deprivation and quintile of household income respectively (Supplementary Figure 3). Conversely, compositional differences were larger for Austria and Spain where we used the highest parental education (and paternal education if maternal education was missing) instead of maternal education (Supplementary Figure 3). Complementary we again show relative differences between observed and counterfactual number of live births by alternative socioeconomic indicators in Supplementary Figure 4.

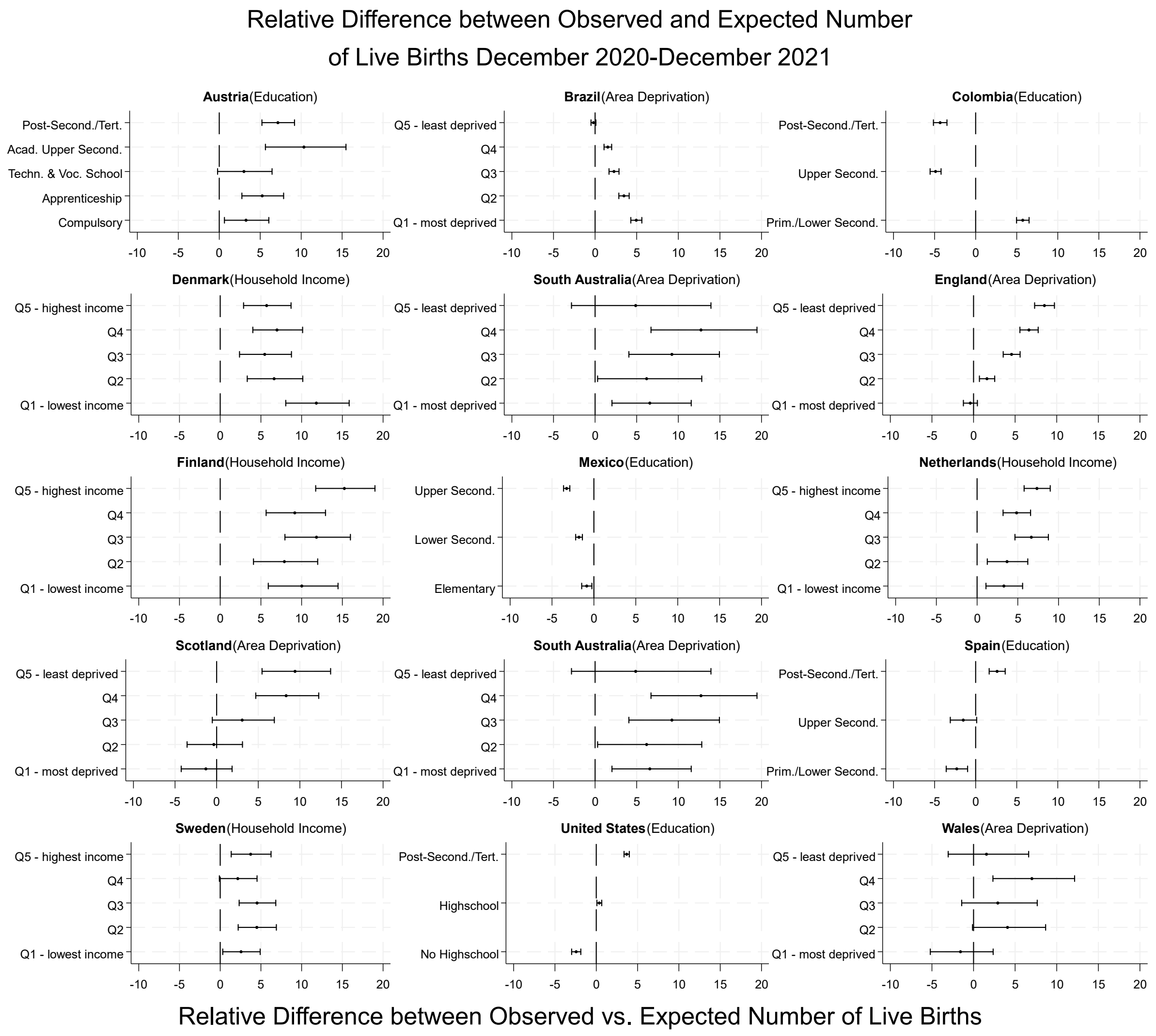

Supplementary Figure 2: Relative differences in the number of live births between the observed and counterfactual birth cohort December 2020 – December 2021 by primary indicator of parental socioeconomic circumstances. Point estimates and 95% confidence intervals are estimated by taking the difference between the observed number of live births and the point estimates of the counterfactual number of live births (or upper and lower bounds of their 95% confidence intervals) divided by the respective counterfactual number of live births. For this visualisation, the relative difference is multiplied by 100.

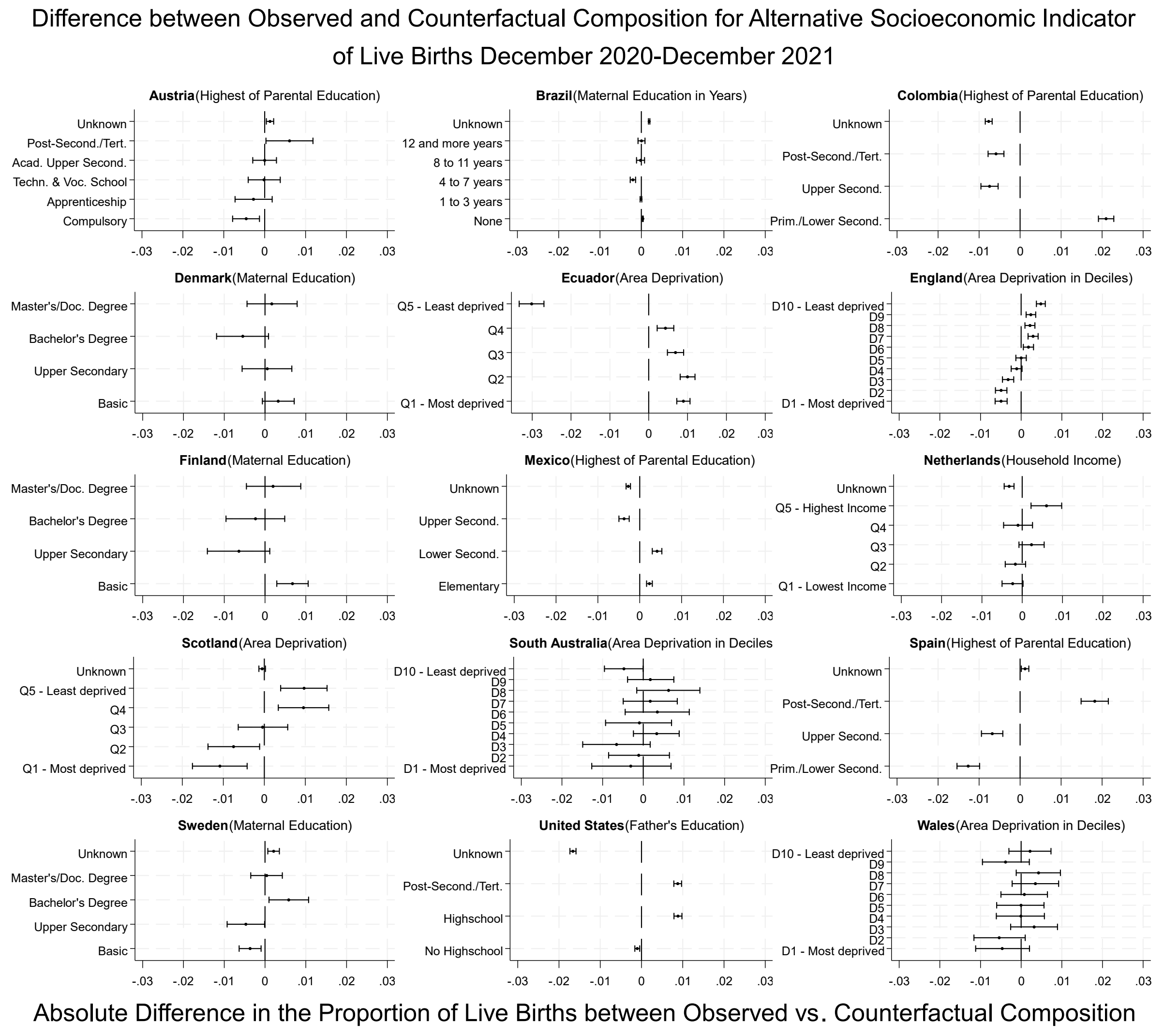

Supplementary Figure 3: Percentage point differences in the socioeconomic composition of live births between the observed and counterfactual birth cohort December 2020 – December 2021. For all countries, except Ecuador, Scotland, and the Netherlands (for which we used the primary indicators), we used alternative or more detailed indicators of parental socioeconomic circumstances. The group with missing information ("unknown") is omitted when there were no or a negligible number of live births with missing information. Point estimates and 95% confidence intervals are estimated as described in the methods section of the main manuscript.

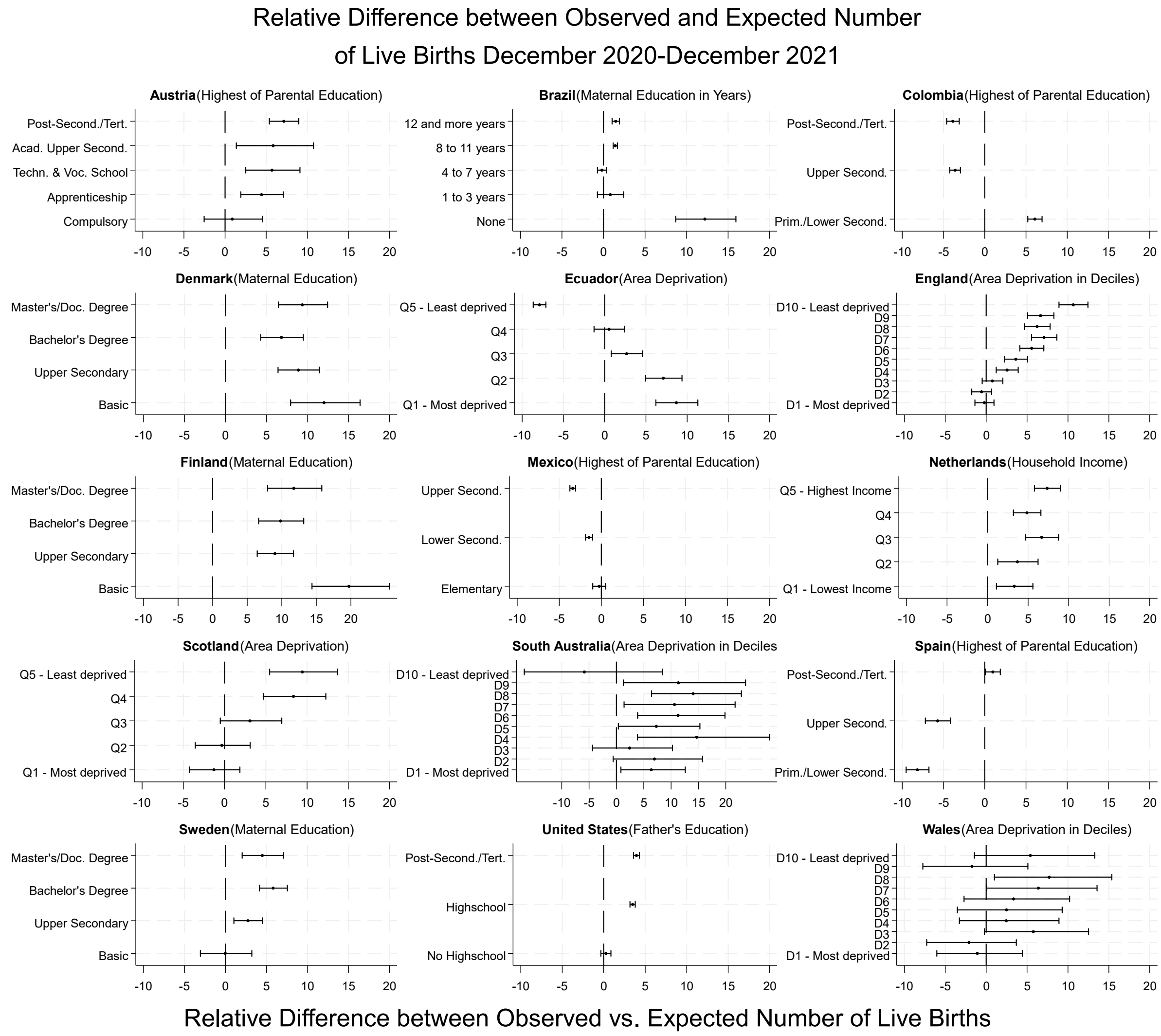

Supplementary Figure 4: Relative differences in the number of live births between the observed and counterfactual birth cohort December 2020 – December 2021 by alternative indicator of parental socioeconomic circumstances. For all countries, except Ecuador, Scotland, and the Netherlands (for which we used the primary indicators), we used alternative or more detailed indicators of parental socioeconomic circumstances. Point estimates and 95% confidence intervals are estimated by taking the difference between the observed number of live births and the point estimates of the counterfactual number of live births (or upper and lower bounds of their 95% confidence intervals) divided by the respective counterfactual number of live births. For this visualisation, the relative difference is multiplied by 100.

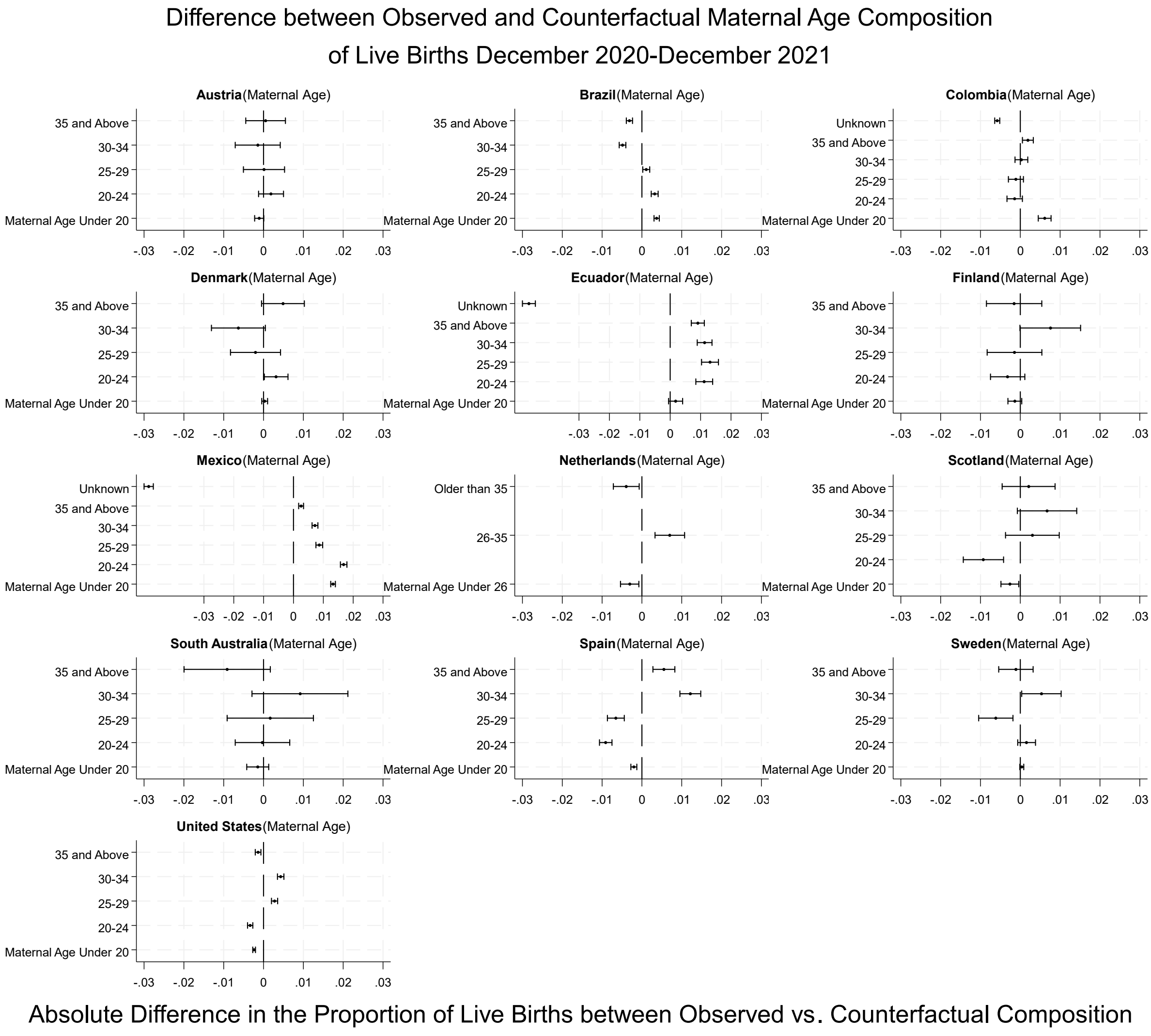

Supplementary Figure 5: Percentage point differences in the maternal age composition of live births between the observed and counterfactual birth cohort December 2020 – December 2021. Point estimates and 95% confidence intervals are estimated as described in the methods section of the main manuscript. The group with missing information ("unknown") is omitted when there were no or a negligible number of live births with missing information.

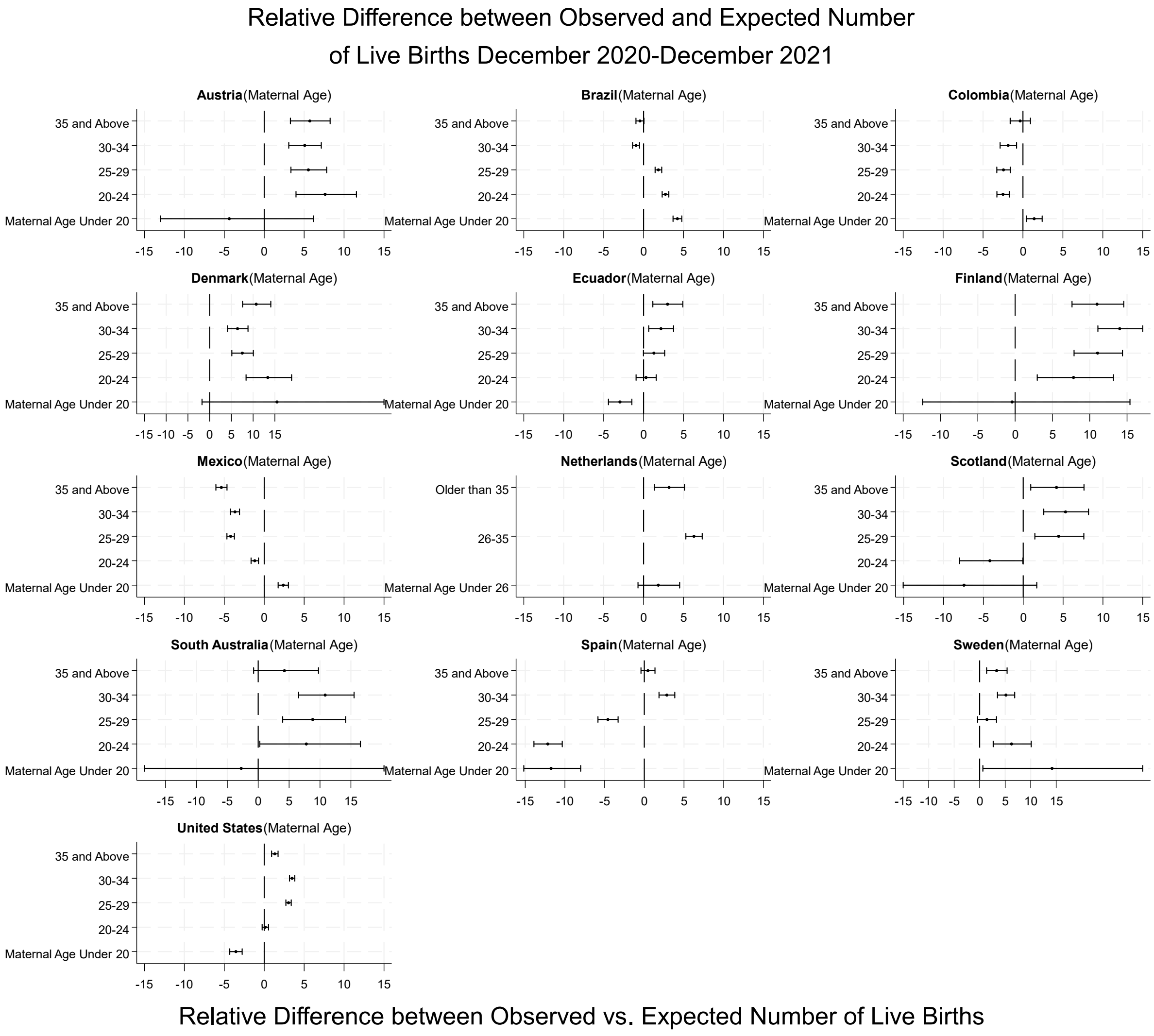

Supplementary Figure 6: Relative differences in the number of live births between the observed and counterfactual birth cohort December 2020 – December 2021 by maternal age. Point estimates and 95% confidence intervals are estimated by taking the difference between the observed number of live births and the point estimates of the counterfactual number of live births (or upper and lower bounds of their 95% confidence intervals) divided by the respective counterfactual number of live births. For this visualisation, the relative difference is multiplied by 100.

Difference between Observed and Counterfactual Parity Composition of Live Births December 2020-December 2021

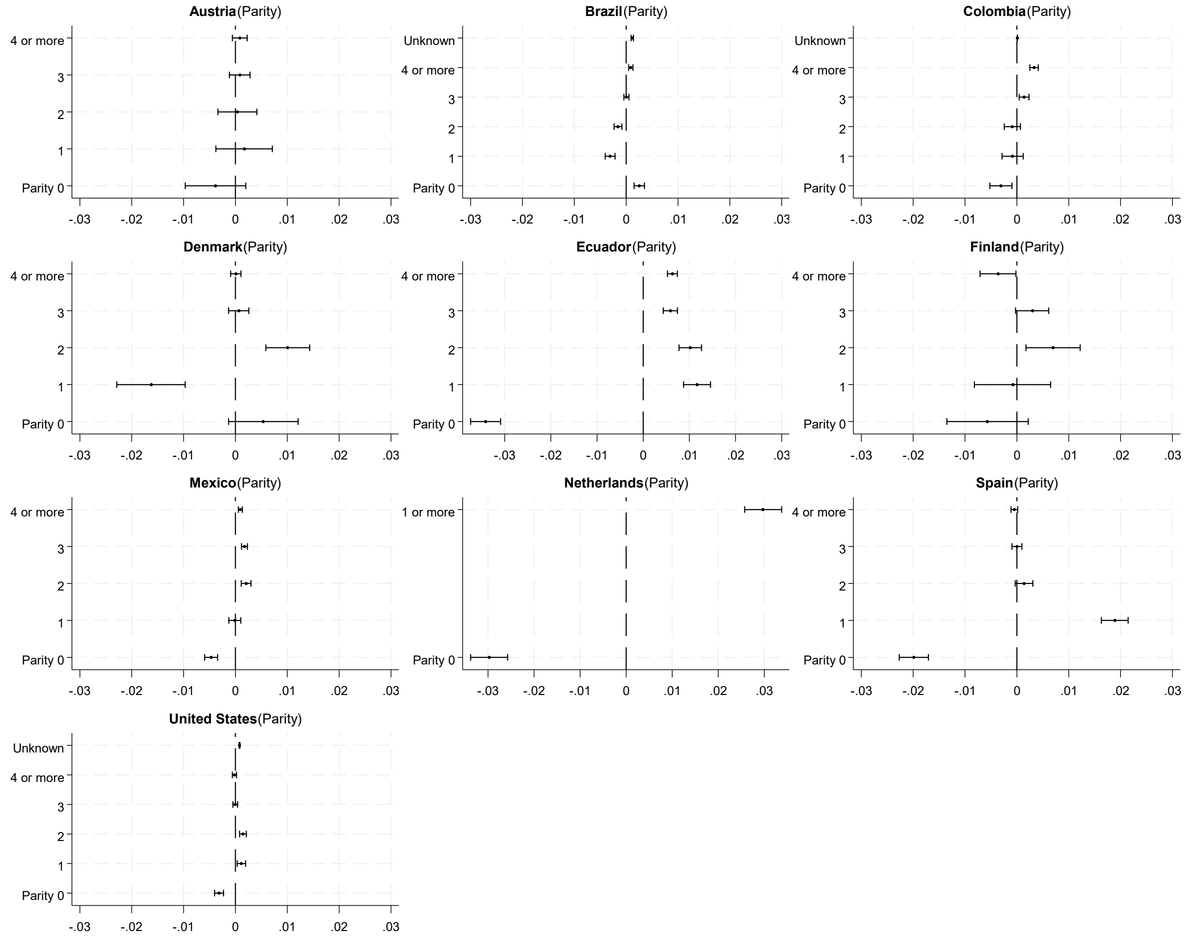

Absolute Difference in the Proportion of Live Births between Observed vs. Counterfactual Composition

Supplementary Figure 7: Percentage point differences in the parity composition of live births between the observed and counterfactual birth cohort December 2020 – December 2021. Point estimates and 95% confidence intervals are estimated as described in the methods section of the main manuscript. The group with missing information ("unknown") is omitted when there were no or a negligible number of live births with missing information.

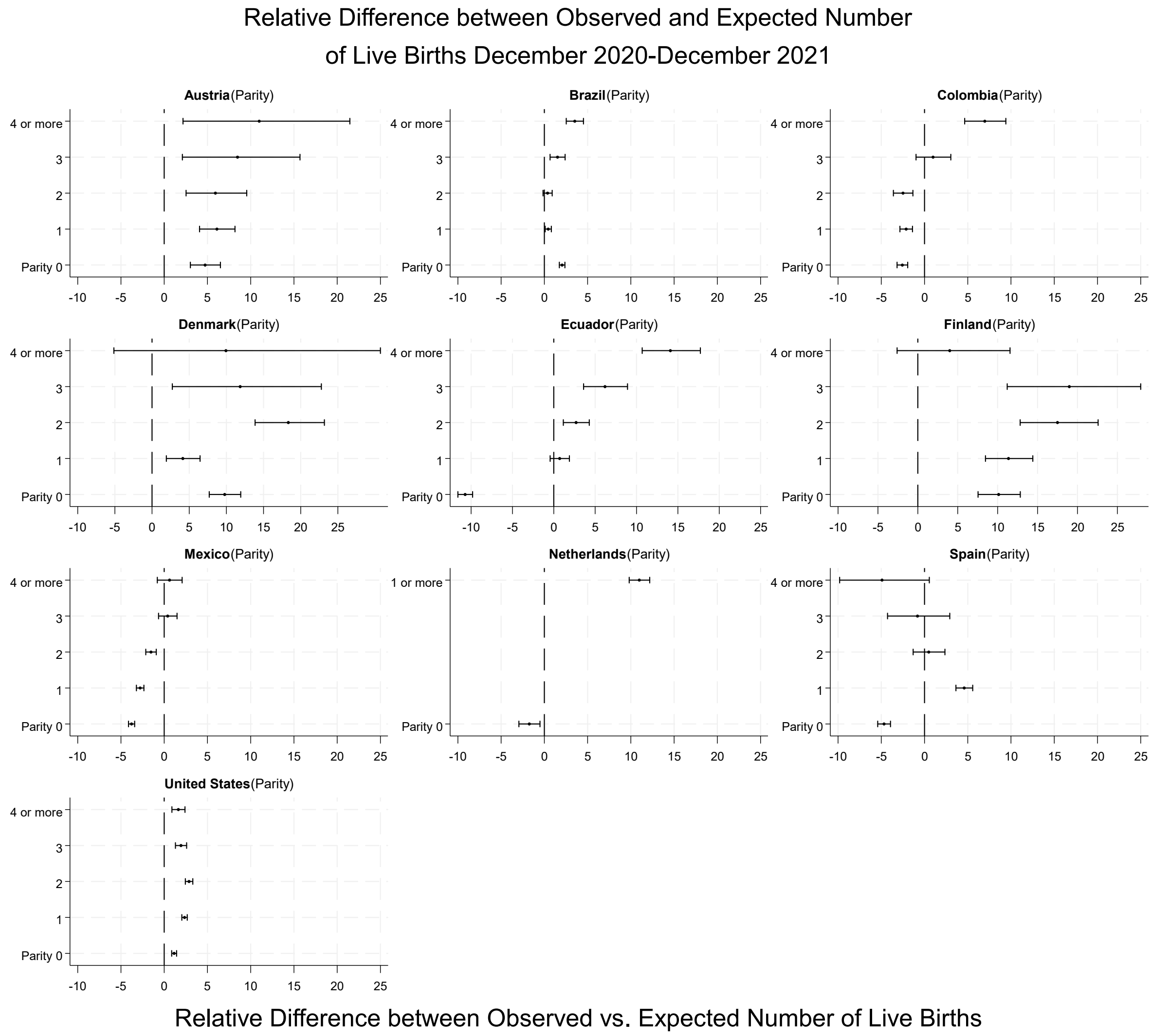

Supplementary Figure 8: Relative differences in the number of live births between the observed and counterfactual birth cohort December 2020 – December 2021 by parity. Point estimates and 95% confidence intervals are estimated by taking the difference between the observed number of live births and the point estimates of the counterfactual number of live births (or upper and lower bounds of their 95% confidence intervals) divided by the respective counterfactual number of live births. For this visualisation, the relative difference is multiplied by 100.

Austria

Data

For Austria, we used the birth register (2015-2021; n=601,886 live births) maintained by Statistics Austria. We used individual-level data and then aggregated to monthly time series by parental characteristics of interest. Available characteristics were maternal and paternal education at birth, maternal age, and parity.

Austria's educational system separates children at a young age. Therefore, we were reluctant to from an "upper secondary school" category and instead show the educational levels in more detail (Figure S8, S9). The missing values for maternal education increase in 2021 because they are obtained from population registers. The availability of these data is time lagged compared to the birth registers. The change in missing data through time is thus not induced by the COVID-19 pandemic but an artefact of the data collection.

Individual-level data used to create aggregated monthly time series was obtained by Thomas Waldhör through purchase of the data from Statistics Austria.

Results

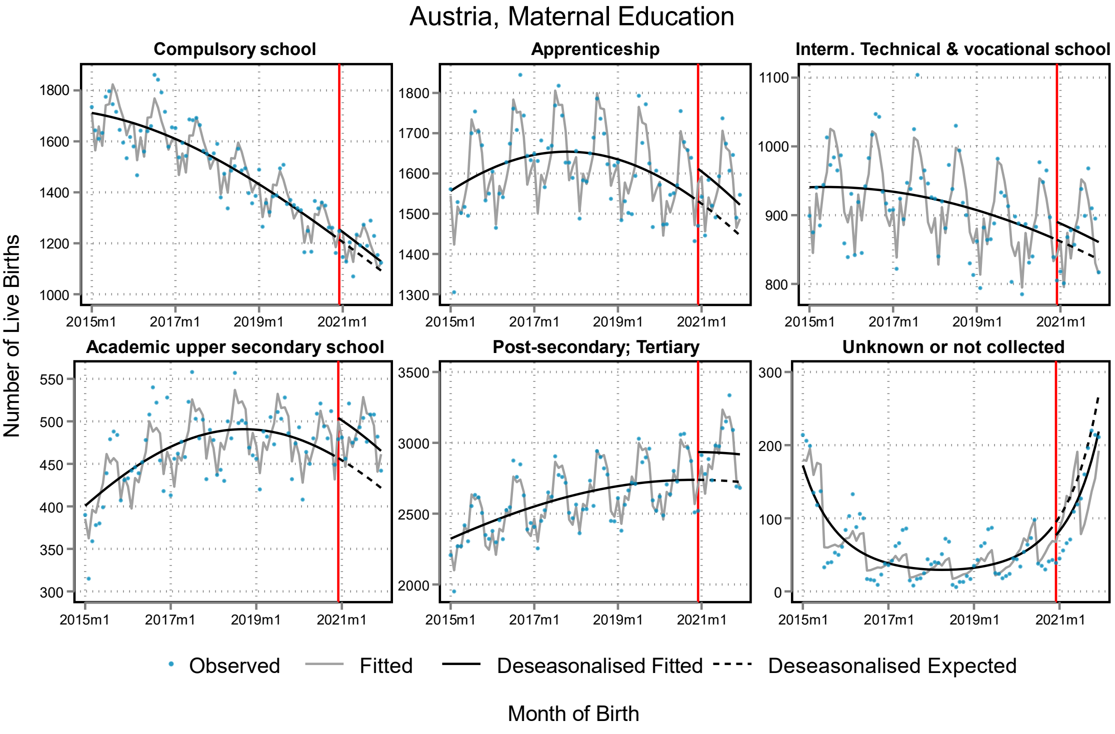

*Supplementary Figure 9: Observed and expected monthly number of live births in **Austria** by primary socioeconomic indicator maternal education. Expected numbers are estimated by subgroup-specific Poisson regression modes on the full time series including an indicator variable for the exposed period (starting December 2020 to December 2021) to estimate the average effect of the COVID-19 pandemic over the entire period; a linear and a quadratic term for month of live birth to capture potential non-linearities in the secular time trends; month of the year fixed effects to account for seasonality.*

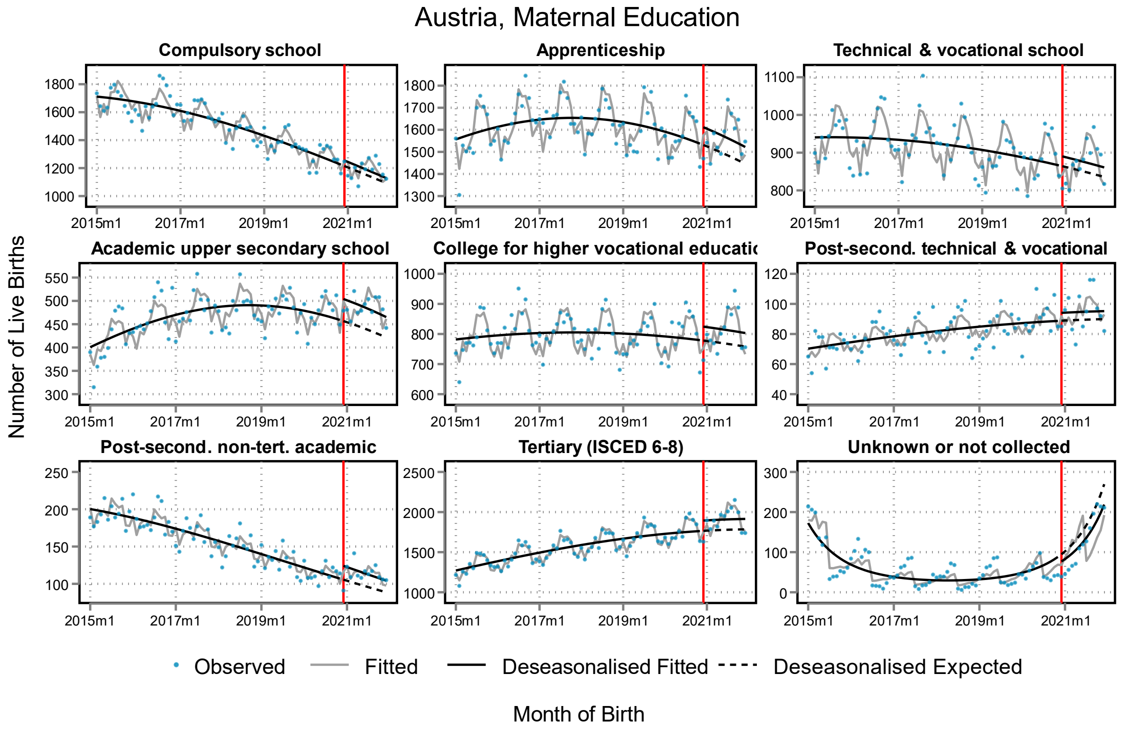

Supplementary Figure 10: Observed and expected monthly number of live births in ***Austria*** *by maternal education in more detail. Expected numbers are estimated by subgroup-specific Poisson regression modes on the full time series including an indicator variable for the exposed period (starting December 2020 to December 2021) to estimate the average effect of the COVID-19 pandemic over the entire period; a linear and a quadratic term for month of live birth to capture potential non-linearities in the secular time trends; month of the year fixed effects to account for seasonality.*

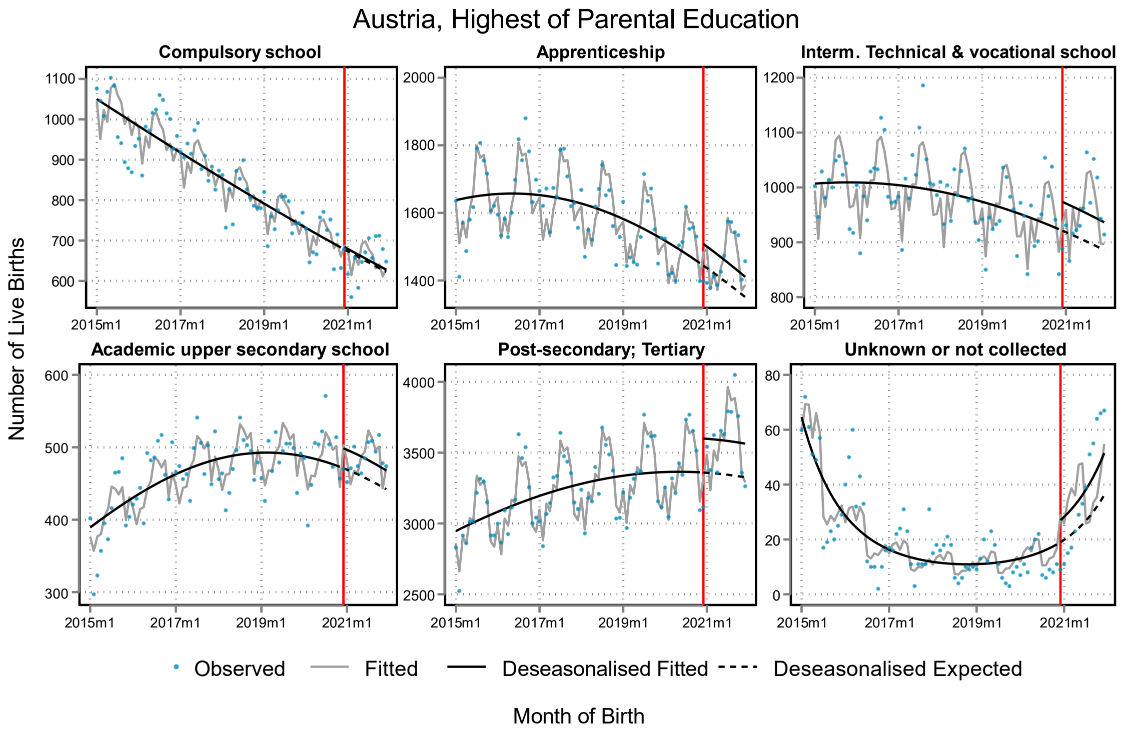

Supplementary Figure 11: Observed and expected monthly number of live births in ***Austria*** *by highest education of both parents. If maternal education was missing and paternal was non-missing, we used paternal education. Expected numbers are estimated by subgroup-specific Poisson regression modes on the full time series including an indicator variable for the exposed period (starting December 2020 to December 2021) to estimate the average effect of the COVID-19 pandemic over the entire period; a linear and a quadratic term for month of live birth to capture potential non-linearities in the secular time trends; month of the year fixed effects to account for seasonality.*

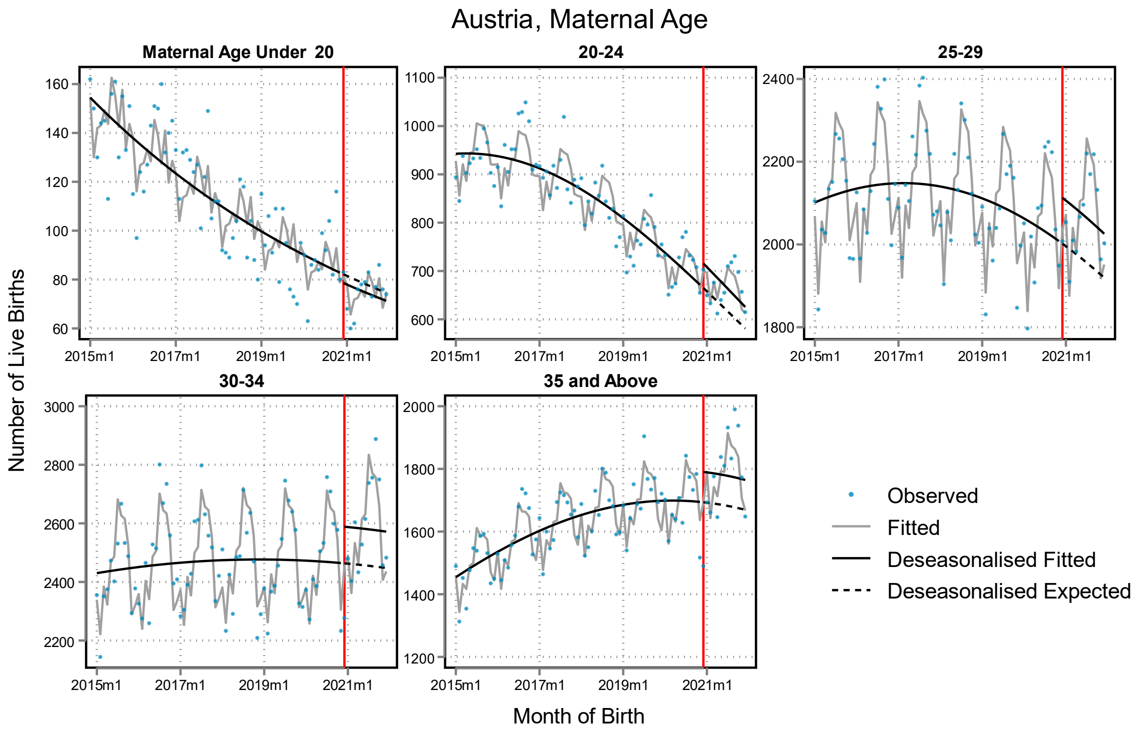

Supplementary Figure 12: Observed and expected monthly number of live births in Austria by maternal age. Expected numbers are estimated by subgroup-specific Poisson regression modes on the full time series including an indicator variable for the exposed period (starting December 2020 to December 2021) to estimate the average effect of the COVID-19 pandemic over the entire period; a linear and a quadratic term for month of live birth to capture potential non-linearities in the secular time trends; month of the year fixed effects to account for seasonality.

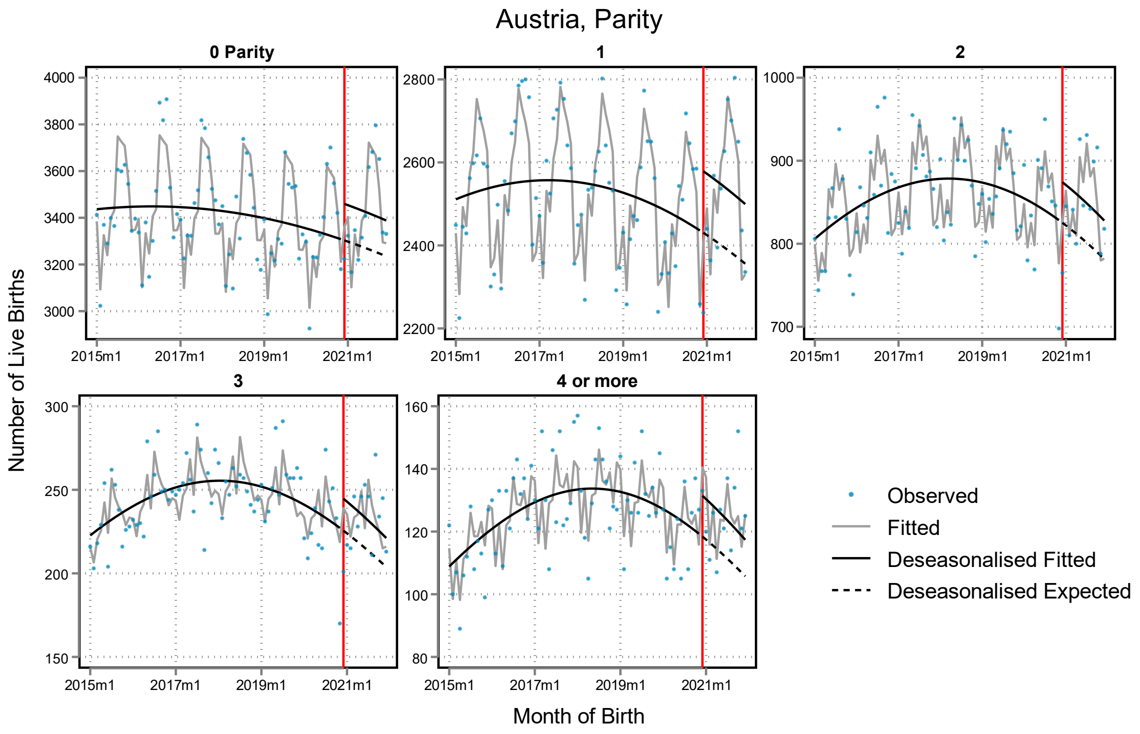

Supplementary Figure 13: Observed and expected monthly number of live births in ***Austria*** *by parity. Expected numbers are estimated by subgroup-specific Poisson regression modes on the full time series including an indicator variable for the exposed period (starting December 2020 to December 2021) to estimate the average effect of the COVID-19 pandemic over the entire period; a linear and a quadratic term for month of live birth to capture potential non-linearities in the secular time trends; month of the year fixed effects to account for seasonality.*

Supplementary Table 2: Relative and Percentage Point Differences in the Composition of the December 2020 – December 2021 Birth Cohort in Austria. "Observed" is abbreviated by "OBS" and "Counterfactual" is abbreviated by "CF". Statistical methods for the estimations are described in the main manuscript.

Characteristic	Observed (OBS) Births	Counterfactual (CF) Births	95%CI: CF Births	OBS - CF Births	95%CI: OBS - CF Births	% more/less than CF	95%CI: % more/less than CF	OBS proportion in %	CF proportion in %	OBS - CF proportion	95%CI: OBS - CF proportion
**Maternal education detailed**											
Compulsory	15481	14991	(14598; 15384)	490	(28; 953)	3.3	(0.6; 6.0)	16.7	17.0	-0.3	(-0.7; 0.1)
Apprenticeship	20347	19332	(18865; 19800)	1015	(470; 1560)	5.2	(2.8; 7.9)	21.9	21.9	0.0	(-0.5; 0.5)
Technical & Vocational School	11336	11004	(10649; 11359)	332	(-80; 744)	3.0	(-0.2; 6.4)	12.2	12.5	-0.3	(-0.6; 0.1)
Academic Upper Secondary School	6304	5713	(5459; 5967)	591	(293; 889)	10.3	(5.6; 15.5)	6.8	6.5	0.3	(0.0; 0.6)
College for Higher Vocational Education	10512	9922	(9583; 10261)	590	(196; 984)	5.9	(2.4; 9.7)	11.3	11.2	0.1	(-0.3; 0.4)
Post-secondary technical & vocational	1229	1161	(1040; 1281)	68	(-71; 207)	5.9	(-4.1; 18.2)	1.3	1.3	0.0	(-0.1; 0.1)
Post-secondary non-tertiary academic	1475	1254	(1148; 1361)	221	(90; 351)	17.6	(8.4; 28.5)	1.6	1.4	0.2	(0.0; 0.3)
Tertiary (ISCED 6-8)	24631	22963	(22424; 23502)	1668	(1047; 2288)	7.3	(4.8; 9.8)	26.5	26.0	0.5	(-0.0; 1.0)
Unknown	1633	2012	(1765; 2259)	-379	(-638; -120)	-18.8	(-27.7; -7.5)	1.8	2.3	-0.5	(-0.8; -0.3)
**total**	92948	88353		4595		0.1		100.0	100.0	0.0	
**Highest Parental Education**											
Compulsory	8527	8453	(8157; 8750)	74	(-274; 421)	0.9	(-2.5; 4.5)	9.2	9.6	-0.5	(-0.8; -0.1)
Apprenticeship	18953	18149	(17701; 18596)	804	(282; 1327)	4.4	(1.9; 7.1)	20.4	20.7	-0.3	(-0.7; 0.2)
Interim. Technical & Vocational School	12378	11710	(11345; 12075)	668	(243; 1093)	5.7	(2.5; 9.1)	13.3	13.3	0.0	(-0.4; 0.4)
Academic Upper Secondary School	6296	5948	(5685; 6211)	348	(42; 654)	5.8	(1.4; 10.7)	6.8	6.8	0.0	(-0.3; 0.3)
Post-secondary; Tertiary	46315	43227	(42506; 43949)	3088	(2252; 3923)	7.1	(5.4; 9.0)	49.8	49.2	0.6	(0.0; 1.2)
Unknown	479	337	(259; 415)	142	(53; 231)	42.2	(15.5; 85.0)	0.5	0.4	0.1	(0.0; 0.2)

**total**	92948	87824		5124		0.1		100.0	100.0	0.0	
**Maternal age**											
Below 20	975	1020	(918; 1121)	-45	(-163; 74)	-4.4	(-13.0; 6.2)	1.0	1.2	-0.1	(-0.2; 0.0)
20-24	8698	8081	(7798; 8365)	617	(279; 954)	7.6	(4.0; 11.5)	9.4	9.2	0.2	(-0.1; 0.5)
25-29	26835	25428	(24889; 25967)	1407	(780; 2034)	5.5	(3.3; 7.8)	28.9	28.9	0.0	(-0.5; 0.5)
30-34	33412	31800	(31184; 32415)	1612	(900; 2324)	5.1	(3.1; 7.1)	35.9	36.1	-0.1	(-0.7; 0.4)
Above 34	23028	21785	(21273; 22297)	1243	(651; 1835)	5.7	(3.3; 8.3)	24.8	24.7	0.1	(-0.4; 0.6)
**total**	92948	88114		4834		0.1		100.0	100.0	0.0	
**Parity**											
0	44419	42411	(41708; 43115)	2008	(1192; 2824)	4.7	(3.0; 6.5)	47.8	48.2	-0.4	(-1.0; 0.2)
1	32848	30961	(30363; 31558)	1887	(1192; 2582)	6.1	(4.1; 8.2)	35.3	35.2	0.2	(-0.4; 0.7)
2	11027	10411	(10067; 10755)	616	(215; 1017)	5.9	(2.5; 9.5)	11.9	11.8	0.0	(-0.3; 0.4)
3	3026	2790	(2615; 2964)	236	(32; 441)	8.5	(2.1; 15.7)	3.3	3.2	0.1	(-0.1; 0.3)
4 or more	1628	1467	(1340; 1593)	161	(12; 310)	11.0	(2.2; 21.5)	1.8	1.7	0.1	(-0.1; 0.2)
**total**	92948	88039		4909		0.1		100.0	100.0	0.0	

Brazil

Data

For Brazil (2015-2021, n=20,000,327 live births), we used openly available individual-level data from SINASC (Sistema de Informações sobre Nascidos Vivos) (https://opendatasus.saude.gov.br/dataset/sistema-de-informacao-sobre-nascidos-vivos-sinasc). SINASC contains national records of live births, and it covers close to 100% of all live births in Brazil.[2] We used individual-level data and then aggregated to weekly time series by parental characteristics of interest. To measure relative material deprivation of mothers' residential area, we used the BrazDep small-area deprivation index.[3] This index is created from the 2010 Brazilian Population Census data and combines i) the percent of household with per capita income smaller than 50% of the minimum wage, ii) percent of people not literate (older than 7), and iii) average percent of people with inadequate access to sewage, water, garbage, collection and no toilet and bath/shower. The BrazDep is developed for the smallest geographical level (310,120 census sectors; average population size ~615 residents in 2010) and was validated against health outcomes (see report by Allik et al. 2020). As the smallest geographical level available in SINASC is municipality (5565 municipalities; average population size ~34,100 in 2010), we needed to aggregate the BrazDep small-area deprivation measure to the municipality level. We did this by creating an average of the values of census sectors within municipalities weighted by the share of the population of each census sectors. Note that the BrazDep small-area deprivation measure uses data from 2010. One the one hand, this means that the COVID-19 pandemic had no impact on the deprivation measure we use. On the other, this time lag in the deprivation measure will probably induce measurement error. As areas have been shown to change their relative material positions only slowly (if at all)[4], we do not believe that the number of areas which drastically changed in their material deprivation is big enough to cause serious misclassification in quintiles of deprivation. Another advantage of the area-level measure is that information on municipality of maternal residence is far more complete than information on maternal education.

As secondary measure of socioeconomic circumstances, we used maternal education available in the SINASC data. Additionally, we estimated compositional change for maternal age and parity.

Results

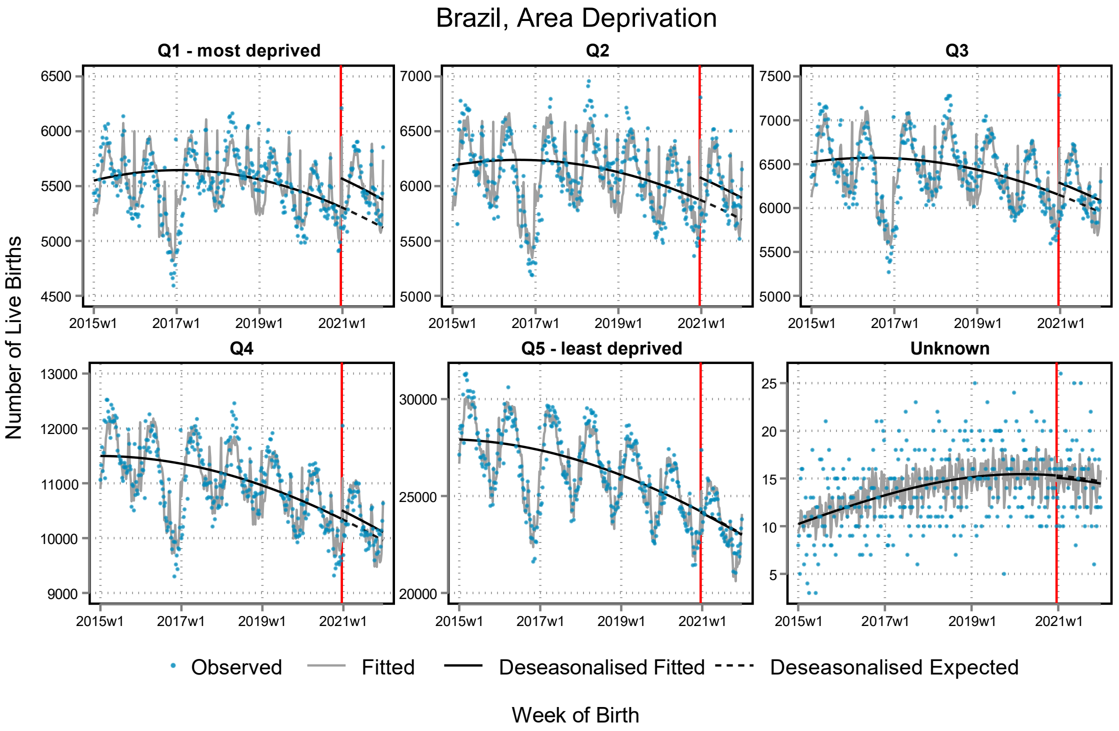

Supplementary Figure 14: Observed and expected weekly number of live births in ***Brazil*** *by primary measure of socioeconomic circumstances (quintile of area level deprivation). Expected numbers are estimated by subgroup-specific Poisson regression modes on the full time series including an indicator variable for the exposed period (starting second week of December 2020 to December 2021) to estimate the average effect of the COVID-19 pandemic over the entire period; a linear and a quadratic term for week of live birth to capture potential non-linearities in the secular time trends; week of the year fixed effects to account for seasonality; an indicator variable (August 2016-December 2016) to account for the 2015-2016 Zika virus epidemic.*

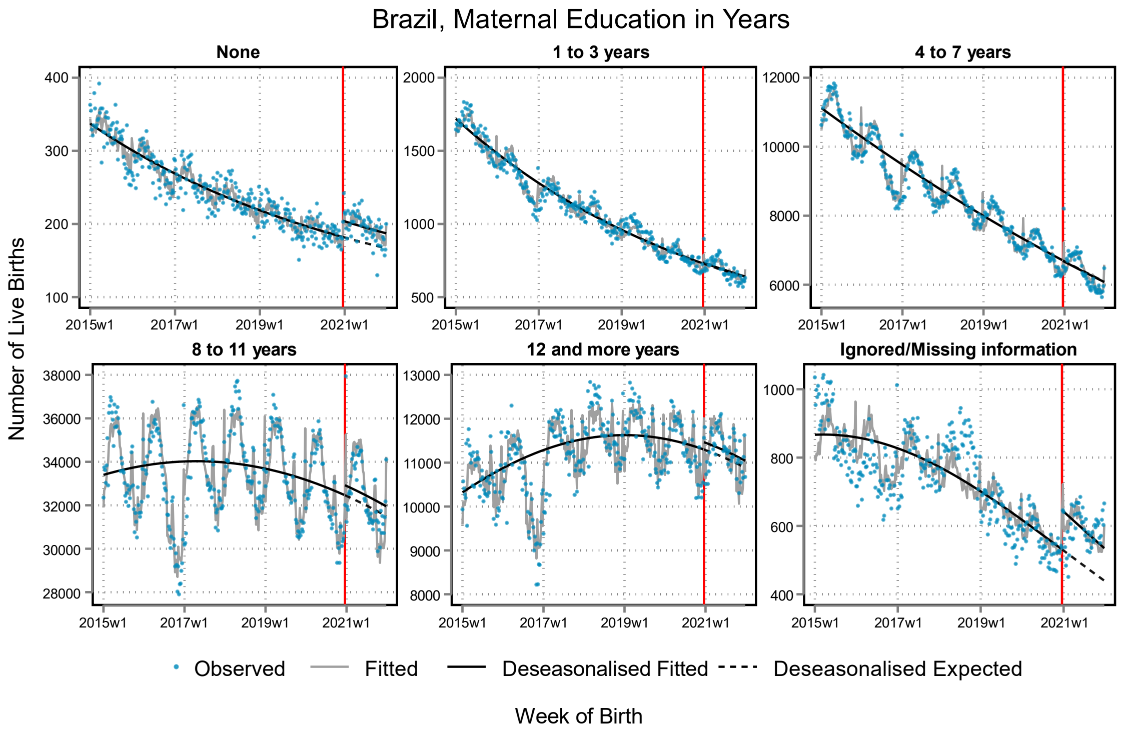

Supplementary Figure 15: Observed and expected weekly number of live births in ***Brazil*** *by secondary measure of socioeconomic circumstances (maternal education). Expected numbers are estimated by subgroup-specific Poisson regression modes on the full time series including an indicator variable for the exposed period (starting second week of December 2020 to December 2021) to estimate the average effect of the COVID-19 pandemic over the entire period; a linear and a quadratic term for week of live birth to capture potential non-linearities in the secular time trends; week of the year fixed effects to account for seasonality; an indicator variable (August 2016-December 2016) to account for the 2015-2016 Zika virus epidemic*

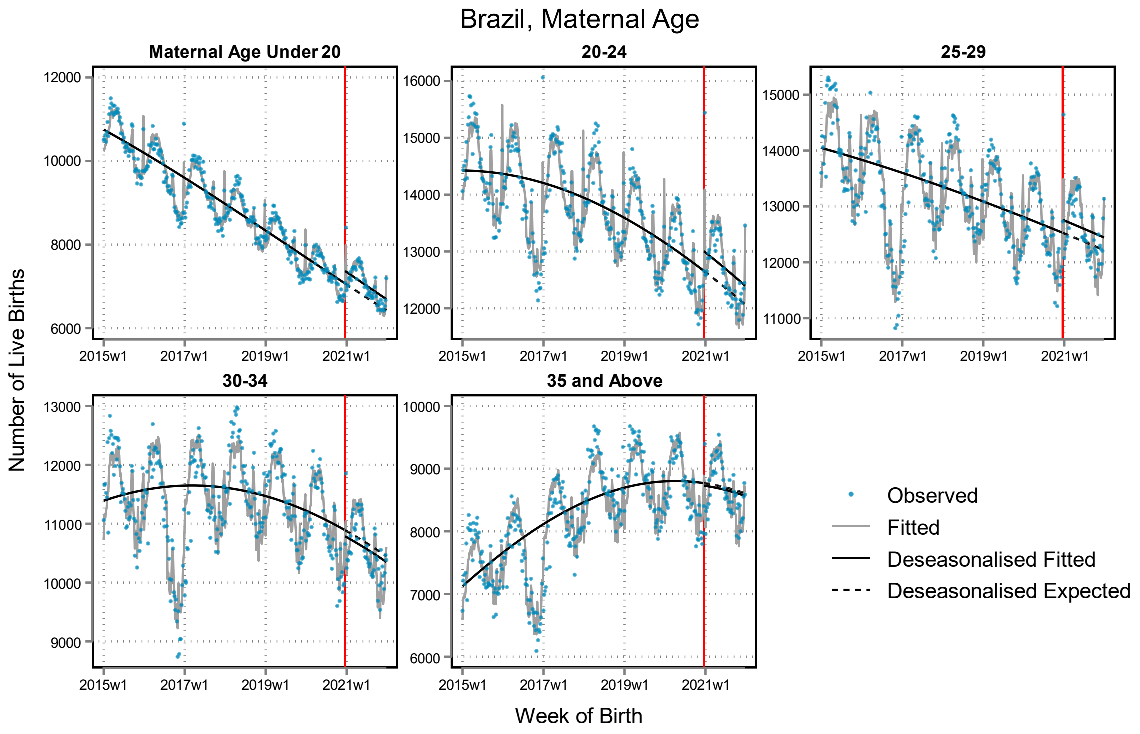

*Supplementary Figure 16: Observed and expected weekly number of live births in **Brazil** by maternal age. Expected numbers are estimated by subgroup-specific Poisson regression modes on the full time series including an indicator variable for the exposed period (starting second week of December 2020 to December 2021) to estimate the average effect of the COVID-19 pandemic over the entire period; a linear and a quadratic term for week of live birth to capture potential non-linearities in the secular time trends; week of the year fixed effects to account for seasonality; an indicator variable (August 2016-December 2016) to account for the 2015-2016 Zika virus epidemic.*

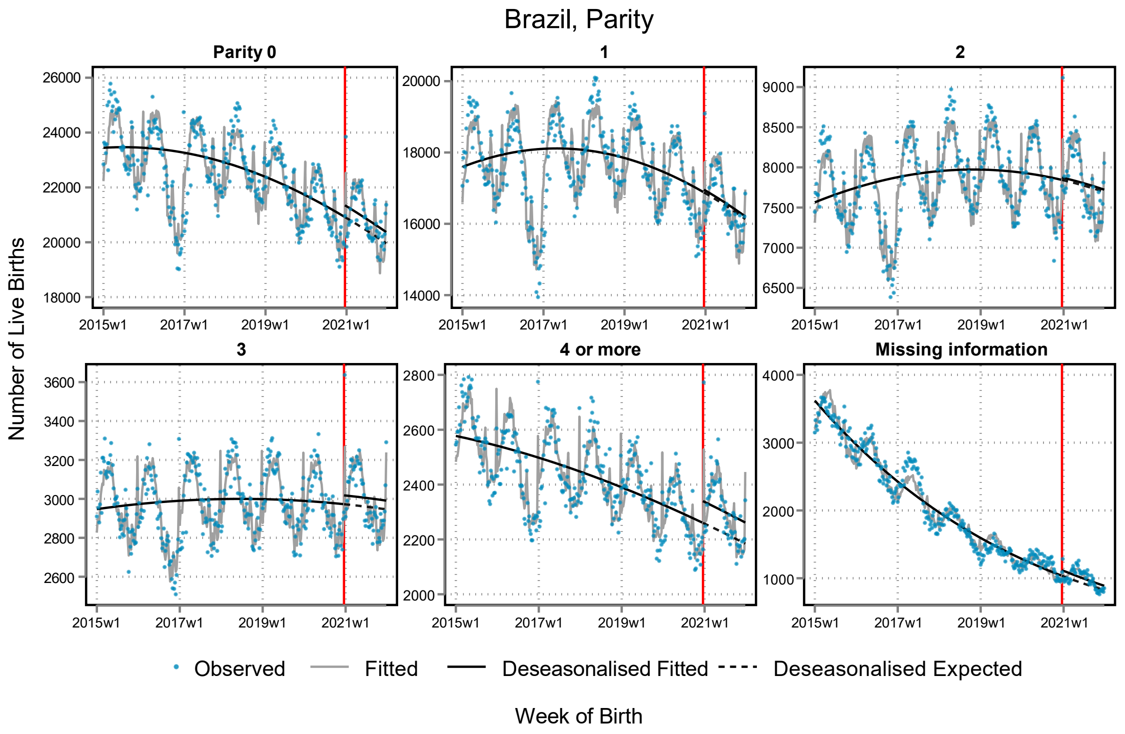

*Supplementary Figure 17: Observed and expected weekly number of live births in **Brazil** by parity. Expected numbers are estimated by subgroup-specific Poisson regression modes on the full time series including an indicator variable for the exposed period (starting second week of December 2020 to December 2021) to estimate the average effect of the COVID-19 pandemic over the entire period; a linear and a quadratic term for week of live birth to capture potential non-linearities in the secular time trends; week of the year fixed effects to account for seasonality; an indicator variable (August 2016-December 2016) to account for the 2015-2016 Zika virus epidemic.*

Supplementary Table 3: Relative and Percentage Point Differences in the Composition of the December 2020 – December 2021 Birth Cohort in Brazil. "Observed" is abbreviated by "OBS" and "Counterfactual" is abbreviated by "CF". Statistical methods for the estimations are described in the main manuscript.

Characteristic	Observed (OBS) Births	Counterfactual (CF) Births	95%CI: CF Births	OBS - CF Births	95%CI: OBS - CF Births	% more/less than CF	95%CI: % more/less than CF	OBS proportion	CF proportion	OBS - CF proportion	95%CI: OBS - CF proportion
**Maternal education in years**											
None	10559	9411	(9108; 9714)	1148	(784; 1512)	12.2	(8.7; 15.9)	0.4	0.3	0.0	(0.0; 0.0)
1-3 years	37136	36831	(36254; 37408)	305	(-385; 995)	0.8	(-0.7; 2.4)	1.3	1.3	0.0	(-0.0; 0.0)
4-7 years	345098	345759	(343905; 347613)	-661	(-2844; 1522)	-0.2	(-0.7; 0.3)	12.4	12.6	-0.2	(-0.3; -0.1)
8-11 years	1754767	1730192	(1725759; 1734624)	24575	(19438; 29712)	1.4	(1.2; 1.7)	62.9	63.0	0.0	(-0.1; 0.1)
12 years and more	608863	599998	(597376; 602620)	8865	(5829; 11901)	1.5	(1.0; 1.9)	21.8	21.8	0.0	(-0.1; 0.1)
Unknown	31957	26278	(25791; 26765)	5679	(5079; 6279)	21.6	(19.4; 23.9)	1.1	1.0	0.2	(0.2; 0.2)
**total**	2788380	2748468		39912		0.0		100.0	100.0	0.0	
**Maternal age**											
Below 20	380232	364793	(362879; 366708)	15439	(13175; 17703)	4.2	(3.7; 4.8)	13.6	13.3	0.4	(0.3; 0.4)
20-24	687112	668808	(666097; 671518)	18304	(15144; 21464)	2.7	(2.3; 3.2)	24.6	24.3	0.3	(0.2; 0.4)
25-29	681519	669110	(666364; 671855)	12409	(9222; 15596)	1.9	(1.4; 2.3)	24.4	24.3	0.1	(0.0; 0.2)
30-34	571615	577081	(574537; 579625)	-5466	(-8410; -2522)	-0.9	(-1.4; -0.5)	20.5	21.0	-0.5	(-0.6; -0.4)
Above 34	467840	469983	(467620; 472347)	-2143	(-4861; 574)	-0.5	(-1.0; 0.0)	16.8	17.1	-0.3	(-0.4; -0.2)
**total**	2788318	2749775		38543		0.0		100.0	100.0	0.0	
**Parity**											
0	1128507	1105785	(1102293; 1109277)	22722	(18656; 26788)	2.1	(1.7; 2.4)	40.5	40.2	0.3	(0.2; 0.4)
1	897185	893176	(890017; 896335)	4009	(345; 7673)	0.4	(0.1; 0.8)	32.2	32.5	-0.3	(-0.4; -0.2)
2	421817	420243	(418037; 422449)	1574	(-973; 4121)	0.4	(-0.1; 0.9)	15.1	15.3	-0.2	(-0.2; -0.1)
3	162545	160108	(158742; 161474)	2437	(859; 4015)	1.5	(0.7; 2.4)	5.8	5.8	0.0	(-0.0; 0.1)
4 or more	124373	120147	(118991; 121304)	4226	(2879; 5573)	3.5	(2.5; 4.5)	4.5	4.4	0.1	(0.0; 0.1)
Unknown	53953	49954	(49331; 50577)	3999	(3227; 4770)	8.0	(6.7; 9.4)	1.9	1.8	0.1	(0.1; 0.1)
**total**	2788380	2749413		38967		0.0		100.0	100.0	0.0	

Colombia

Data

For Colombia (2015-2021, n=4,503,315 live births), we used openly available individual-level vital statistics from DANE (Departamento Administrativo Nacional De Estatdistica) (https://microdatos.dane.gov.co/index.php/catalog/DEM-Microdatos#_r=1700214975152&collection=&country=&dtype=&from=2015&page=1&ps=&sid=&sk=&sort_by=title&sort_order=&to=2023&topic=&view=s&vk=). Close to 100% of births in Colombia are covered by these data as survey research found that the proportion of unregistered children under age 5 was 2% at highest in the regions with the lowest coverage (Caribbean, Eastern, Pacific).[5]

We aggregated the data to monthly time series of the number of live births by parental characteristics. The thirteen levels of parental education were translated to more comparable ISCED (International Standard Classification of Education) categories using OECD's profile of Colombia's educational system (https://gpseducation.oecd.org/CountryProfile?primaryCountry=COL&treshold=5&topic=EO). Having non, pre-school, primary school, and lower secondary school was grouped together as primary or lower secondary education (all qualifications up to ISCED 2). ISCED 3 level qualifications were grouped together as upper secondary and ISCED 4 to ISCED 8 were grouped together as post-secondary non-tertiary and tertiary education.

As secondary measure of socioeconomic circumstances, we used the highest education of mother or father and father's education if maternal education was not available. Additionally, we estimated compositional change for maternal education in more detail, maternal age, and parity.

Results

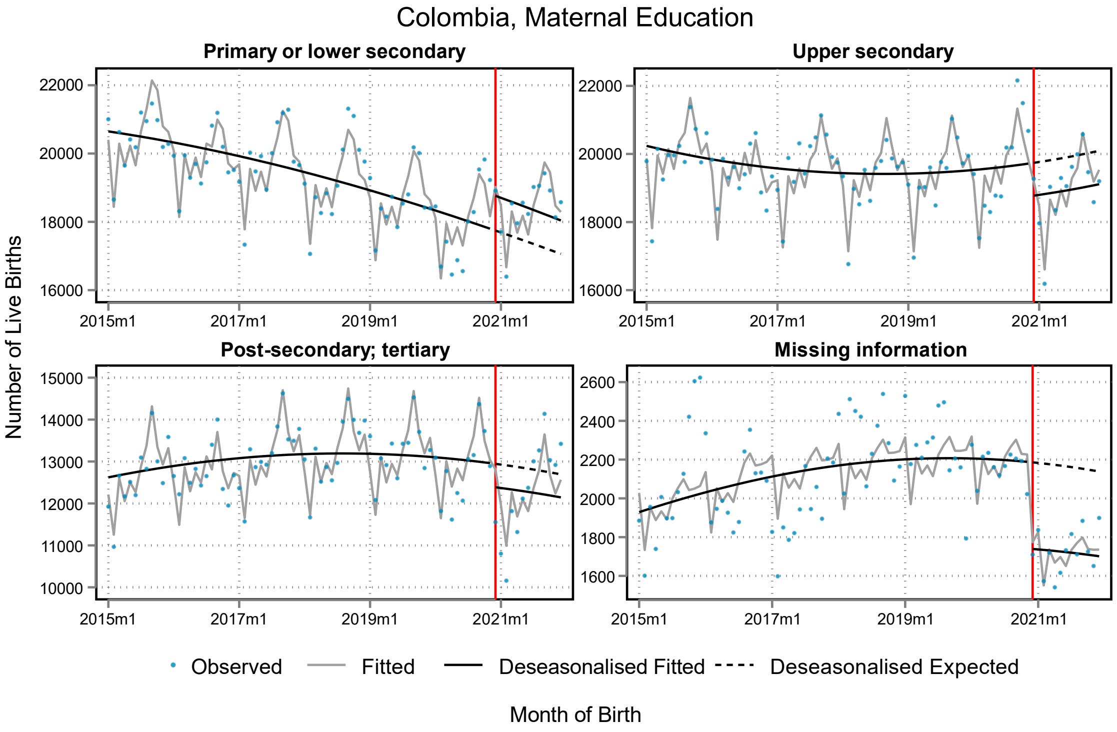

*Supplementary Figure 18: Observed and expected monthly number of live births in **Colombia** by primary indicator of socioeconomic circumstances (maternal education). Expected numbers are estimated by subgroup-specific Poisson regression modes on the full time series including an indicator variable for the exposed period (starting December 2020 to December 2021) to estimate the average effect of the COVID-19 pandemic over the entire period; a linear and a quadratic term for week of live birth to capture potential non-linearities in the secular time trends; month of the year fixed effects to account for seasonality; an indicator variable (August 2016-December 2016) to account for the 2015-2016 Zika virus epidemic.*

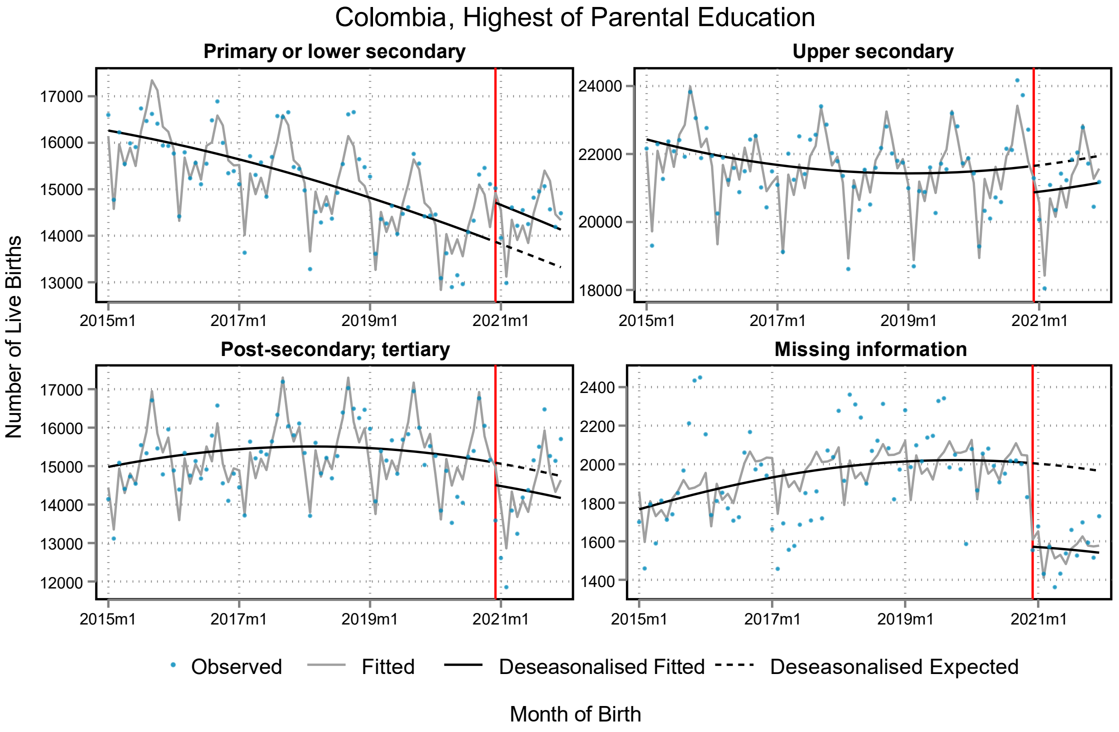

*Supplementary Figure 19: Observed and expected monthly number of live births in **Colombia** by highest of parental education. We used paternal education if maternal education was missing. Expected numbers are estimated by subgroup-specific Poisson regression modes on the full time series including an indicator variable for the exposed period (starting December 2020 to December 2021) to estimate the average effect of the COVID-19 pandemic over the entire period; a linear and a quadratic term for week of live birth to capture potential non-linearities in the secular time trends; month of the year fixed effects to account for seasonality; an indicator variable (August 2016-December 2016) to account for the 2015-2016 Zika virus epidemic.*

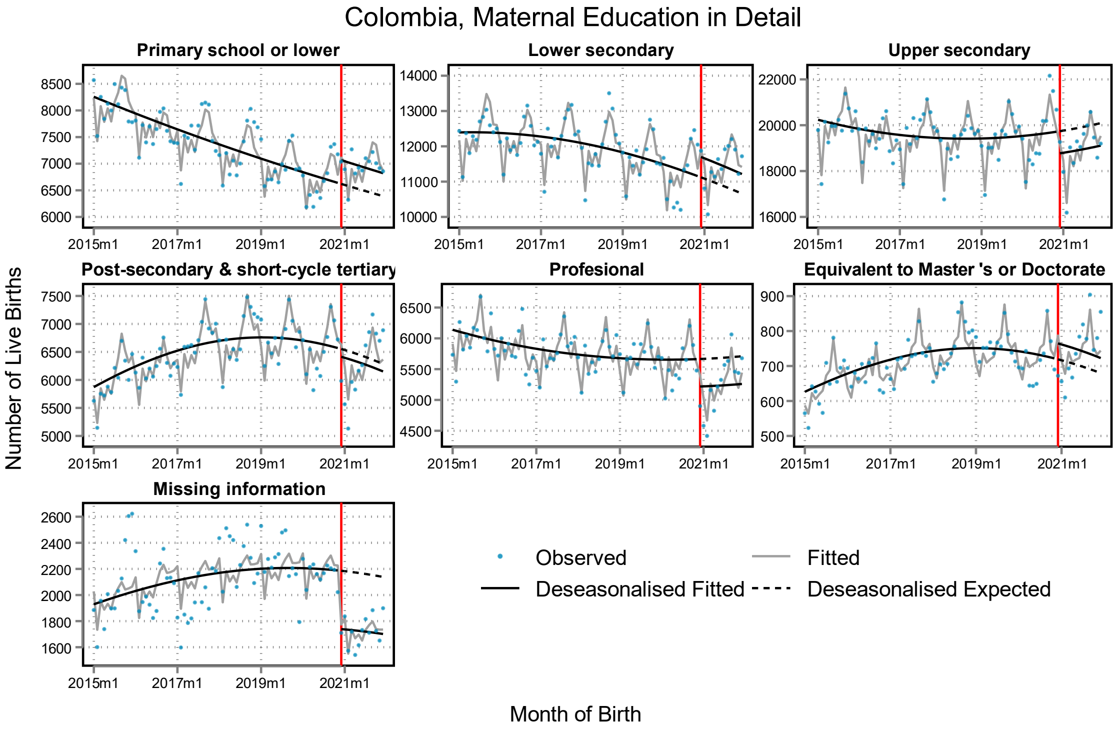

*Supplementary Figure 20: Observed and expected monthly number of live births in **Colombia** by maternal education in detail. Expected numbers are estimated by subgroup-specific Poisson regression modes on the full time series including an indicator variable for the exposed period (starting December 2020 to December 2021) to estimate the average effect of the COVID-19 pandemic over the entire period; a linear and a quadratic term for week of live birth to capture potential non-linearities in the secular time trends; month of the year fixed effects to account for seasonality; an indicator variable (August 2016-December 2016) to account for the 2015-2016 Zika virus epidemic.*

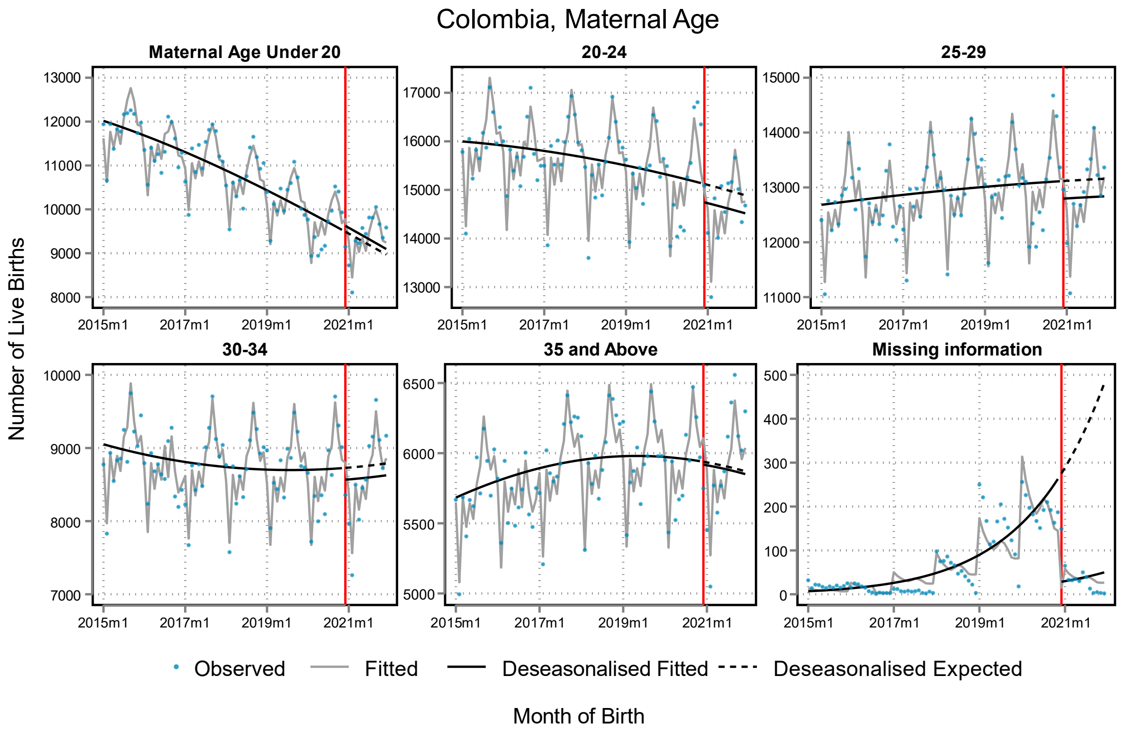

*Supplementary Figure 21: Observed and expected monthly number of live births in **Colombia** by maternal age. Expected numbers are estimated by subgroup-specific Poisson regression modes on the full time series including an indicator variable for the exposed period (starting December 2020 to December 2021) to estimate the average effect of the COVID-19 pandemic over the entire period; a linear and a quadratic term for week of live birth to capture potential non-linearities in the secular time trends; month of the year fixed effects to account for seasonality; an indicator variable (August 2016-December 2016) to account for the 2015-2016 Zika virus epidemic.*

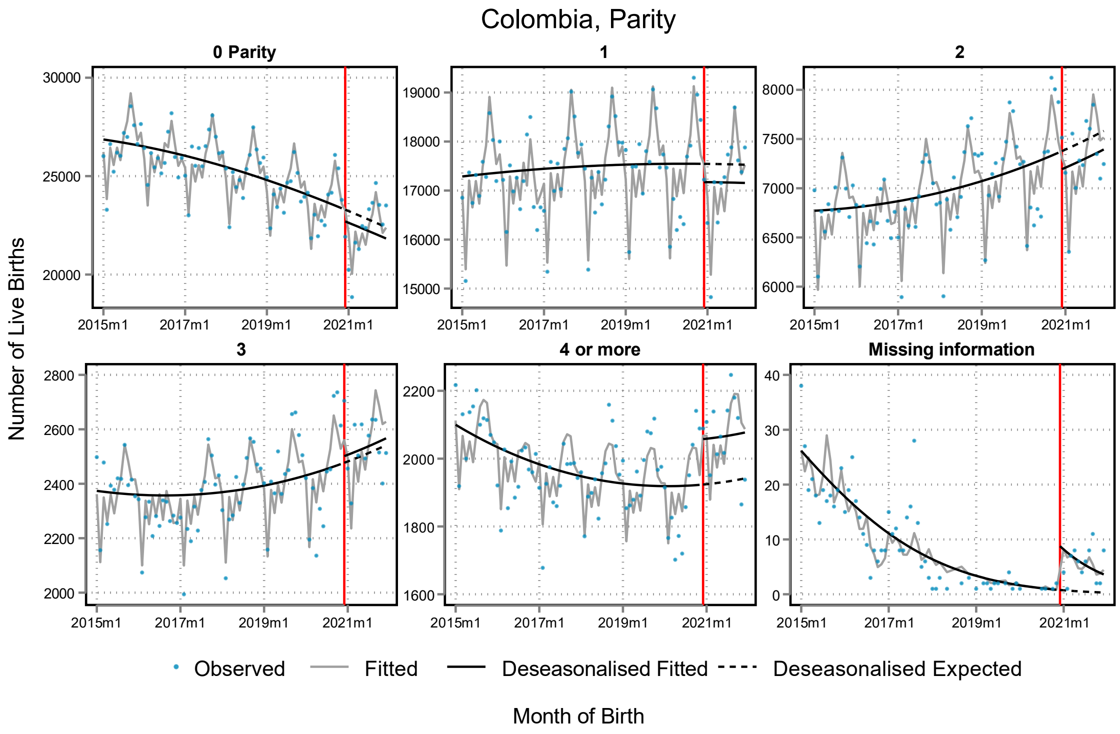

*Supplementary Figure 22: Observed and expected monthly number of live births in **Colombia** by parity. Expected numbers are estimated by subgroup-specific Poisson regression modes on the full time series including an indicator variable for the exposed period (starting December 2020 to December 2021) to estimate the average effect of the COVID-19 pandemic over the entire period; a linear and a quadratic term for week of live birth to capture potential non-linearities in the secular time trends; month of the year fixed effects to account for seasonality; an indicator variable (August 2016-December 2016) to account for the 2015-2016 Zika virus epidemic.*

Supplementary Table 4: Relative and Percentage Point Differences in the Composition of the December 2020 – December 2021 Birth Cohort in Colombia. "Observed" is abbreviated by "OBS" and "Counterfactual" is abbreviated by "CF". Statistical methods for the estimations are described in the main manuscript.

Characteristic	Observed (OBS) Births	Counterfactual (CF) Births	95%CI: CF Births	OBS - CF Births	95%CI: OBS - CF Births	% more/less than CF	95%CI: % more/less than CF	OBS proportion	CF proportion	OBS - CF proportion	95%CI: OBS - CF proportion
**Highest Parental Education**											
Primary or Lower Secondary	187661	176912	(175472; 178352)	10749	(9077; 12421)	6.1	(5.2; 6.9)	28.1	26.0	2.1	(1.9; 2.3)
Upper Secondary	273500	283706	(281808; 285603)	-10206	(-12362; -8049)	-3.6	(-4.2; -2.9)	40.9	41.7	-0.7	(-1.0; -0.5)
Post-secondary; Tertiary	186918	194430	(192882; 195979)	-7512	(-9278; -5747)	-3.9	(-4.6; -3.1)	28.0	28.6	-0.6	(-0.8; -0.4)
Unknown	20285	25880	(25306; 26454)	-5595	(-6233; -4956)	-21.6	(-23.3; -19.8)	3.0	3.8	-0.8	(-0.8; -0.7)
**total**	668364	680928		-12564		0.0		100.0	100.0	0.0	
**Highest Parental Education in Detail**											
Primary School or Lower	90285	84584	(83591; 85577)	5701	(4546; 6855)	6.7	(5.5; 8.0)	13.5	12.4	1.1	(0.9; 1.2)
Lower Secondary	149148	141790	(140496; 143083)	7358	(5859; 8857)	5.2	(4.2; 6.2)	22.3	20.8	1.5	(1.3; 1.7)
Upper Secondary	246595	259256	(257435; 261076)	-12661	(-14725; -10596)	-4.9	(-5.5; -4.2)	36.9	38.1	-1.2	(-1.4; -1.0)
Post-secondary; short tertiary	81957	83741	(82727; 84754)	-1784	(-2942; -625)	-2.1	(-3.3; -0.9)	12.3	12.3	0.0	(-0.2; 0.1)
"Profesional"	68265	74102	(73135; 75069)	-5837	(-6931; -4743)	-7.9	(-9.1; -6.7)	10.2	10.9	-0.7	(-0.8; -0.5)
Master's or Doctorate Equivalent	9699	9123	(8791; 9455)	576	(192; 960)	6.3	(2.6; 10.3)	1.5	1.3	0.1	(0.1; 0.2)
Unknown	22415	28176	(27578; 28774)	-5761	(-6427; -5095)	-20.4	(-22.1; -18.7)	3.4	4.1	-0.8	(-0.9; -0.7)
**total**	668364	680771		-12407		0.0		100.0	100.0	0.0	
**Maternal age**											
Below 20	121783	120095	(118921; 121268)	1688	(330; 3046)	1.4	(0.4; 2.4)	18.2	17.6	0.6	(0.5; 0.8)
20-24	190463	195335	(193786; 196883)	-4872	(-6641; -3103)	-2.5	(-3.3; -1.7)	28.5	28.6	-0.1	(-0.3; 0.1)

25-29	166979	171158	(169685; 172632)	-4179	(-5856; -2502)	-2.4	(-3.3; -1.6)	25.0	25.1	-0.1	(-0.3; 0.1)
30-34	111970	114075	(112876; 115273)	-2105	(-3471; -738)	-1.8	(-2.9; -0.8)	16.8	16.7	0.0	(-0.1; 0.2)
Above 34	76710	76979	(75998; 77960)	-269	(-1390; 853)	-0.3	(-1.6; 0.9)	11.5	11.3	0.2	(0.1; 0.3)
Unknown	459	4420	(3989; 4851)	-3961	(-4394; -3528)	-89.6	(-90.5; -88.5)	0.1	0.6	-0.6	(-0.6; -0.5)
**total**	668364	682061		-13697		0.0		100.0	100.0	0.0	
**Parity**											
0	289982	297607	(295729; 299486)	-7625	(-9780; -5470)	-2.6	(-3.2; -1.9)	43.4	43.7	-0.3	(-0.5; -0.1)
1	223514	228359	(226664; 230054)	-4845	(-6777; -2913)	-2.1	(-2.8; -1.4)	33.4	33.5	-0.1	(-0.3; 0.1)
2	94929	97346	(96219; 98474)	-2417	(-3697; -1138)	-2.5	(-3.6; -1.3)	14.2	14.3	-0.1	(-0.2; 0.1)
3	32998	32674	(32024; 33324)	324	(-417; 1065)	1.0	(-1.0; 3.0)	4.9	4.8	0.1	(0.0; 0.2)
4 or more	26874	25123	(24564; 25681)	1751	(1107; 2396)	7.0	(4.6; 9.4)	4.0	3.7	0.3	(0.3; 0.4)
Unknown	67	6	(1; 11)	61	(44; 78)	1020.5	(532.9; 4783.4)	0.0	0.0	0.0	(0.0; 0.0)
**total**	668364	681116		-12752		0.0		100.0	100.0	0.0	

Denmark

Data

For Denmark (2015-2021; n= 423,792 live births), we used individual-level data supplied by Statistics Denmark from the medical birth register (until 2018) and the population registry (2019-2021) and linked parental characteristics (household income, education, maternal age, parity) via birth, population, income, and education registers and aggregated the data to weekly time series (under auspices of project 703566). Because the population registry only includes birth where children are still alive and in country end of year of birth, and we limit the sample to known mothers, we are missing n=4,477 births compared to the official birth count from Statistics Denmark. Quintiles of equivalized household incomes are based on the household income distribution of women aged 15-49 and are lagged by two years before the year of birth. This lag in household income avoids that our estimated compositional change is driven by pandemic-induced change in the income distribution (due to, e.g., income losses) instead of pandemic-induced change in fertility behaviour. To protect anonymity in line with data providers' policies, we set the number of weekly births to 5 if it was positive and below 5. The mother's highest completed formal education was grouped into primary education, upper secondary education, bachelor's degree, and master's and Doctoral degree at year of birth following ISCED-classification.

Results

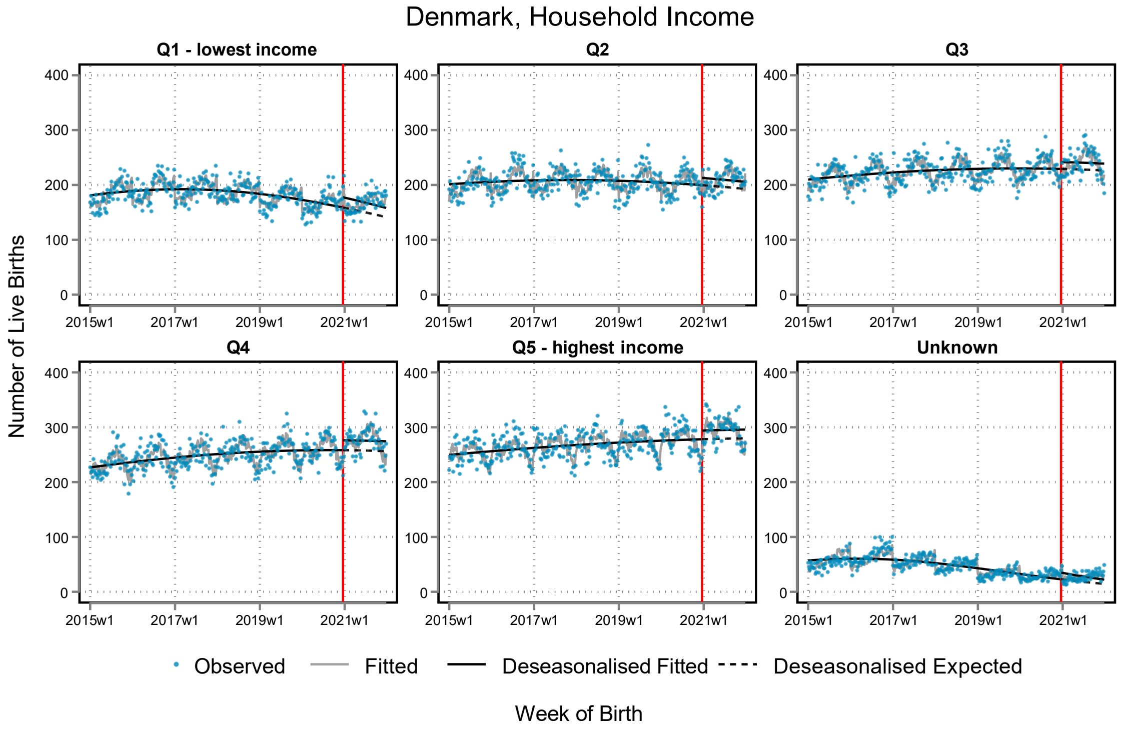

*Supplementary Figure 23: Observed and expected weekly number of live births in **Denmark** by quintile of two-year lagged equivalised household income among women aged 15-49. Expected numbers are estimated by subgroup-specific Poisson regression modes on the full time series including an indicator variable for the exposed period (starting second week of December 2020 to December 2021) to estimate the average effect of the COVID-19 pandemic over the entire period; a linear and a quadratic term for week of live birth to capture potential non-linearities in the secular time trends; week of the year fixed effects to account for seasonality. Weekly counts below 5 were set to 5 to protect anonymity (bottom right panel).*

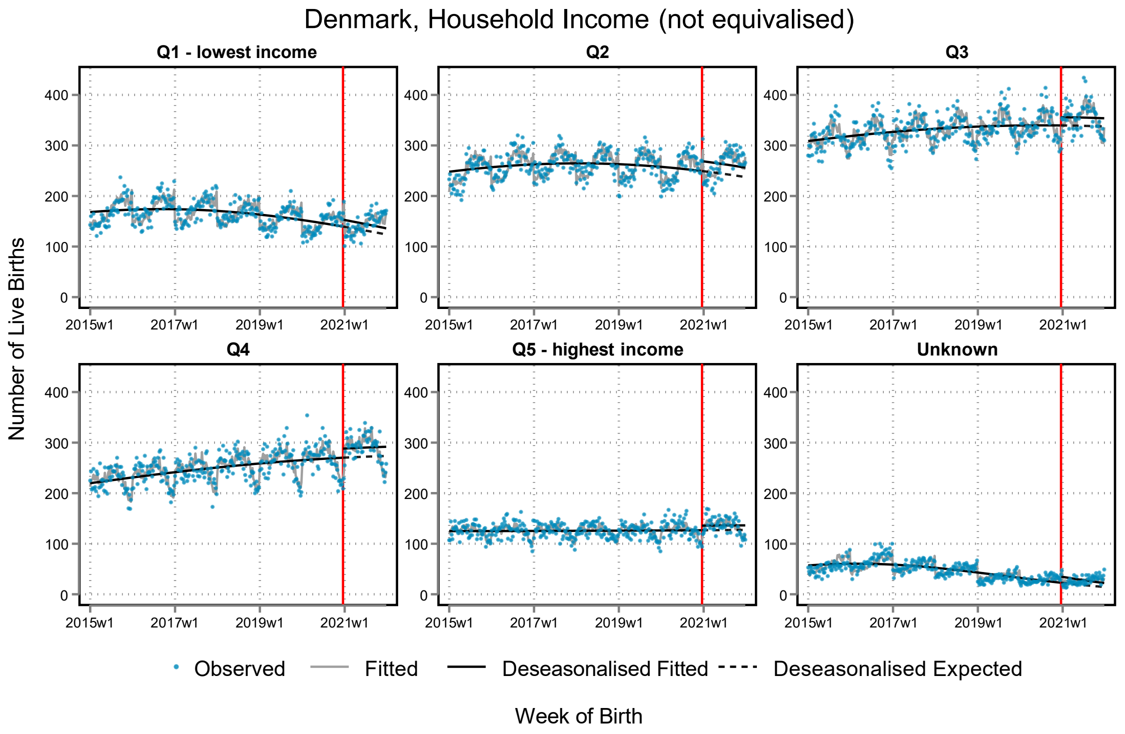

*Supplementary Figure 24: Observed and expected weekly number of live births in **Denmark** by quintile of non-equivalised two-year lagged household income among women aged 15-49. Expected numbers are estimated by subgroup-specific Poisson regression modes on the full time series including an indicator variable for the exposed period (starting second week of December 2020 to December 2021) to estimate the average effect of the COVID-19 pandemic over the entire period; a linear and a quadratic term for week of live birth to capture potential non-linearities in the secular time trends; week of the year fixed effects to account for seasonality. Weekly counts below 5 were set to 5 to protect anonymity (bottom right panel).*

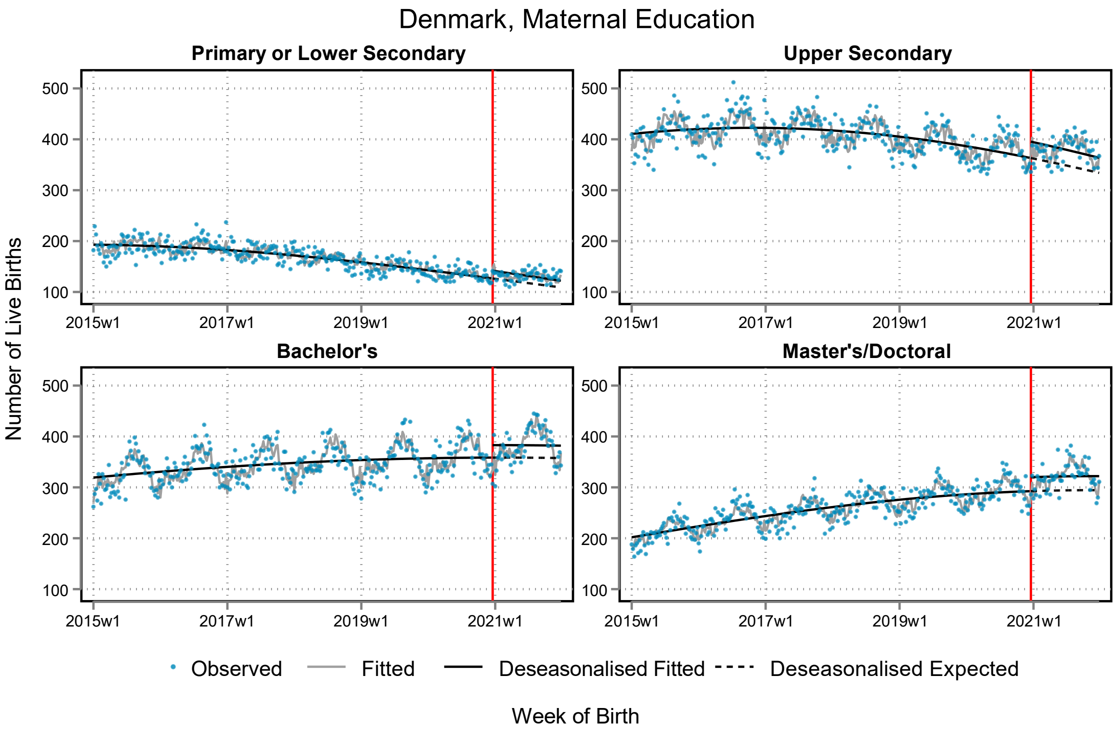

Supplementary Figure 25: Observed and expected weekly number of live births in Denmark by formal maternal education. Expected numbers are estimated by subgroup-specific Poisson regression modes on the full time series including an indicator variable for the exposed period (starting second week of December 2020 to December 2021) to estimate the average effect of the COVID-19 pandemic over the entire period; a linear and a quadratic term for week of live birth to capture potential non-linearities in the secular time trends; week of the year fixed effects to account for seasonality.

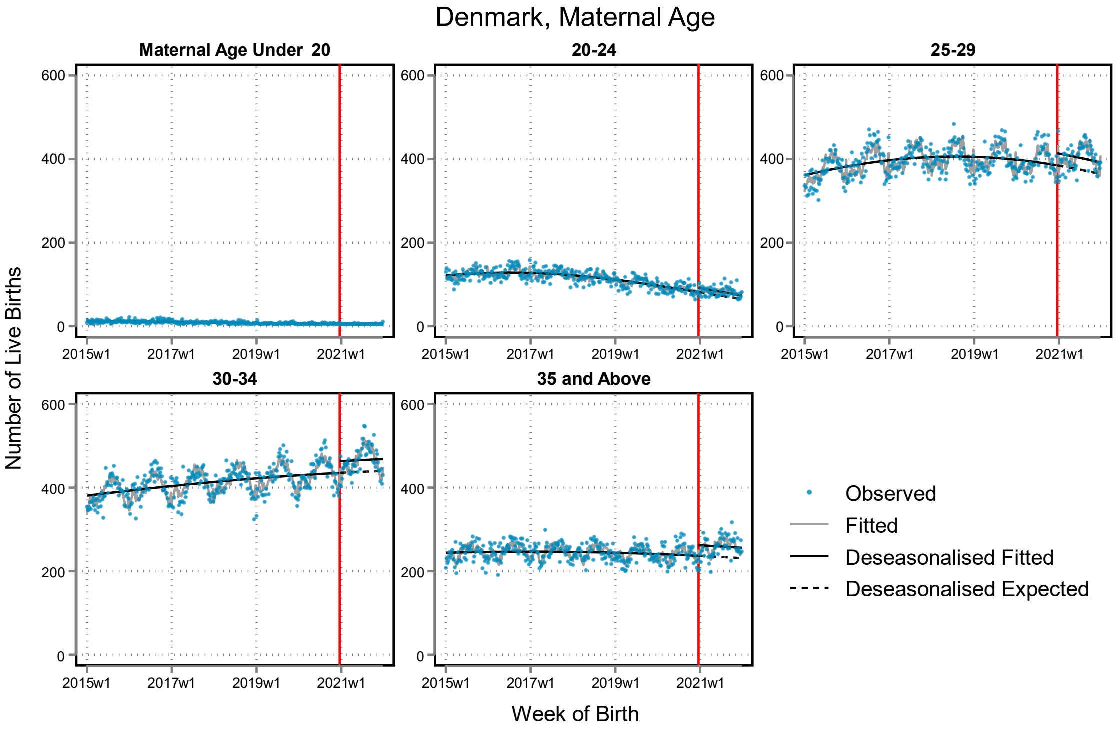

*Supplementary Figure 26: Observed and expected weekly number of live births in **Denmark** by formal maternal age. Expected numbers are estimated by subgroup-specific Poisson regression modes on the full time series including an indicator variable for the exposed period (starting second week of December 2020 to December 2021) to estimate the average effect of the COVID-19 pandemic over the entire period; a linear and a quadratic term for week of live birth to capture potential non-linearities in the secular time trends; week of the year fixed effects to account for seasonality.*

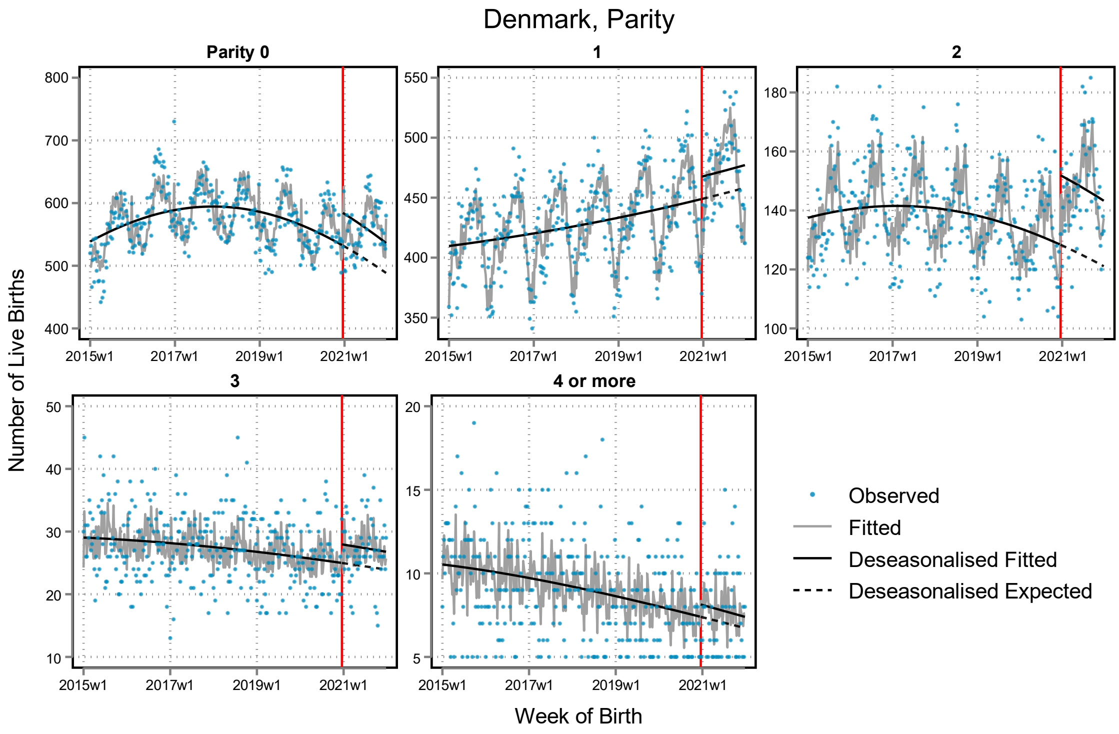

*Supplementary Figure 27: Observed and expected weekly number of live births in **Denmark** by parity. Expected numbers are estimated by subgroup-specific Poisson regression modes on the full time series including an indicator variable for the exposed period (starting second week of December 2020 to December 2021) to estimate the average effect of the COVID-19 pandemic over the entire period; a linear and a quadratic term for week of live birth to capture potential non-linearities in the secular time trends; week of the year fixed effects to account for seasonality.*

Supplementary Table 5: Relative and Percentage Point Differences in the Composition of the December 2020 – December 2021 Birth Cohort in Denmark. "Observed" is abbreviated by "OBS" and "Counterfactual" is abbreviated by "CF". Statistical methods for the estimations are described in the main manuscript.

Characteristic	Observed (OBS) Births	Counterfactual (CF) Births	95%CI: CF Births	OBS - CF Births	95%CI: OBS - CF Births	% more/less than CF	95%CI: % more/less than CF	OBS proportion	CF proportion	OBS - CF proportion	95%CI: OBS - CF proportion
**Non-equivalised household income**											
Lowest 20%	7851	7153	(6889; 7418)	698	(381; 1014)	9.8	(5.8; 14.0)	12.0	11.7	0.2	(-0.2; 0.7)
Q2	14201	13170	(12795; 13546)	1031	(588; 1473)	7.8	(4.8; 11.0)	21.7	21.6	0.1	(-0.5; 0.6)
Q3	19107	18229	(17776; 18682)	878	(350; 1406)	4.8	(2.3; 7.5)	29.2	29.9	-0.8	(-1.4; -0.1)
Q4	15556	14588	(14176; 14999)	968	(489; 1447)	6.6	(3.7; 9.7)	23.7	23.9	-0.2	(-0.8; 0.4)
Highest 20%	7301	6814	(6538; 7090)	487	(164; 810)	7.1	(3.0; 11.7)	11.1	11.2	0.0	(-0.5; 0.4)
Missing income information	1518	991	(912; 1069)	527	(418; 637)	53.2	(42.0; 66.4)	2.3	1.6	0.7	(0.6; 0.8)
Total	65534	60945		4589		7.5		100.0	100.0	0.0	
**Maternal Education**											
Compulsory	7137	6372	(6131; 6612)	765	(473; 1057)	12.0	(7.9; 16.4)	10.9	10.6	0.3	(-0.1; 0.7)
Upper Secondary	20488	18820	(18383; 19256)	1668	(1150; 2187)	8.9	(6.4; 11.4)	31.3	31.2	0.1	(-0.6; 0.7)
Bachelor's Degree	20601	19286	(18818; 19753)	1315	(770; 1861)	6.8	(4.3; 9.5)	31.4	32.0	-0.5	(-1.2; 0.1)
Master's / Doctorate	17308	15827	(15393; 16260)	1481	(977; 1986)	9.4	(6.4; 12.4)	26.4	26.2	0.2	(-0.4; 0.8)
**total**	65534	60304		5230		8.7		100.0	100.0	0.0	
**Maternal age**											
Below 20	291	252	(208; 296)	39	(-16; 95)	15.5	(-1.7; 40.1)	0.4	0.4	0.0	(-0.0; 0.1)
20-24	4481	3952	(3770; 4135)	529	(304; 753)	13.4	(8.4; 18.9)	6.8	6.5	0.3	(0.0; 0.6)
25-29	21752	20231	(19765; 20696)	1521	(973; 2069)	7.5	(5.1; 10.1)	33.2	33.4	-0.2	(-0.8; 0.4)
30-34	25084	23571	(23050; 24092)	1513	(906; 2120)	6.4	(4.1; 8.8)	38.3	38.9	-0.6	(-1.3; 0.0)
Above 34	13960	12608	(12238; 12977)	1352	(916; 1788)	10.7	(7.6; 14.1)	21.3	20.8	0.5	(-0.1; 1.0)
**total**	65568	60613		4955		8.2		100.0	100.0	0.0	
**Parity**											
0	30290	27594	(27061; 28126)	2696	(2064; 3329)	9.8	(7.7; 11.9)	46.2	45.7	0.5	(-0.1; 1.2)
1	25402	24391	(23860; 24922)	1011	(395; 1627)	4.1	(1.9; 6.5)	38.8	40.4	-1.6	(-2.3; -1.0)

2	7960	6727	(6462; 6991)	1233	(916; 1551)	18.3	(13.9; 23.2)	12.1	11.1	1.0	(0.6; 1.4)
3	1475	1319	(1201; 1436)	156	(17; 296)	11.9	(2.7; 22.8)	2.3	2.2	0.1	(-0.1; 0.3)
4 or more	420	382	(321; 443)	38	(-35; 111)	9.9	(-5.1; 30.7)	0.6	0.6	0.0	(-0.1; 0.1)
**total**	65547	60412		5135		8.5		100.0	100.0	0.0	

Ecuador

Data

For Ecuador (2015-2021; n=1,966,277 live births), we used openly available individual-level vital statistics from INEC (Instituto Nacional de Estadística y Censos) (https://aplicaciones3.ecuadorencifras.gob.ec/BIINEC-war/index.xhtml). We used the most recent data (2015-2022), to include births that occurred in 2021 but were registered in 2022. The number of births registered two years after the year of birth has been continuously declining in recent years (https://www.ecuadorencifras.gob.ec/nacidos-vivos-y-defunciones-fetales/). Therefore, the analysed number of live births in 2021 might not be definitive but will be close to the definitive number of live births. For our analysis, results will only be biased by incomplete coverage if the propensity to registration two or more years after birth is associated with parental characteristics. As late registrations will be more likely to happen in remote areas and for births outside hospitals, we believe that, if anything, our estimates for differences in socioeconomic composition will be underestimates.

Data on maternal education available in the vital statistics were inconsistently collected through time and thus not used in our analysis. Instead, we used a deprivation index created to study geographical inequalities in health outcomes.[6] This deprivation index on the canton level (n=221, population size ranges from1,760 to 2,350,278 (median 23,820)) was created using data from the National Population Census 2010 and the National Living Conditions Survey 2013-2014 and comprises 17 indicators in total. We created quintiles of this index and linked them to the canton of maternal residence.

Additionally, we estimated compositional change for maternal age and the number of living children as a measure of parity.

The Zika virus epidemic had two waves in Ecuador (https://www3.paho.org/hq/dmdocuments/2017/2017-phe-zika-situation-report-ecu.pdf). We thus estimated our models once with variables indicating the start of the epidemic in Brazil that coincided with public recommendations to postpone pregnancies in many Latin American countries. Here, we assumed that not the actual ZIKV cases are driving behaviour change, but the spillover of information from other countries and the official recommendations. We then re-estimated our models with variables indicating the 40-weeks lagged period after the two ZIKV waves (1st wave in summer 2016 and 2nd wave in the first half of 2017).

After visual inspection of our results, we decided that the former model specification captured the drop in live births (in some time series clearly visible in the second half of 2016) while the latter specification did not. An exception is the time series for live births with missing information on maternal age, where the second option fitted the data better. The results for the worse performing models with variables indicating the 40-weeks lagged period after the two ZIKV waves (1st wave in summer 2016 and 2nd wave in the first half of 2017) are shown in Supplementary Figures 31 to 33.

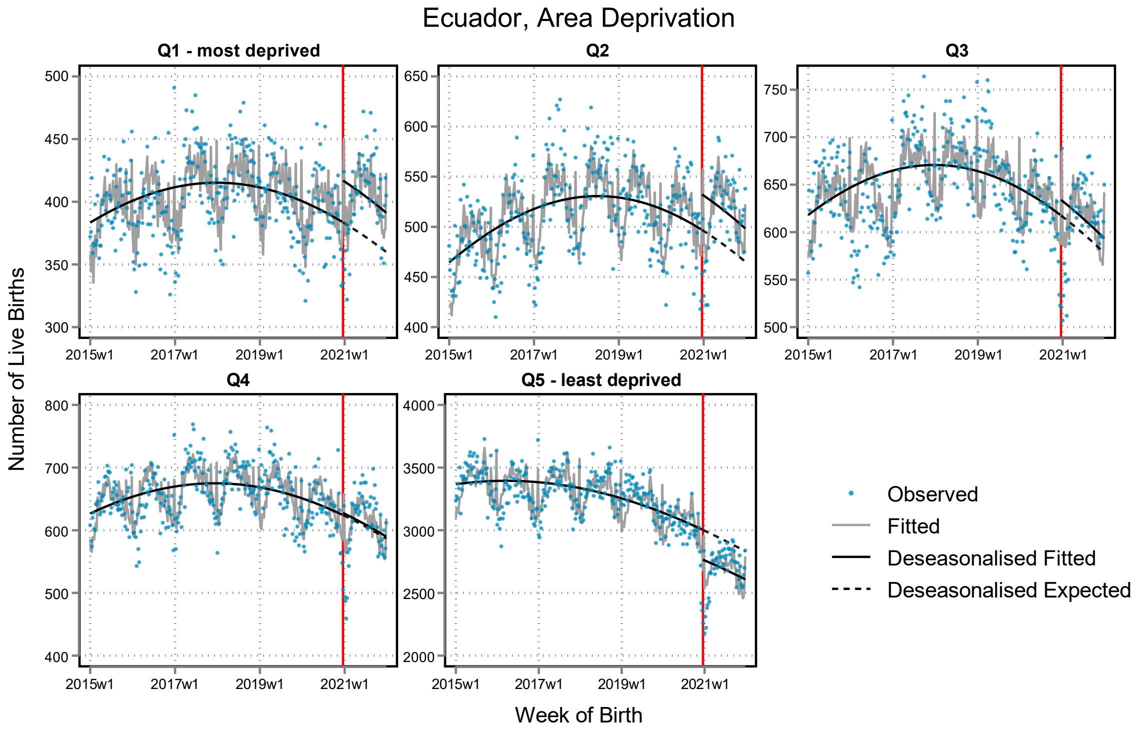

*Supplementary Figure 28: Observed and expected weekly number of live births in **Ecuador** by primary indicator of socioeconomic circumstances (area deprivation in canton of maternal residence). Expected numbers are estimated by subgroup-specific Poisson regression modes on the full time series including an indicator variable for the exposed period (starting second week of December 2020 to December 2021) to estimate the average effect of the COVID-19 pandemic over the entire period; a linear and a quadratic term for week of live birth to capture potential non-linearities in the secular time trends; week of the year fixed effects to account for seasonality; an indicator variable (August 2016-December 2016) to account for the 2015-2016 Zika virus epidemic.*

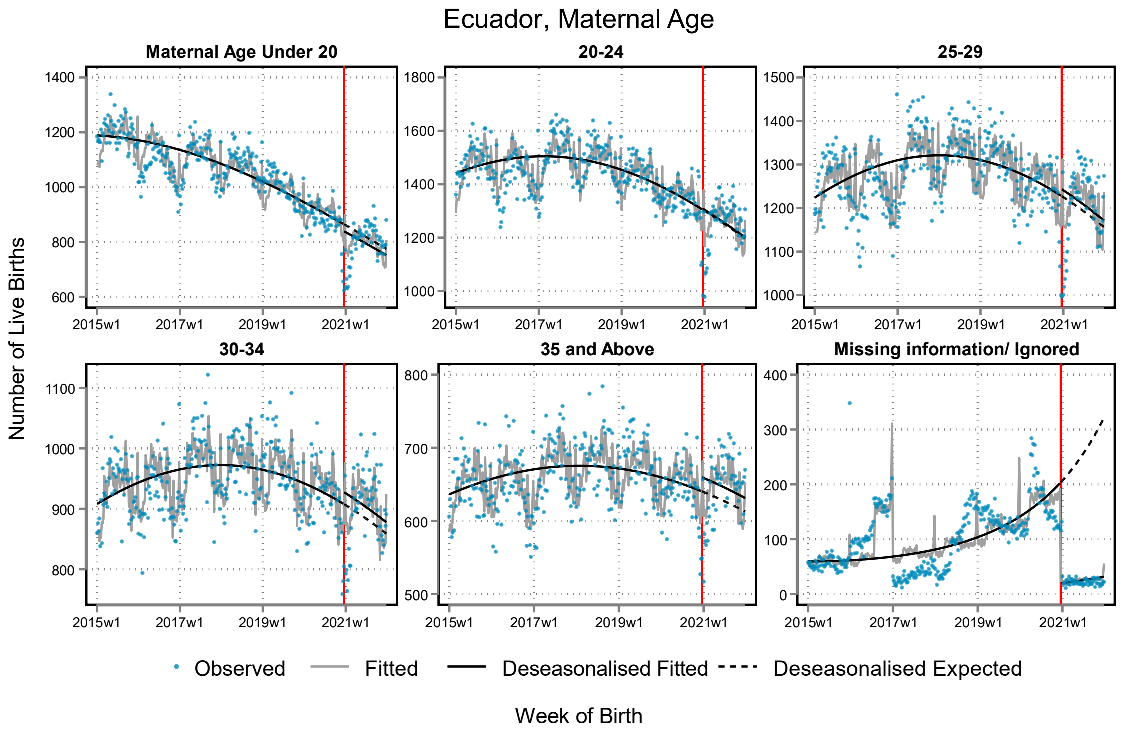

*Supplementary Figure 29: Observed and expected weekly number of live births in **Ecuador** by maternal age. Expected numbers are estimated by subgroup-specific Poisson regression modes on the full time series including an indicator variable for the exposed period (starting second week of December 2020 to December 2021) to estimate the average effect of the COVID-19 pandemic over the entire period; a linear and a quadratic term for week of live birth to capture potential non-linearities in the secular time trends; week of the year fixed effects to account for seasonality; an indicator variable (August 2016-December 2016) to account for the 2015-2016 Zika virus epidemic.*

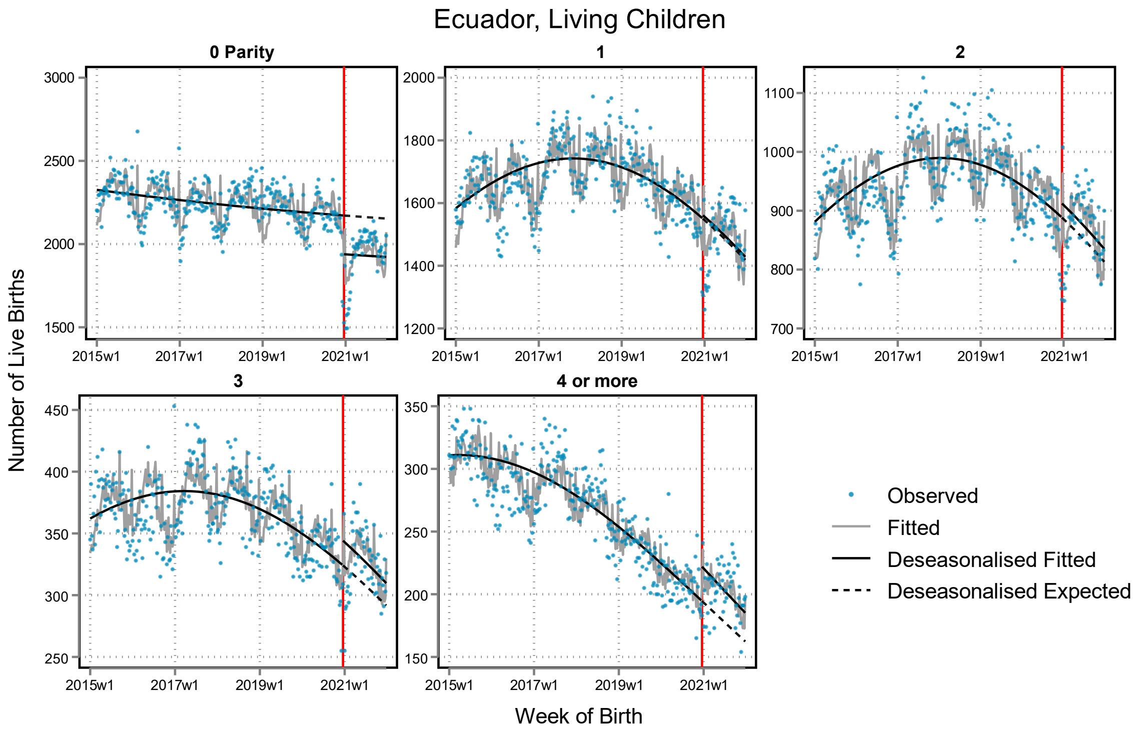

Supplementary Figure 30: Observed and expected weekly number of live births in ***Ecuador*** *by number of living children (before focal birth). Expected numbers are estimated by subgroup-specific Poisson regression modes on the full time series including an indicator variable for the exposed period (starting December 2020 to December 2021) to estimate the average effect of the COVID-19 pandemic over the entire period; a linear and a quadratic term for week of live birth to capture potential non-linearities in the secular time trends; week of the year fixed effects to account for seasonality; an indicator variable (August 2016-December 2016) to account for the 2015-2016 Zika virus epidemic.*

Supplementary Table 6: Relative and Percentage Point Differences in the Composition of the December 2020 – December 2021 Birth Cohort in Ecuador. "Observed" is abbreviated by "OBS" and "Counterfactual" is abbreviated by "CF". Statistical methods for the estimations are described in the main manuscript.

Characteristic	Observed (OBS) Births	Counterfactual (CF) Births	95%CI: CF Births	OBS - CF Births	95%CI: OBS - CF Births	% more/less than CF	95%CI: % more/less than CF	OBS proportion	CF proportion	OBS - CF proportion	95%CI: OBS - CF proportion
**Maternal age**											
Below 20	42974	44284	(43615; 44953)	-1310	(-2092; -527)	-3.0	(-4.4; -1.5)	16.5	16.3	0.2	(-0.1; 0.4)
20-24	67789	67584	(66736; 68433)	205	(-785; 1194)	0.3	(-0.9; 1.6)	26.0	24.8	1.1	(0.8; 1.4)
25-29	65310	64477	(63632; 65323)	833	(-150; 1816)	1.3	(0.0; 2.6)	25.0	23.7	1.3	(1.0; 1.6)
30-34	48797	47759	(47030; 48487)	1038	(191; 1886)	2.2	(0.6; 3.8)	18.7	17.6	1.1	(0.9; 1.4)
Above 34	34893	33876	(33259; 34493)	1017	(299; 1735)	3.0	(1.2; 4.9)	13.4	12.5	0.9	(0.7; 1.1)
Unknown	1383	14086	(13491; 14681)	-12703	(-13303; -12104)	-90.2	(-90.6; -89.7)	0.5	5.2	-4.6	(-4.9; -4.4)
**total**	261146	272066		-10920		0.0		100.0	100.0	0.0	
**Parity**											
0	104357	116884	(115718; 118049)	-12527	(-13853; -11200)	-10.7	(-11.6; -9.8)	40.0	43.4	-3.4	(-3.7; -3.1)
1	80878	80310	(79381; 81239)	568	(-515; 1651)	0.7	(-0.4; 1.9)	31.0	29.8	1.2	(0.9; 1.5)
2	47243	46003	(45299; 46707)	1240	(417; 2063)	2.7	(1.1; 4.3)	18.1	17.1	1.0	(0.8; 1.3)
3	17681	16650	(16234; 17066)	1031	(540; 1522)	6.2	(3.6; 8.9)	6.8	6.2	0.6	(0.4; 0.7)
4 or more	10987	9630	(9333; 9926)	1357	(997; 1718)	14.1	(10.7; 17.7)	4.2	3.6	0.6	(0.5; 0.7)
**total**	261146	269477		-8331		0.0		100.0	100.0	0.0	

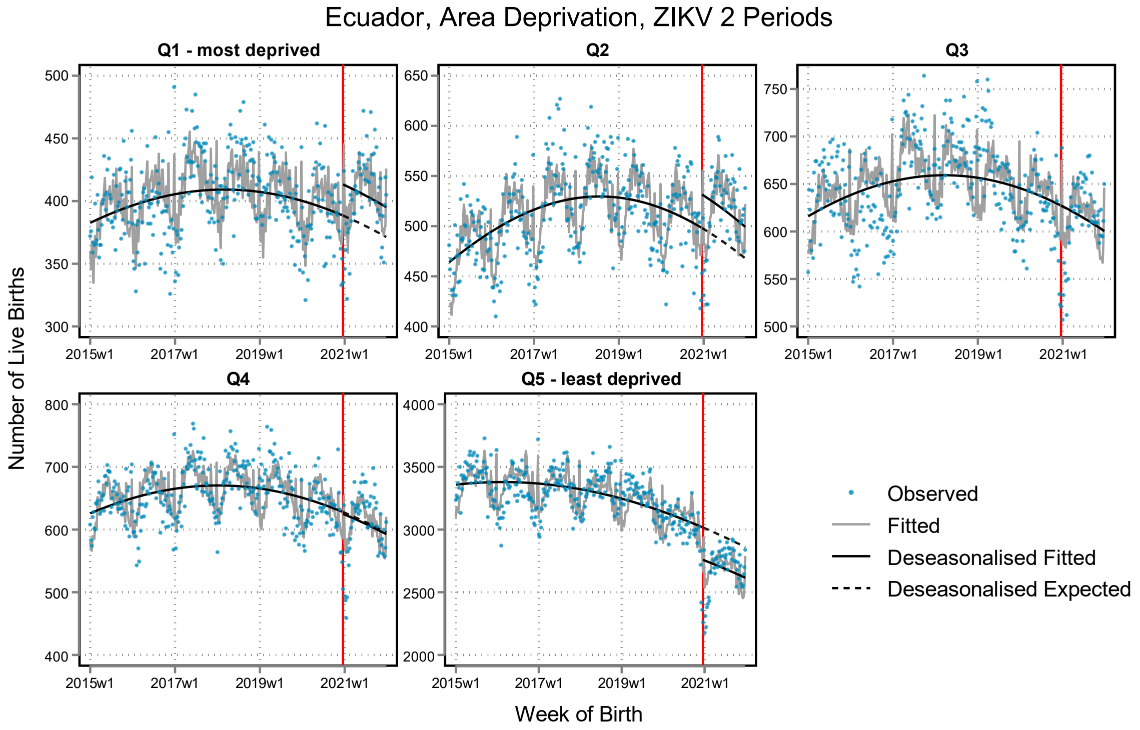

*Supplementary Figure 31: Observed and expected weekly number of live births in **Ecuador** by primary indicator of socioeconomic circumstances (area deprivation in canton of maternal residence). Expected numbers are estimated by subgroup-specific Poisson regression modes on the full time series including an indicator variable for the exposed period (starting second week of December 2020 to December 2021) to estimate the average effect of the COVID-19 pandemic over the entire period; a linear and a quadratic term for week of live birth to capture potential non-linearities in the secular time trends; week of the year fixed effects to account for seasonality; To account for the 2015-2016 Zika virus epidemic in this sensitivity analysis, we used two variables indicating the 40-week lagged periods after the two ZIKV waves in Ecuador (1st wave in summer 2016 and 2nd wave in the first half of 2017) from the fifth to the 28th (including) calendar week of 2017 and the period from the 43rd calendar week of 2017 to the 18th calendar week of 2018.*

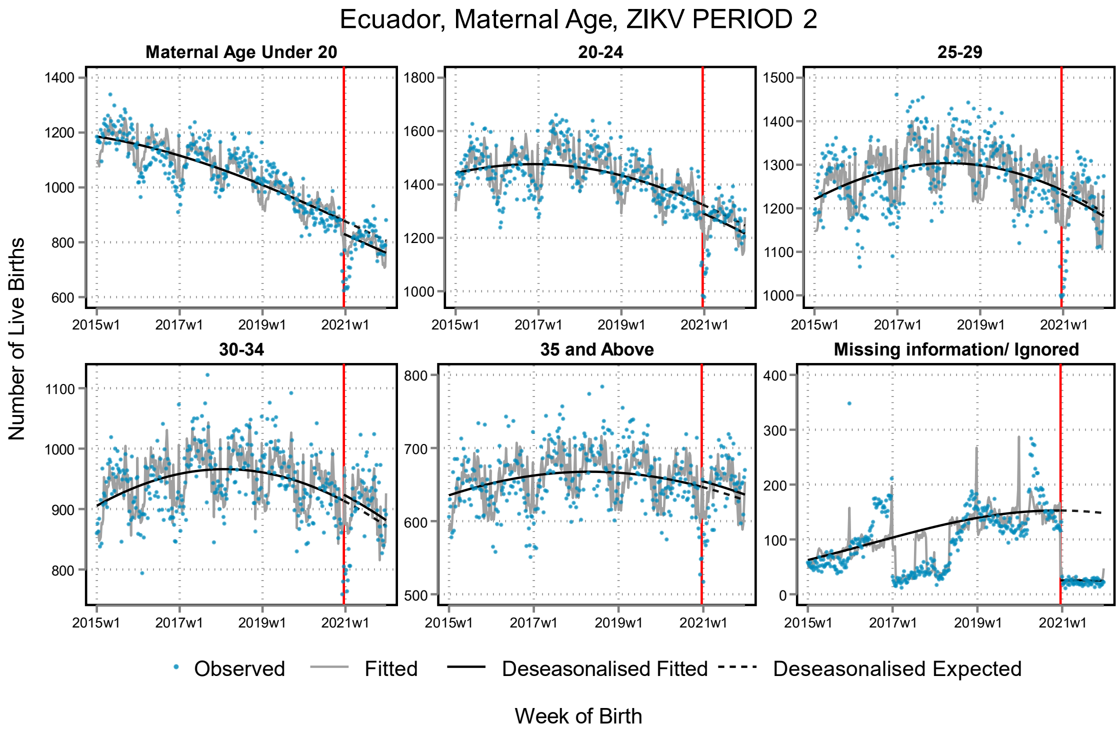

Supplementary Figure 32: Observed and expected weekly number of live births in ***Ecuador*** *by maternal age. Expected numbers are estimated by subgroup-specific Poisson regression modes on the full time series including an indicator variable for the exposed period (starting second week of December 2020 to December 2021) to estimate the average effect of the COVID-19 pandemic over the entire period; a linear and a quadratic term for week of live birth to capture potential non-linearities in the secular time trends; week of the year fixed effects to account for seasonality; To account for the 2015-2016 Zika virus epidemic in this sensitivity analysis, we used two variables indicating the 40-week lagged periods after the two ZIKV waves in Ecuador (1st wave in summer 2016 and 2nd wave in the first half of 2017) from the fifth to the 28th (including) calendar week of 2017 and the period from the 43rd calendar week of 2017 to the 18th calendar week of 2018.*

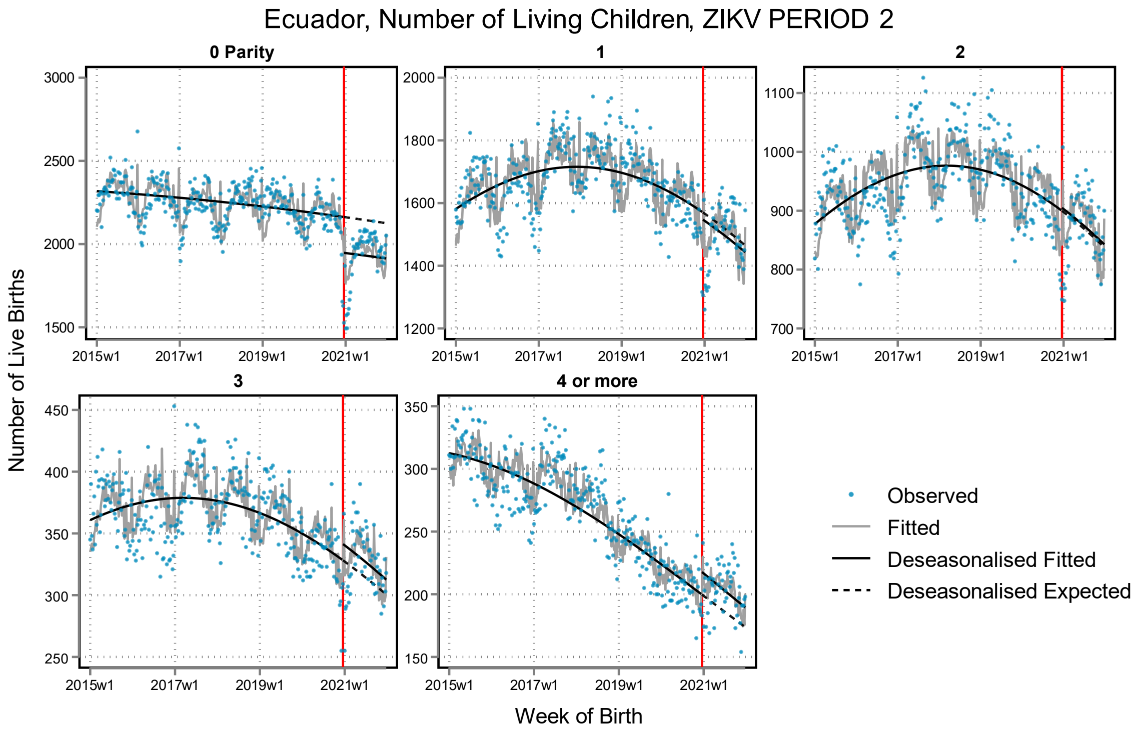

Supplementary Figure 33: Observed and expected weekly number of live births in ***Ecuador*** *by by number of living children (before focal birth). Expected numbers are estimated by subgroup-specific Poisson regression modes on the full time series including an indicator variable for the exposed period (starting second week of December 2020 to December 2021) to estimate the average effect of the COVID-19 pandemic over the entire period; a linear and a quadratic term for week of live birth to capture potential non-linearities in the secular time trends; week of the year fixed effects to account for seasonality; To account for the 2015-2016 Zika virus epidemic in this sensitivity analysis, we used two variables indicating the 40-week lagged periods after the two ZIKV waves in Ecuador (1st wave in summer 2016 and 2nd wave in the first half of 2017) from the fifth to the 28th (including) calendar week of 2017 and the period from the 43rd calendar week of 2017 to the 18th calendar week of 2018.*

England

Data

For England (2015-2021; n=4,391,999 live births), we purchased monthly time series (2015-2022) of the number of live births by deciles of the Index for Multiple Deprivation (IMD) from the Office for National Statistics. These data are now openly available due to our purchase.

(https://www.ons.gov.uk/peoplepopulationandcommunity/birthsdeathsandmarriages/livebirths/adhocs/1703livebirthsbymonthofoccurrenceandimddecileenglandandwales2015to2022)

These data are considered to cover 100% of live births and there are only a low number of late registrations which, for 2021, are already captured in our data.

The IMD is created for Lower Super Output Areas (LSOAs) of mothers' residence. IMD deciles for 2015 are based on 2015 census data and IMD deciles for 2016-2022 are based on 2019 census data.

We use quintiles of the IMD as primary indicator of socioeconomic circumstances but show results also for deciles.

Results

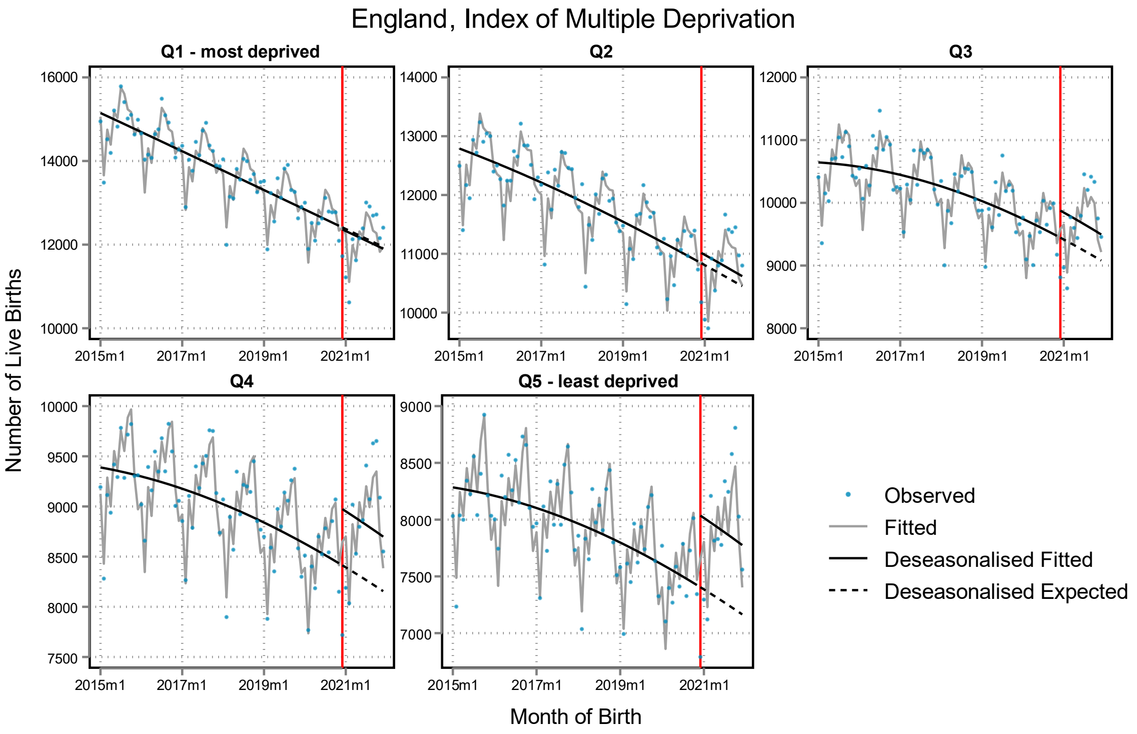

*Supplementary Figure 31: Observed and expected monthly number of live births in **England** by primary indicator of socioeconomic circumstances (area deprivation in Lower Super Output Areas of maternal residence). Expected numbers are estimated by subgroup-specific Poisson regression modes on the full time series including an indicator variable for the exposed period (starting December 2020 to December 2021) to estimate the average effect of the COVID-19 pandemic over the entire period; a linear and a quadratic term for month of live birth to capture potential non-linearities in the secular time trends; month of the year fixed effects to account for seasonality.*

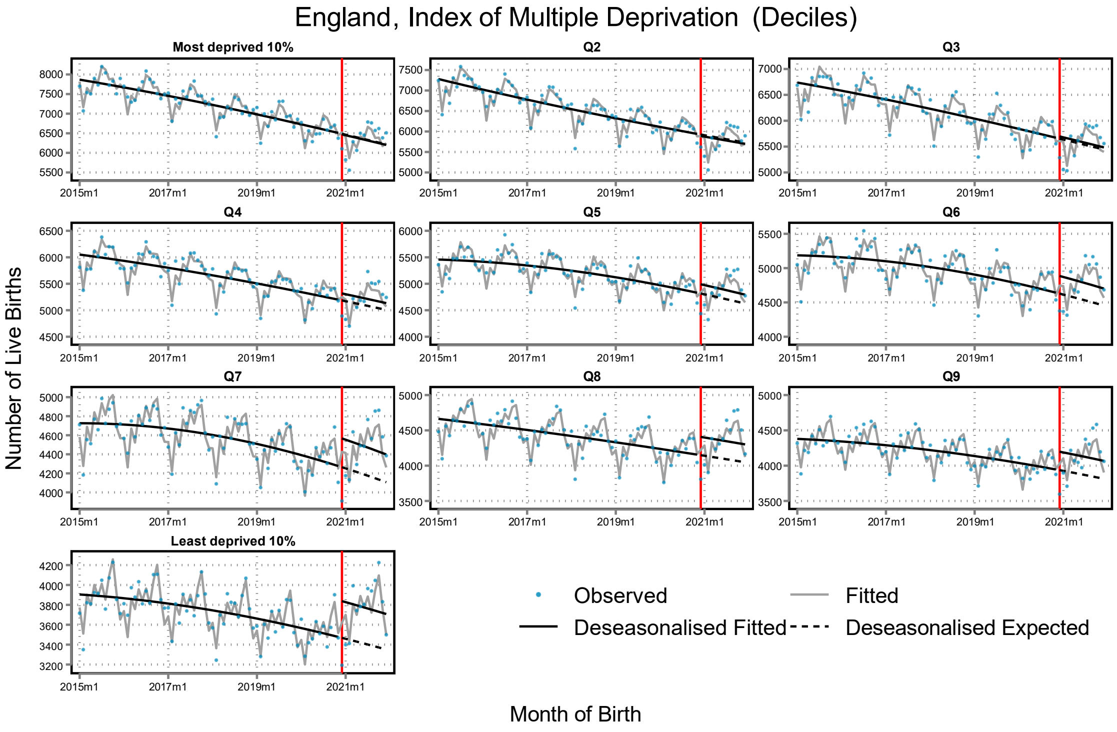

Supplementary Figure 32: Observed and expected monthly number of live births in ***England*** *by decile of the index of multiple deprivation of Lower Super Output Areas of maternal residence. Expected numbers are estimated by subgroup-specific Poisson regression modes on the full time series including an indicator variable for the exposed period (starting December 2020 to December 2021) to estimate the average effect of the COVID-19 pandemic over the entire period; a linear and a quadratic term for month of live birth to capture potential non-linearities in the secular time trends; month of the year fixed effects to account for seasonality.*

Supplementary Table 7: Relative and Percentage Point Differences in the Composition of the December 2020 – December 2021 Birth Cohort in England. "Observed" is abbreviated by "OBS" and "Counterfactual" is abbreviated by "CF". Statistical methods for the estimations are described in the main manuscript.

Characteristic	Observed (OBS) Births	Counterfactual (CF) Births	95%CI: CF Births	OBS - CF Births	95%CI: OBS - CF Births	% more/less than CF	95%CI: % more/less than CF	OBS proportion	CF proportion	OBS - CF proportion	95%CI: OBS - CF proportion
**Index of Multiple Deprivation**											
D1 - Most Deprived	82428	82610	(81646; 83574)	-182	(-1298; 934)	-0.2	(-1.4; 1.0)	12.9	13.4	-0.5	(-0.6; -0.3)
D2	75324	75750	(74824; 76676)	-426	(-1497; 645)	-0.6	(-1.8; 0.7)	11.8	12.2	-0.5	(-0.6; -0.4)
D3	72583	72036	(71134; 72938)	547	(-498; 1592)	0.8	(-0.5; 2.0)	11.3	11.6	-0.3	(-0.5; -0.2)
D4	67869	66179	(65312; 67046)	1690	(684; 2696)	2.6	(1.2; 3.9)	10.6	10.7	-0.1	(-0.2; 0.0)
D5	63451	61232	(60400; 62064)	2219	(1251; 3187)	3.6	(2.2; 5.1)	9.9	9.9	0.0	(-0.1; 0.1)
D6	62186	58909	(58091; 59726)	3277	(2325; 4230)	5.6	(4.1; 7.0)	9.7	9.5	0.2	(0.1; 0.3)
D7	58135	54293	(53508; 55078)	3842	(2926; 4758)	7.1	(5.6; 8.6)	9.1	8.8	0.3	(0.2; 0.4)
D8	56418	53106	(52326; 53886)	3312	(2403; 4220)	6.2	(4.7; 7.8)	8.8	8.6	0.2	(0.1; 0.3)
D9	53562	50222	(49466; 50979)	3340	(2458; 4222)	6.6	(5.1; 8.3)	8.4	8.1	0.2	(0.1; 0.4)
D10 - Least Deprived	48832	44133	(43427; 44839)	4699	(3871; 5527)	10.6	(8.9; 12.4)	7.6	7.1	0.5	(0.4; 0.6)
**total**	640788	618470		22318		0.0		100.0	100.0	0.0	

Finland

Data

For Finland (2015-2021; n= 336,494 live births), we used individual-level data from the medical birth register and linked parental characteristics (household income, education, maternal age, parity) via population registers and aggregated the data to weekly time series (access permit: TK/1170/07.03.00/2023 and THL/6303/14.06.00/2023). Quintiles of household incomes are based on the household income distribution of women aged 15-49 and are lagged by two years before the year of birth. This lag in household income avoids that our estimated compositional change is driven by pandemic-induced change in the income distribution (due to, e.g., income losses) instead of pandemic-induced change in fertility behaviour. To protect anonymity in line with data providers' policies, we set the number of weekly births to 5 if it was below 5.

The mother's highest completed formal education was grouped into primary education, upper secondary education, bachelor's degree, and master's and Doctoral degree and was lagged by one year before birth due to data availability.

We additionally estimated compositional change along maternal age and parity.

Results

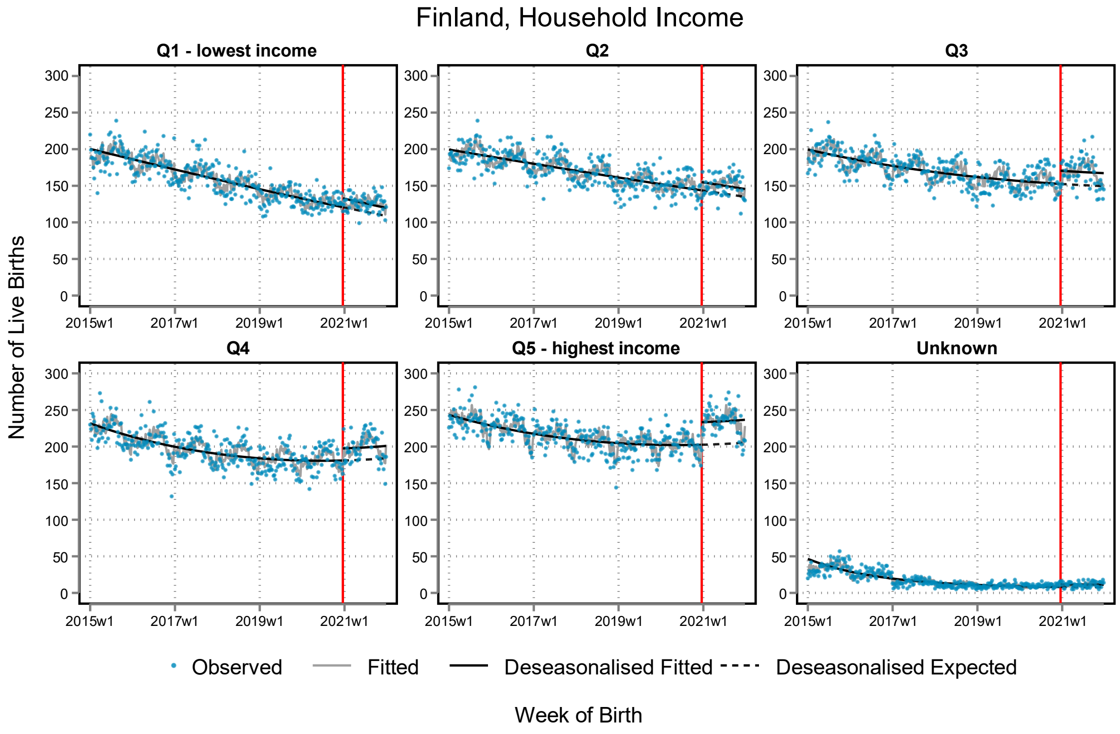

*Supplementary Figure 33: Observed and expected weekly number of live births in **Finland** by quintile of equivalised disposable household income among women aged 15-49. Expected numbers are estimated by subgroup-specific Poisson regression modes on the full time series including an indicator variable for the exposed period (starting second week of December 2020 to December 2021) to estimate the average effect of the COVID-19 pandemic over the entire period; a linear and a quadratic term for week of live birth to capture potential non-linearities in the secular time trends; week of the year fixed effects to account for seasonality. Weekly counts below 5 were set to 5 to protect anonymity (bottom right panel).*

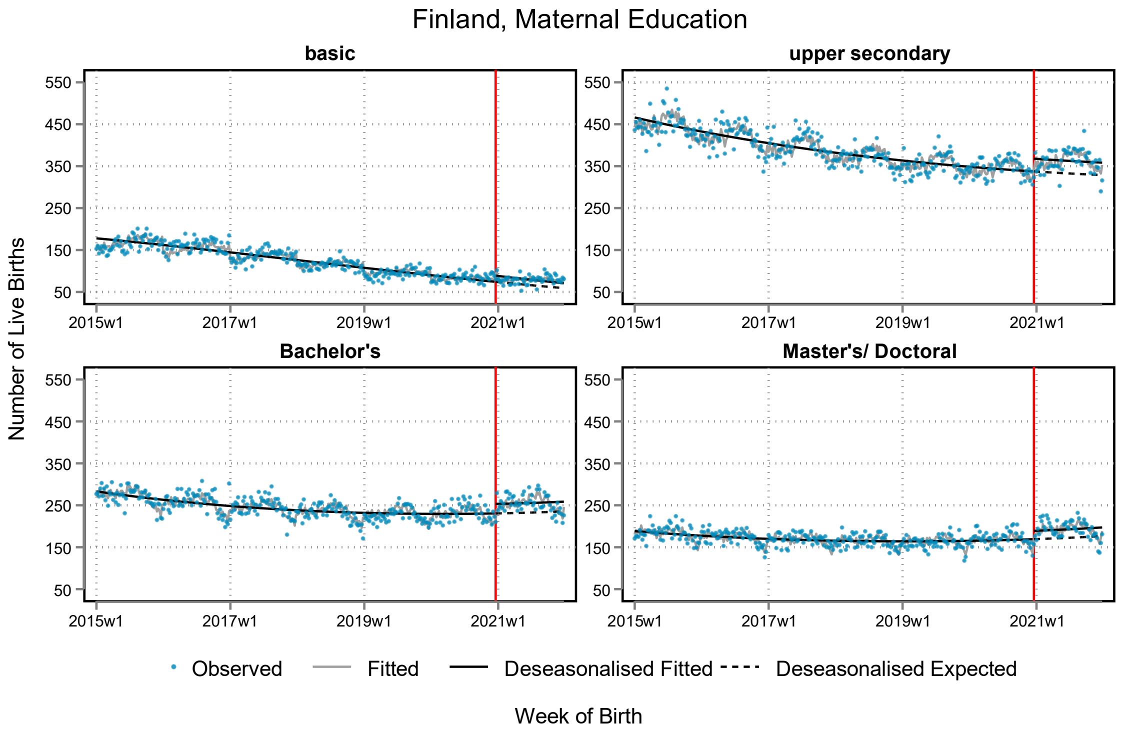

Supplementary *Figure 34: Observed and expected weekly number of live births in **Finland** by formal maternal education. Expected numbers are estimated by subgroup-specific Poisson regression modes on the full time series including an indicator variable for the exposed period (starting second week of December 2020 to December 2021) to estimate the average effect of the COVID-19 pandemic over the entire period; a linear and a quadratic term for week of live birth to capture potential non-linearities in the secular time trends; week of the year fixed effects to account for seasonality.*

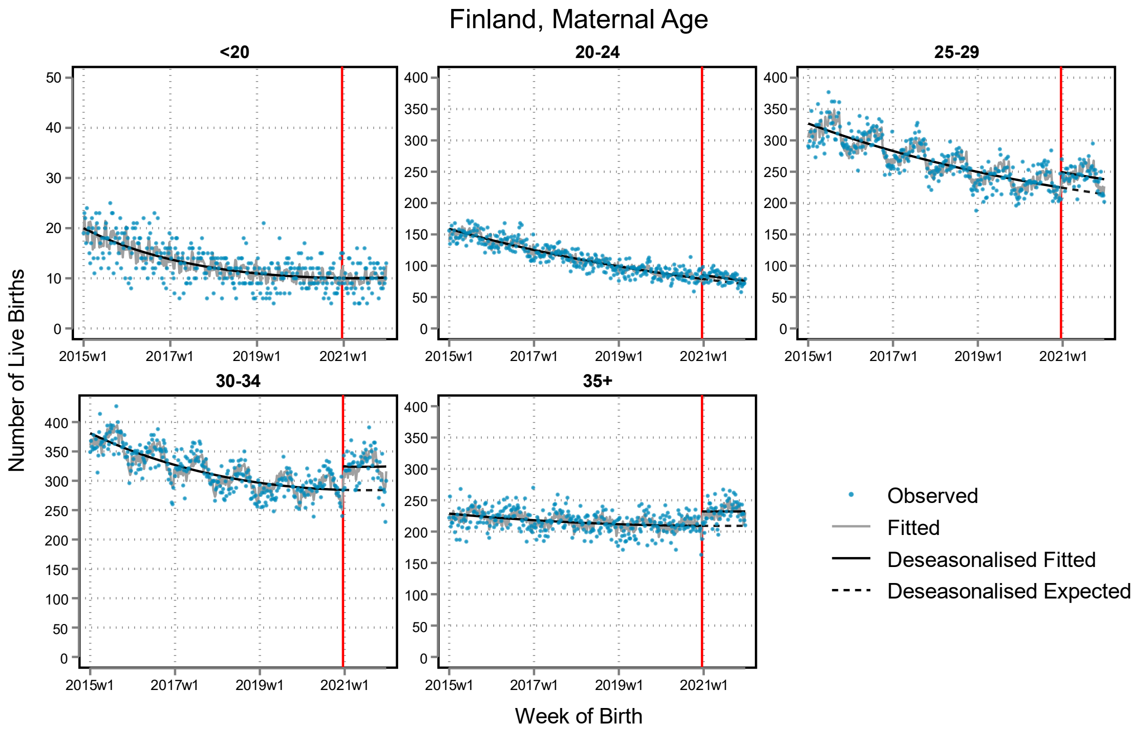

Supplementary Figure 35: Observed and expected weekly number of live births in ***Finland*** *by maternal age. Expected numbers are estimated by subgroup-specific Poisson regression modes on the full time series including an indicator variable for the exposed period (starting second week of December 2020 to December 2021) to estimate the average effect of the COVID-19 pandemic over the entire period; a linear and a quadratic term for week of live birth to capture potential non-linearities in the secular time trends; week of the year fixed effects to account for seasonality. Weekly counts below 5 were set to 5 to protect anonymity.*

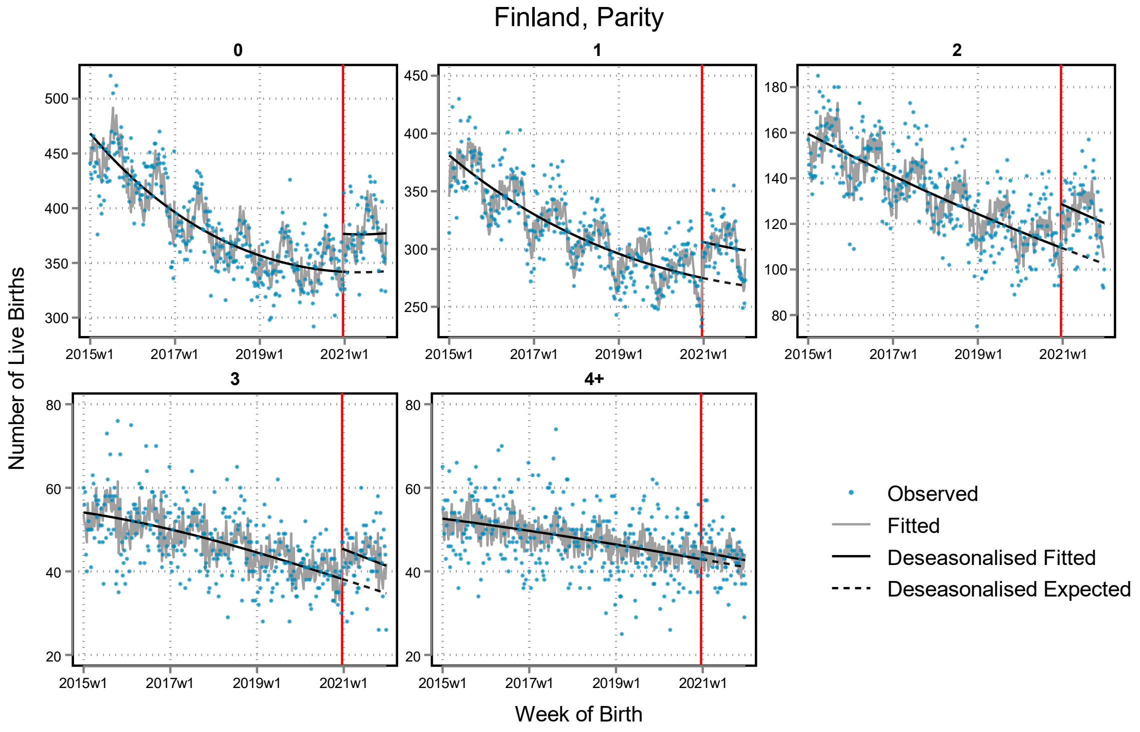

*Supplementary Figure 36: Observed and expected weekly number of live births in **Finland** by parity. Expected numbers are estimated by subgroup-specific Poisson regression modes on the full time series including an indicator variable for the exposed period (starting second week of December 2020 to December 2021) to estimate the average effect of the COVID-19 pandemic over the entire period; a linear and a quadratic term for week of live birth to capture potential non-linearities in the secular time trends; week of the year fixed effects to account for seasonality.*

Supplementary Table 8: Relative and Percentage Point Differences in the Composition of the December 2020 – December 2021 Birth Cohort in Finland. "Observed" is abbreviated by "OBS" and "Counterfactual" is abbreviated by "CF". Statistical methods for the estimations are described in the main manuscript.

Characteristic	Observed (OBS) Births	Counterfactual (CF) Births	95%CI: CF Births	OBS - CF Births	95%CI: OBS - CF Births	% more/less than CF	95%CI: % more/less than CF	OBS proportion	CF proportion	OBS - CF proportion	95%CI: OBS - CF proportion
**Maternal Education**											
Compulsory	4298	3590	(3422; 3758)	708	(497; 919)	19.7	(14.4; 25.6)	9.0	8.3	0.7	(0.3; 1.1)
Upper Secondary	19563	17947	(17516; 18378)	1616	(1105; 2127)	9.0	(6.4; 11.7)	40.8	41.4	-0.6	(-1.4; 0.1)
Bachelor's Degree	13747	12517	(12146; 12889)	1230	(793; 1666)	9.8	(6.7; 13.2)	28.6	28.9	-0.2	(-1.0; 0.5)
Master's / Doctorate	10376	9286	(8961; 9611)	1090	(708; 1471)	11.7	(8.0; 15.8)	21.6	21.4	0.2	(-0.5; 0.9)
**total**	47984	43341		4643		0.1		100.0	100.0	0.0	
**Maternal age**											
Below 20	544	546	(471; 621)	-2	(-90; 85)	-0.4	(-12.4; 15.4)	1.1	1.3	-0.1	(-0.3; 0.0)
20-24	4352	4036	(3845; 4227)	316	(86; 546)	7.8	(3.0; 13.2)	9.1	9.4	-0.3	(-0.7; 0.1)
25-29	13119	11814	(11469; 12158)	1305	(894; 1717)	11.0	(7.9; 14.4)	27.3	27.5	-0.1	(-0.8; 0.5)
30-34	17453	15307	(14904; 15711)	2146	(1666; 2625)	14.0	(11.1; 17.1)	36.4	35.6	0.8	(-0.0; 1.5)
Above 34	12516	11278	(10926; 11629)	1238	(824; 1653)	11.0	(7.6; 14.6)	26.1	26.2	-0.2	(-0.8; 0.5)
**total**	47984	42981		5003		0.1		100.0	100.0	0.0	
**Parity**											
0	20304	18437	(17994; 18880)	1867	(1343; 2391)	10.1	(7.5; 12.8)	42.3	42.9	-0.6	(-1.4; 0.2)
1	16286	14624	(14235; 15014)	1662	(1199; 2124)	11.4	(8.5; 14.4)	33.9	34.0	-0.1	(-0.8; 0.7)
2	6694	5697	(5461; 5933)	997	(711; 1283)	17.5	(12.8; 22.6)	14.0	13.3	0.7	(0.2; 1.2)
3	2340	1967	(1829; 2104)	373	(206; 540)	19.0	(11.2; 27.9)	4.9	4.6	0.3	(-0.0; 0.6)
4 or more	2360	2269	(2116; 2423)	91	(-90; 271)	4.0	(-2.6; 11.6)	4.9	5.3	-0.4	(-0.7; -0.0)
**total**	47984	42994		4990		0.1		100.0	100.0	0.0	

Mexico

Data

For Mexico (2015-2021, n=13,788,132 live births), we used openly available individual-level data collected with birth certificates issued by the Civil Registry and provided through the Instituto Nactional de Estadística y Geografía (INEGI).
(https://en.www.inegi.org.mx/programas/natalidad/#microdata)

Like for Ecuador, we used the most recent data (2015-2022), to include births that occurred in 2021 but were registered in 2022. Still, it is likely that these data do not capture all births that occurred in Mexico 2015-2021. However, the difference between registered and occurred births decreased slightly over the last years. While 1,616,988 out of 2,2412,558 babies born in 2015 were also registered in 2015 (the remaining births were registered 2016-2022), 1,432,072 out of 1,892,086 babies born in 2019 were also registered in 2019. With only one additional year of late registrations for the 2021 birth cohort, our number of live births for 2021 will be lower than the actual number of live births in 2021.

As mentioned for Ecuador, results will only be biased by incomplete coverage if the propensity to registration two or more years after birth is associated with parental characteristics. As late registrations will be more likely to happen in remote areas and for births outside hospitals, we believe that, if anything, our estimates for differences in socioeconomic composition will be underestimates. For example, in figure S31 and S32, we see that the number of live births remains unchanged in the exposed period for parents with elementary and primary education while there were fewer births among parents with upper secondary education. If late registration of live births was more common among lower educated parents, the difference between observed and expected number of live births would be higher and the socioeconomic composition of the December 2020 – December 2021 birth cohort therefore more disadvantaged compared with a counterfactual birth cohort than estimated.

The data collection did not allow us to form a subgroup with post-secondary or tertiary education. Parental education was collected through 8 levels: 1) no schooling, 2) 1-3 years of elementary, 3) 4-5 years of elementary, 4) completed elementary school, 5) lower secondary school, 6) upper secondary, 7) vocational upper secondary, and 8) other. According to OECD's profile of Mexico's educational system, these levels do not go beyond ISCED 3 levels (https://gpseducation.oecd.org/CountryProfile?primaryCountry=MEX&treshold=10&topic=EO). As this did not allow us to create groups similar to the other countries, our primary indicator for socioeconomic circumstances were 3 education levels: 1,2,3,4 to "elementary", level 5 as lower secondary education, levels 6,7,8 to "upper secondary".

Additionally, we estimated compositional change for highest parental education (using paternal education if maternal education was missing), maternal education in more detail, maternal age, and parity.

In Mexico, the ZIKV epidemic started and peaked in the second half of 2016.[7] If change in childbearing behaviour would respond to the number of cases, we would expect to see changes in the number of live births starting in the first half of 2017. However, like in Ecuador, using the indicator variable for an earlier period (August 2016 – December 2016) in accordance with the ZIKV epidemic in Brazil and official recommendations to postpone pregnancy in many Latin American countries, captured changes

in the number of live births better. For an example, see the time series for missing information on maternal education in figure S31 (bottom right panel), where this model specification captures the increase in live births with missing information in the second half of 2016 well.

Results

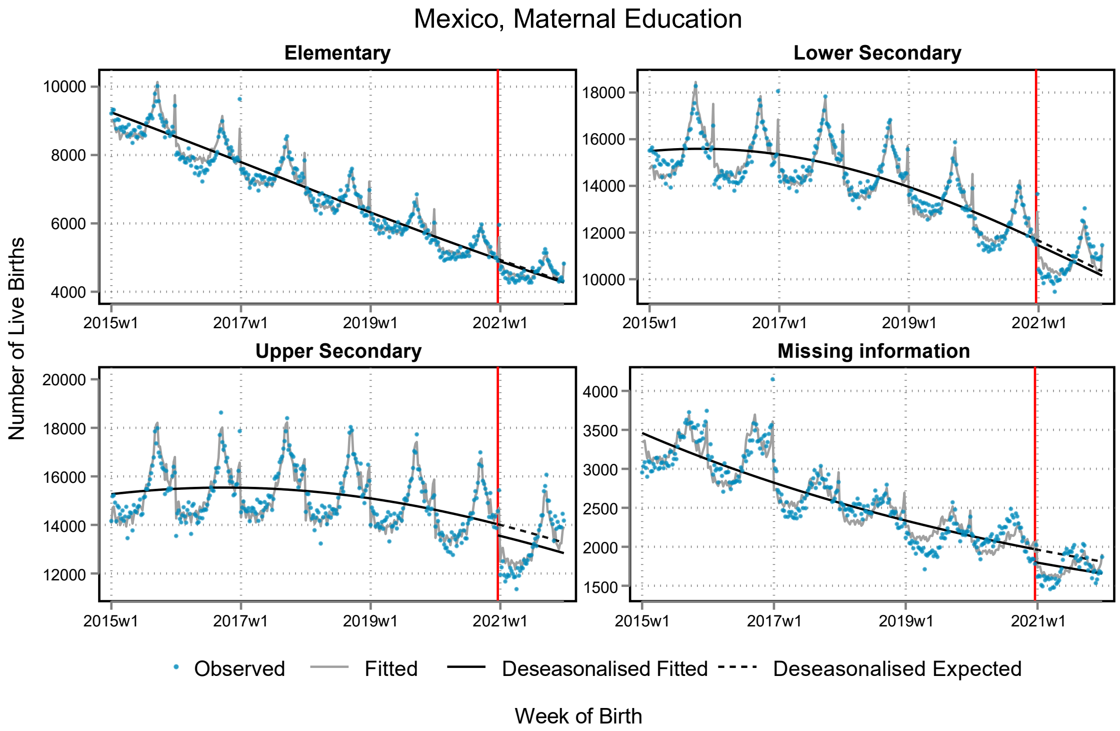

*Supplementary Figure 37: Observed and expected weekly number of live births in **Mexico** by primary indicator of socioeconomic circumstances (maternal education). Expected numbers are estimated by subgroup-specific Poisson regression modes on the full time series including an indicator variable for the exposed period (starting second week of December of 2020 to December 2021) to estimate the average effect of the COVID-19 pandemic over the entire period; a linear and a quadratic term for week of live birth to capture potential non-linearities in the secular time trends; week of the year fixed effects to account for seasonality; an indicator variable (August 2016-December 2016) to account for the 2015-2016 Zika virus epidemic.*

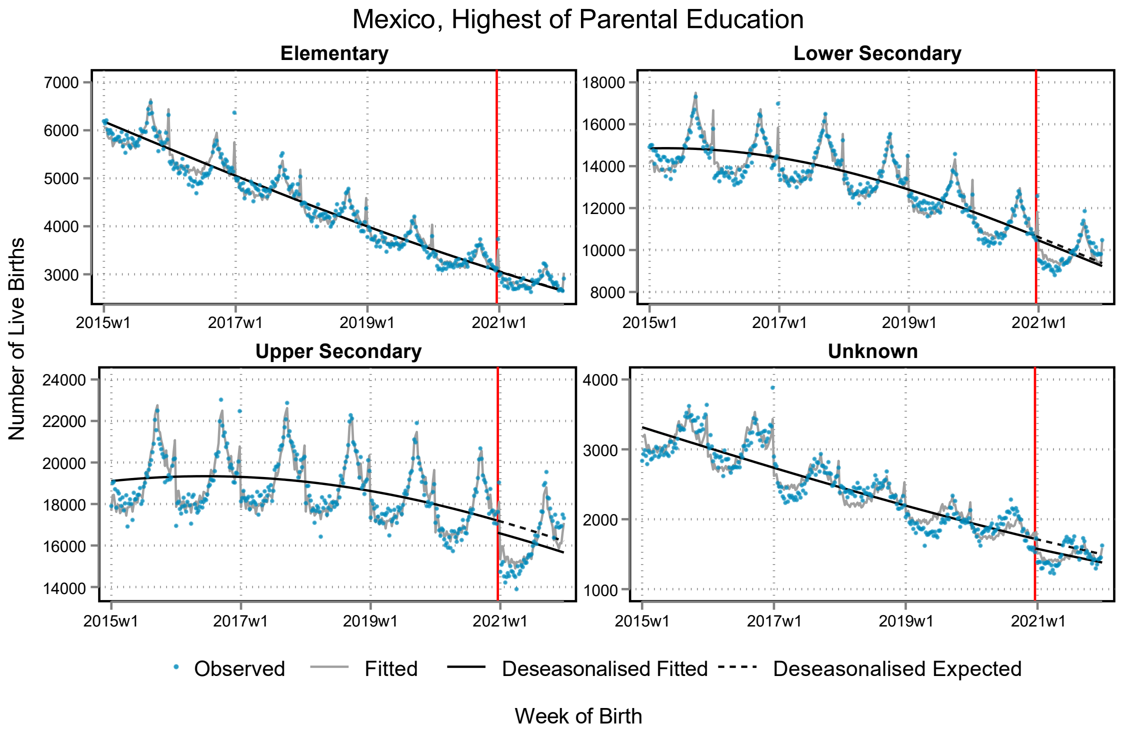

*Supplementary Figure 38: Observed and expected weekly number of live births in **Mexico** paternal education. Expected numbers are estimated by subgroup-specific Poisson regression modes on the full time series including an indicator variable for the exposed period (starting second week of December 2020 to December 2021) to estimate the average effect of the COVID-19 pandemic over the entire period; a linear and a quadratic term for week of live birth to capture potential non-linearities in the secular time trends; week of the year fixed effects to account for seasonality; an indicator variable (August 2016-December 2016) to account for the 2015-2016 Zika virus epidemic.*

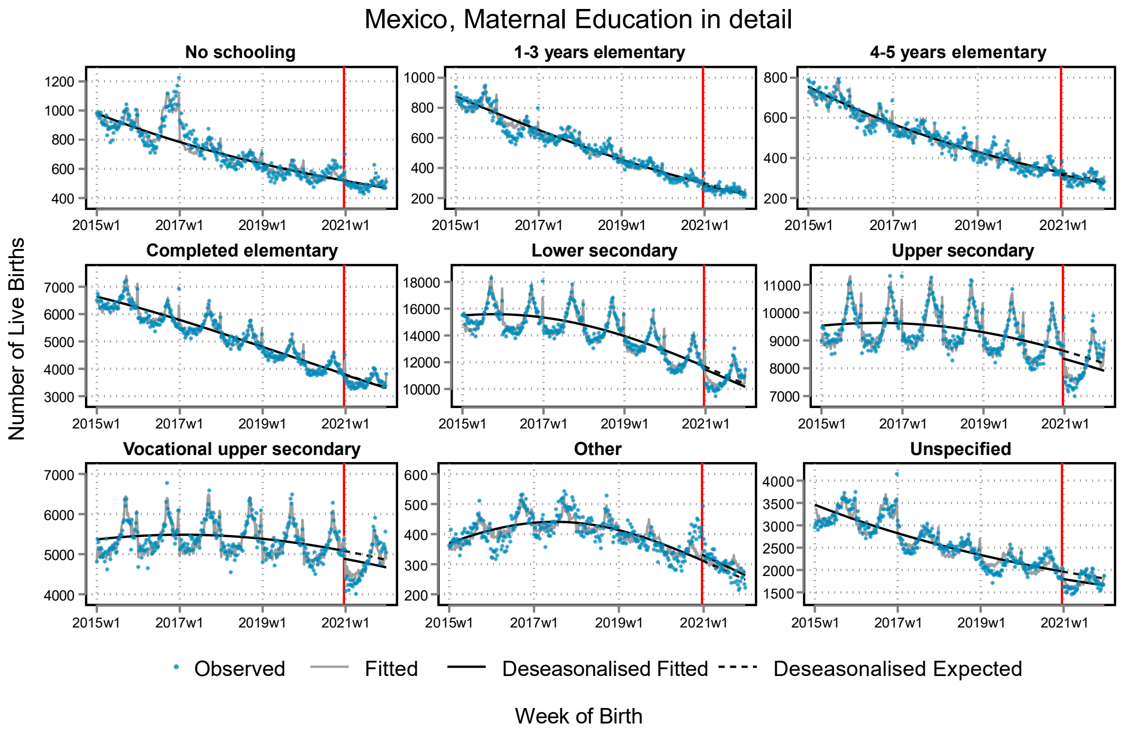

Supplementary Figure 39: Observed and expected weekly number of live births in ***Mexico*** *by maternal education in detail. Expected numbers are estimated by subgroup-specific Poisson regression modes on the full time series including an indicator variable for the exposed period (starting second week of December 2020 to December 2021) to estimate the average effect of the COVID-19 pandemic over the entire period; a linear and a quadratic term for week of live birth to capture potential non-linearities in the secular time trends; week of the year fixed effects to account for seasonality; an indicator variable (August 2016-December 2016) to account for the 2015-2016 Zika virus epidemic.*

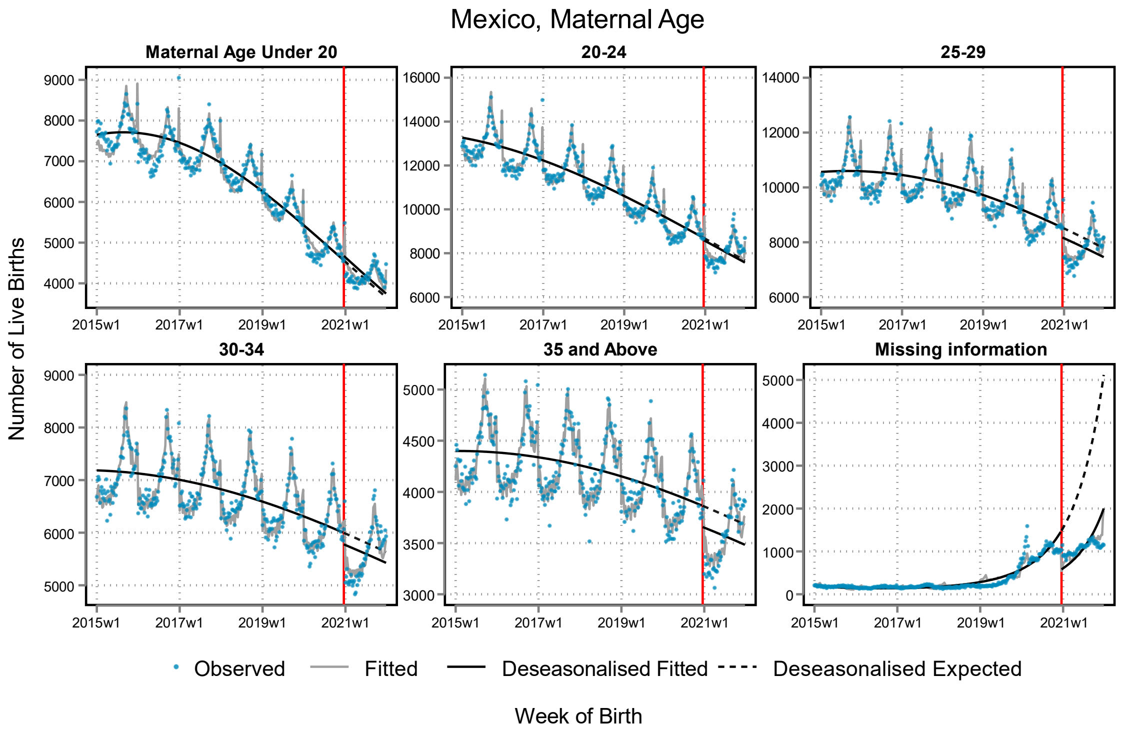

*Supplementary Figure 40: Observed and expected weekly number of live births in **Mexico** maternal age. Expected numbers are estimated by subgroup-specific Poisson regression modes on the full time series including an indicator variable for the exposed period (starting second week of December 2020 to December 2021) to estimate the average effect of the COVID-19 pandemic over the entire period; a linear and a quadratic term for week of live birth to capture potential non-linearities in the secular time trends; week of the year fixed effects to account for seasonality; an indicator variable (August 2016-December 2016) to account for the 2015-2016 Zika virus epidemic.*

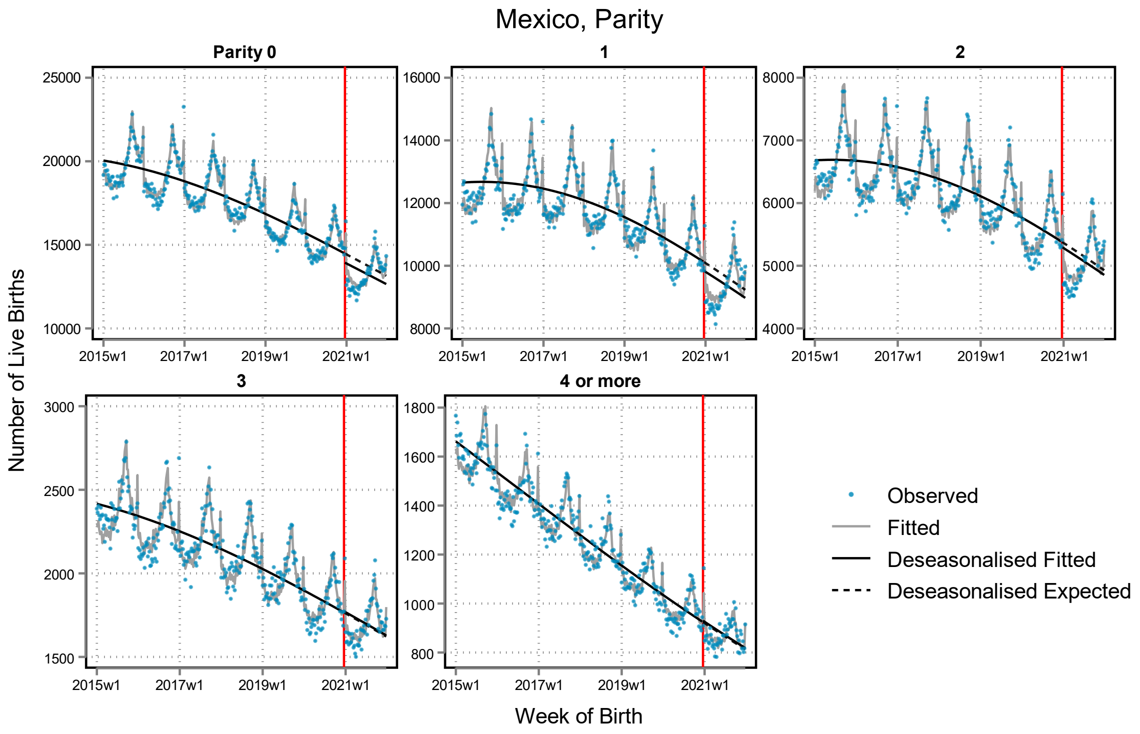

*Supplementary Figure 41: Observed and expected weekly number of live births in **Mexico** by parity. Expected numbers are estimated by subgroup-specific Poisson regression modes on the full time series including an indicator variable for the exposed period (starting second week of December 2020 to December 2021) to estimate the average effect of the COVID-19 pandemic over the entire period; a linear and a quadratic term for week of live birth to capture potential non-linearities in the secular time trends; week of the year fixed effects to account for seasonality; an indicator variable (August 2016-December 2016) to account for the 2015-2016 Zika virus epidemic.*

Supplementary Table 9: Relative and Percentage Point Differences in the Composition of the December 2020 – December 2021 Birth Cohort in Mexico. "Observed" is abbreviated by "OBS" and "Counterfactual" is abbreviated by "CF". Statistical methods for the estimations are described in the main manuscript.

Characteristic	Observed (OBS) Births	Counterfactual (CF) Births	95%CI: CF Births	OBS - CF Births	95%CI: OBS - CF Births	% more/less than CF	95%CI: % more/less than CF	OBS proportion	CF proportion	OBS - CF proportion	95%CI: OBS - CF proportion
**Highest Parental Education**											
Elementary	154527	154932	(153738; 156126)	-405	(-1826; 1016)	-0.3	(-1.0; 0.5)	9.4	9.2	0.2	(0.2; 0.3)
Lower Secondary	533804	541742	(539427; 544057)	-7938	(-10660; -5216)	-1.5	(-1.9; -1.0)	32.5	32.1	0.4	(0.3; 0.5)
Upper Secondary	873804	904577	(901428; 907725)	-30773	(-34416; -27130)	-3.4	(-3.7; -3.1)	53.2	53.6	-0.4	(-0.5; -0.3)
Unknown	80189	87058	(86153; 87963)	-6869	(-7931; -5807)	-7.9	(-8.8; -6.9)	4.9	5.2	-0.3	(-0.3; -0.2)
**total**	1642324	1688308		-45984		0.0		100.0	100.0	0.0	
**Maternal education detailed**											
No Schooling	26740	26796	(26285; 27306)	-56	(-658; 547)	-0.2	(-2.1; 1.7)	1.6	1.6	0.0	(0.0; 0.1)
1-3 years elementary	13861	14458	(14118; 14797)	-597	(-1007; -186)	-4.1	(-6.3; -1.8)	0.8	0.9	0.0	(-0.0; 0.0)
4-5 years elementary	15942	16489	(16102; 16876)	-547	(-1006; -87)	-3.3	(-5.5; -1.0)	1.0	1.0	0.0	(-0.0; 0.0)
Completed Elementary	191538	192415	(191065; 193766)	-877	(-2477; 723)	-0.5	(-1.1; 0.2)	11.7	11.4	0.3	(0.2; 0.3)
Lower Secondary	585510	596123	(593680; 598567)	-10613	(-13480; -7746)	-1.8	(-2.2; -1.4)	35.7	35.3	0.3	(0.2; 0.5)
Upper Secondary	440252	455235	(452995; 457475)	-14983	(-17573; -12393)	-3.3	(-3.8; -2.8)	26.8	27.0	-0.2	(-0.3; -0.0)
Vocational Upper Secondary	258798	269171	(267435; 270907)	-10373	(-12375; -8371)	-3.9	(-4.5; -3.2)	15.8	15.9	-0.2	(-0.3; -0.1)
Other	16048	15143	(14772; 15515)	905	(458; 1351)	6.0	(3.4; 8.6)	1.0	0.9	0.1	(0.1; 0.1)
Unknown	93635	102284	(101270; 103298)	-8649	(-9828; -7471)	-8.5	(-9.4; -7.5)	5.7	6.1	-0.4	(-0.4; -0.3)
**total**	1642324	1688114		-45790		0.0		100.0	100.0	0.0	
**Maternal age**											
Below 20	227362	222072	(220675; 223469)	5290	(3609; 6971)	2.4	(1.7; 3.0)	13.8	12.5	1.3	(1.2; 1.4)
20-24	437267	442513	(440431; 444595)	-5246	(-7698; -2794)	-1.2	(-1.6; -0.7)	26.6	24.9	1.7	(1.6; 1.8)

25-29	422982	441556	(439407; 443705)	-18574	(-21073; -16076)	-4.2	(-4.7; -3.7)	25.8	24.9	0.9	(0.8; 1.0)
30-34	303145	314672	(312828; 316516)	-11527	(-13664; -9390)	-3.7	(-4.2; -3.1)	18.5	17.7	0.7	(0.6; 0.8)
Above 34	193310	204263	(202761; 205765)	-10953	(-12685; -9221)	-5.4	(-6.1; -4.7)	11.8	11.5	0.3	(0.2; 0.3)
Unknown	58258	148963	(146020; 151906)	-90705	(-93685; -87724)	-60.9	(-61.6; -60.1)	3.5	8.4	-4.8	(-5.0; -4.7)
**total**	1642324	1774040		-131716		-0.1		100.0	100.0	0.0	
**Parity**											
0	719624	747844	(745077; 750611)	-28220	(-31448; -24991)	-3.8	(-4.1; -3.4)	43.8	44.3	-0.5	(-0.6; -0.3)
1	508966	523526	(521189; 525863)	-14560	(-17283; -11836)	-2.8	(-3.2; -2.3)	31.0	31.0	0.0	(-0.1; 0.1)
2	274552	278815	(277106; 280524)	-4263	(-6257; -2270)	-1.5	(-2.1; -0.9)	16.7	16.5	0.2	(0.1; 0.3)
3	91955	91585	(90612; 92559)	370	(-771; 1510)	0.4	(-0.7; 1.5)	5.6	5.4	0.2	(0.1; 0.2)
4 or more	47227	46937	(46268; 47606)	290	(-503; 1083)	0.6	(-0.8; 2.1)	2.9	2.8	0.1	(0.1; 0.1)
**total**	1642324	1688707		-46383		0.0		100.0	100.0	0.0	

Netherlands

Data

For the Netherlands (2015-2021, n=1,198,205 live births), we used restricted access individual-level data from the live births registry dataset, which was linked to parental characteristics and household income records through Statistics Netherlands (CBS – Bureau voor de Statistiek) population records (project number 8552). We aggregated the data to monthly time series by household income quintile based on the population-wide equivalised household income distribution. Data on household income was drawn from the annual income of households dataset. This dataset contains information on household disposable income adjusted for household size and composition. To calculate household incomes for each reporting year, tax records and other income information up to January 1 of that year are used. For example, household incomes for the year 2020 are based on tax records and other income information up to January 1 of the year 2020. Thus, household income in 2020 cannot be affected by pandemic effects income. Household incomes of 2021, however, are affected by potential pandemic effects on household incomes. Household income was not lagged by 2 years (instead of 1) to obtain pre-pandemic household income (as done for Finland) due to a misunderstanding between collaborators. This means that the observed compositional change could be an artifact of pandemic-induced changes of the income distribution. This, however, would require a high number of households changing their pre-pandemic fifth in the household income distribution. As the results are consistent with those of other countries, we believe that the potential bias caused by this non-lagged measure of household income quintile is limited.

Apart from compositional changes in the household income composition, we analysed compositional changes regarding maternal age and between first born and higher order births.

Results

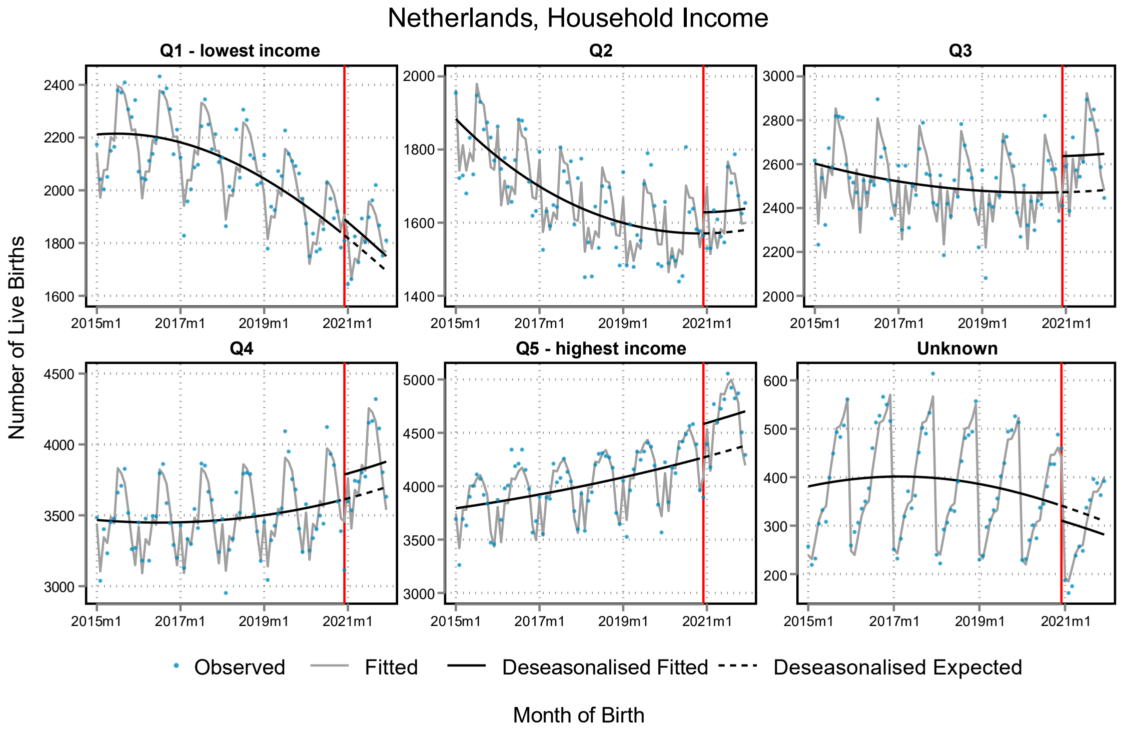

*Supplementary Figure 42: Observed and expected monthly number of live births in **the Netherlands** by quintile of equivalised disposable household (primary socioeconomic indicator). Expected numbers are estimated by subgroup-specific Poisson regression modes on the full time series including an indicator variable for the exposed period (December 2020 to December 2021) to estimate the average effect of the COVID-19 pandemic over the entire period; a linear and a quadratic term for month of live birth to capture potential non-linearities in the secular time trends; month of the year fixed effects to account for seasonality.*

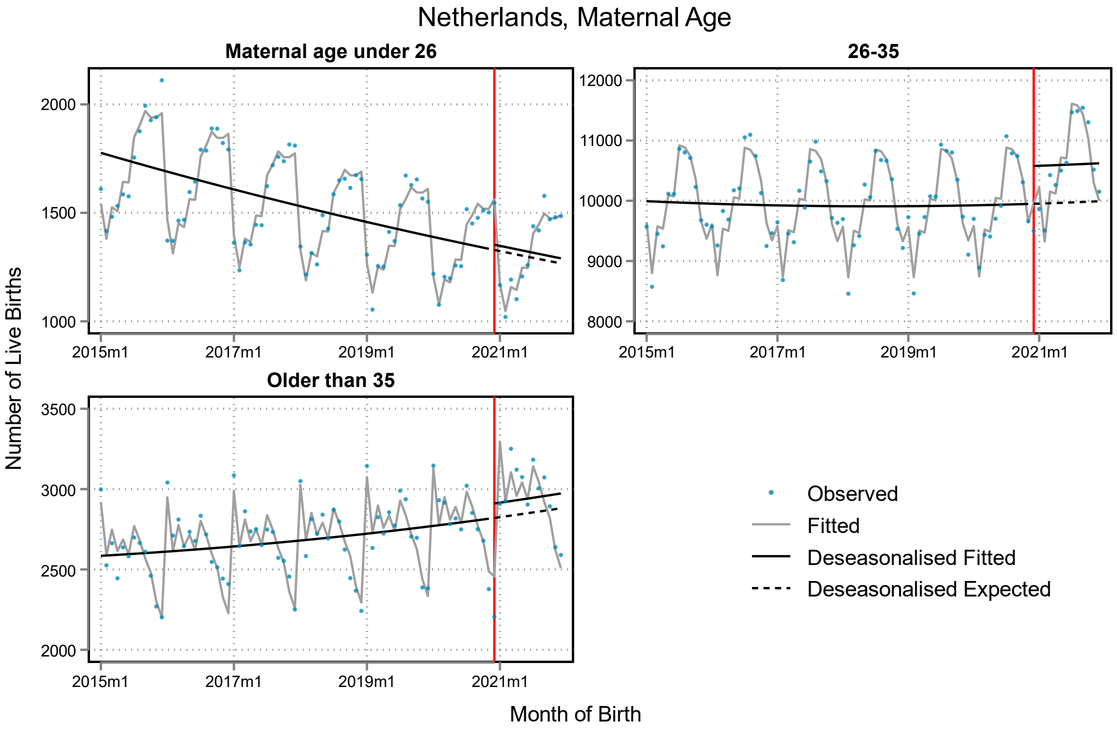

*Supplementary Figure 43: Observed and expected monthly number of live births in **the Netherlands** by maternal age. Expected numbers are estimated by subgroup-specific Poisson regression modes on the full time series including an indicator variable for the exposed period (starting December 2020 to December 2021) to estimate the average effect of the COVID-19 pandemic over the entire period; a linear and a quadratic term for month of live birth to capture potential non-linearities in the secular time trends; month of the year fixed effects to account for seasonality.*

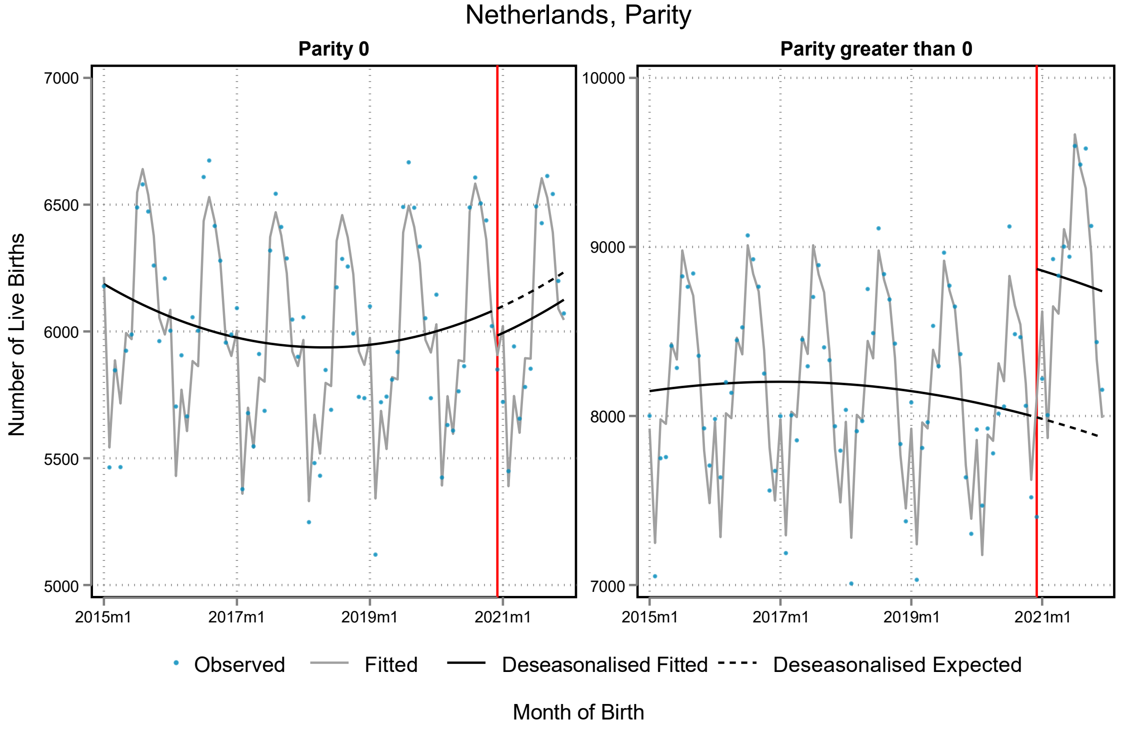

*Supplementary Figure 44: Observed and expected monthly number of live births in **the Netherlands** by parity. Expected numbers are estimated by subgroup-specific Poisson regression modes on the full time series including an indicator variable for the exposed period (starting December 2020 to December 2021) to estimate the average effect of the COVID-19 pandemic over the entire period; a linear and a quadratic term for month of live birth to capture potential non-linearities in the secular time trends; month of the year fixed effects to account for seasonality.*

Supplementary Table 10: Relative and Percentage Point Differences in the Composition of the December 2020 – December 2021 Birth Cohort in the Netherlands. "Observed" is abbreviated by "OBS" and "Counterfactual" is abbreviated by "CF". Statistical methods for the estimations are described in the main manuscript.

Characteristic	Observed (OBS) Births	Counterfactual (CF) Births	95%CI: CF Births	OBS - CF Births	95%CI: OBS - CF Births	% more/less than CF	95%CI: % more/less than CF	OBS proportion	CF proportion	OBS - CF proportion	95%CI: OBS - CF proportion
**Maternal age**											
Below 26	17363	17049	(16613; 17486)	314	(-193; 821)	1.8	(-0.7; 4.5)	9.0	9.3	-0.3	(-0.5; -0.1)
26-35	137174	129036	(127792; 130279)	8138	(6699; 9578)	6.3	(5.3; 7.3)	71.3	70.6	0.7	(0.3; 1.1)
Older than 35	37772	36603	(35933; 37272)	1170	(399; 1940)	3.2	(1.3; 5.1)	19.6	20.0	-0.4	(-0.7; -0.1)
**total**	192309	182687		9622		0.1		100.0	100.0	0.0	
**Parity**											
Firstborn	78597	79988	(78996; 80980)	-1391	(-2525; -257)	-1.7	(-2.9; -0.5)	40.9	43.8	-3.0	(-3.4; -2.6)
Second or higher birth order	113712	102462	(101369; 103555)	11250	(9972; 12528)	11.0	(9.8; 12.2)	59.1	56.2	3.0	(2.6; 3.4)
**total**	192309	182450		9859		0.1		100.0	100.0	0.0	

Scotland

Data

For Scotland (2015-2021, n=344,134 live births), we used openly available aggregated monthly time series from the Scottish Morbidity Record 02 (SMR02) by quintiles of the Scottish Index of Multiple Deprivation 2020 (SIMD) and maternal age categories. (https://scotland.shinyapps.io/phs-covid-wider-impact/) The SMR02 covers around 99% of births registered with the National Records of Scotland.

The SIMD combines 33 indicators from 7 domains (income, employment, health, education, geographic access to services, crime, and Housing) and is measured on the data zone level. (See here for technical notes:

https://www.gov.scot/binaries/content/documents/govscot/publications/statistics/2020/09/simd-2020-technical-notes/documents/simd-2020-technical-notes/simd-2020-technical-notes/govscot%3Adocument/SIMD%2B2020%2Btechnical%2Bnotes.pdf)

There are 6,976 data zones in Scotland with an average population of 784 people. Births are allocated to a quintile of the SIMD based on their mother's residential data zone. The SIMD is created such that each quintile contains a fifth of the overall population of Scotland.

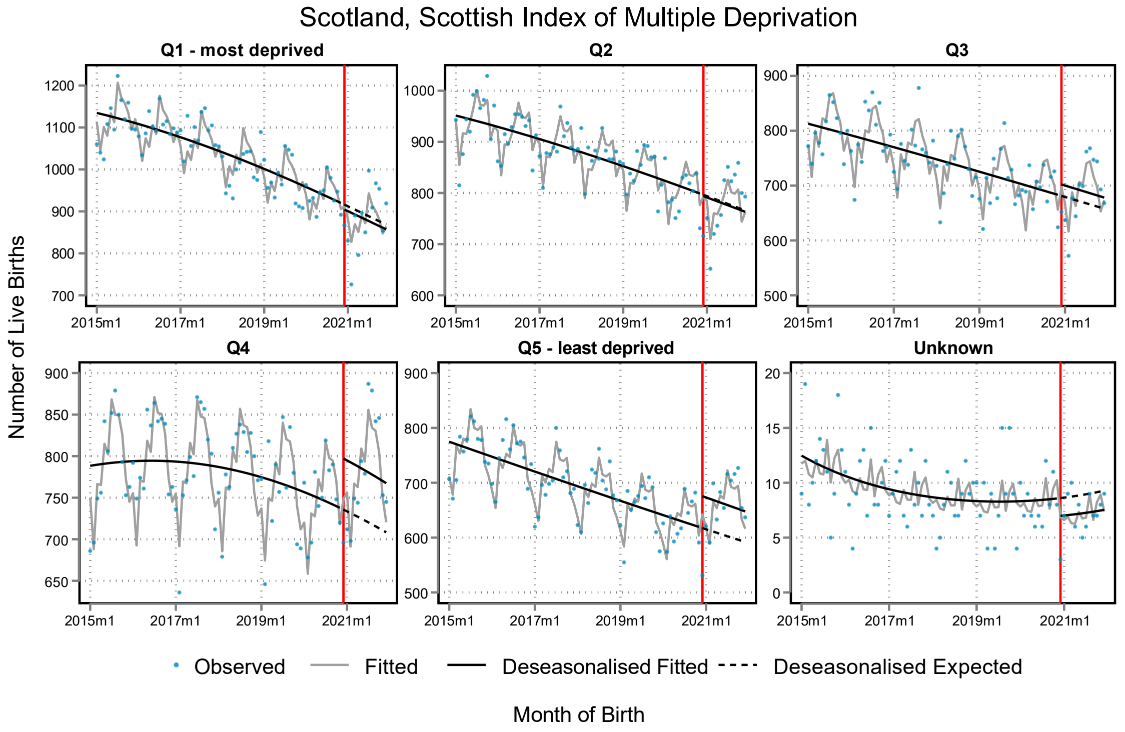

*Supplementary Figure 45: Observed and expected monthly number of live births in **Scotland** by quintile of Scottish Index of Multiple Deprivation of maternal residential location (primary socioeconomic indicator). Expected numbers are estimated by subgroup-specific Poisson regression modes on the full time series including an indicator variable for the exposed period (starting December 2020 to December 2021) to estimate the average effect of the COVID-19 pandemic over the entire period; a linear and a quadratic term for month of live birth to capture potential non-linearities in the secular time trends; month of the year fixed effects to account for seasonality.*

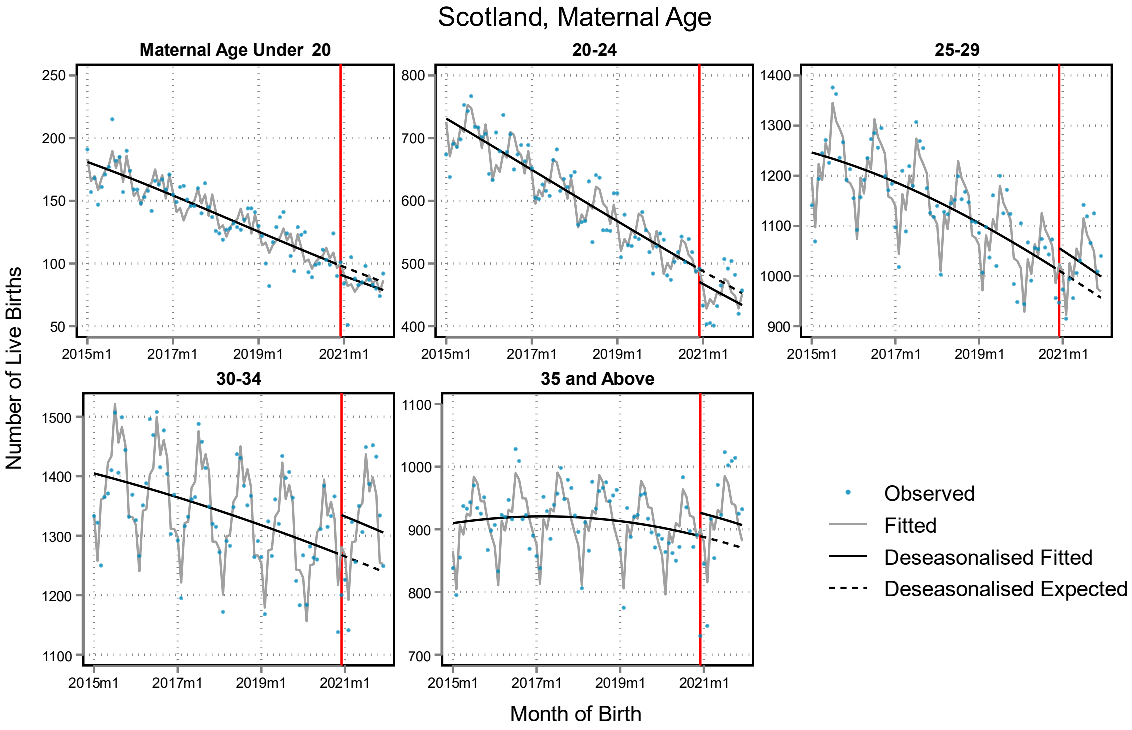

*Supplementary Figure 46: Observed and expected monthly number of live births in **Scotland** by maternal age. Expected numbers are estimated by subgroup-specific Poisson regression modes on the full time series including an indicator variable for the exposed period (starting December 2020 to December 2021) to estimate the average effect of the COVID-19 pandemic over the entire period; a linear and a quadratic term for month of live birth to capture potential non-linearities in the secular time trends; month of the year fixed effects to account for seasonality.*

Supplementary Table 11: Relative and Percentage Point Differences in the Composition of the December 2020 – December 2021 Birth Cohort in Scotland. "Observed" is abbreviated by "OBS" and "Counterfactual" is abbreviated by "CF". Statistical methods for the estimations are described in the main manuscript.

Characteristic	Observed (OBS) Births	Counterfactual (CF) Births	95%CI: CF Births	OBS - CF Births	95%CI: OBS - CF Births	% more/less than CF	95%CI: % more/less than CF	OBS proportion	CF proportion	OBS - CF proportion	95%CI: OBS - CF proportion
**Maternal age**											
Below 20	1111	1200	(1092; 1308)	-89	(-215; 37)	-7.4	(-15.1; 1.7)	2.3	2.5	-0.3	(-0.5; -0.0)
20-24	5893	6150	(5895; 6405)	-257	(-553; 39)	-4.2	(-8.0; 0.0)	11.9	12.9	-0.9	(-1.4; -0.4)
25-29	13323	12756	(12381; 13131)	567	(129; 1005)	4.4	(1.5; 7.6)	27.0	26.7	0.3	(-0.4; 1.0)
30-34	17106	16244	(15811; 16676)	862	(359; 1366)	5.3	(2.6; 8.2)	34.7	34.0	0.7	(-0.1; 1.4)
Above 34	11891	11414	(11048; 11779)	477	(54; 901)	4.2	(0.9; 7.6)	24.1	23.9	0.2	(-0.5; 0.9)
**total**	49324	47764		1560		0.0		100.0	100.0	0.0	

South Australia

Data

For South Australia (2015-2021, n=133,337 live births), we used restricted access individual-level data from the Better Evidence Better Outcomes Linked Data (BEBOLD) platform. BEBOLD includes births registered in South Australia for all children born 1991 to 2022 and their parents. It includes all births ≤20 weeks or more than 400 grams (alive or stillborn), which are legally required to be registered within 60 days of birth. For more information about the BEBOLD platform, see (https://health.adelaide.edu.au/betterstart/bebold). The data used for this study were provided by South Australian State government agencies under agreements with the researchers led by JL, and SANT DataLink, following approval from multiple ethics committees and data custodians. No data are available because Australian privacy laws prohibit us from making individual-level de-identified data publicly available. Data are only able to be accessed by researchers who are approved users by the relevant ethics committees and data custodians. Data can be accessed through an application and approval process administered by the independent data linkage authority, SANT DataLink. This study was approved by the SA Department for Health and Wellbeing Human Research Ethics Committee (2022/HRE00137) and The University of Adelaide Human Research Ethics Committee (37934). We thank SANT Datalink for managing and conducting the data linkage for the BEBOLD (Better Evidence, Better Outcomes, Linked Data) data platform (led by JL). The findings and views reported in this study are those of the authors and should not be attributed to any agency or government department.

As an indicator for socioeconomic circumstances, we used the Index of Relative Socio-economic Advantage and Disadvantage (IRSAD) measured with 2016 census data. The IRSAD 2016 includes several area-level indicators of advantage and disadvantage, like household income, educational level, occupation, unemployment level, household overcrowding. Mother's postal code at the time of birth was assigned an IRSAD quintile.
For a detailed description of the IRSAD 2016, please see
https://www.abs.gov.au/methodologies/socio-economic-indexes-areas-seifa-australia-methodology/2021#index-of-relative-socio-economic-advantage-and-disadvantage-irsad-.

Although IRSAD measures exists with 2021 census data, we applied the 2016 IRSAD data to all births to avoid inconsistencies in data collection which led to sudden jumps in the quintile-specific number of births because areas changes IRSAD quintiles over time.

We also estimate compositional changes along maternal age.

Results

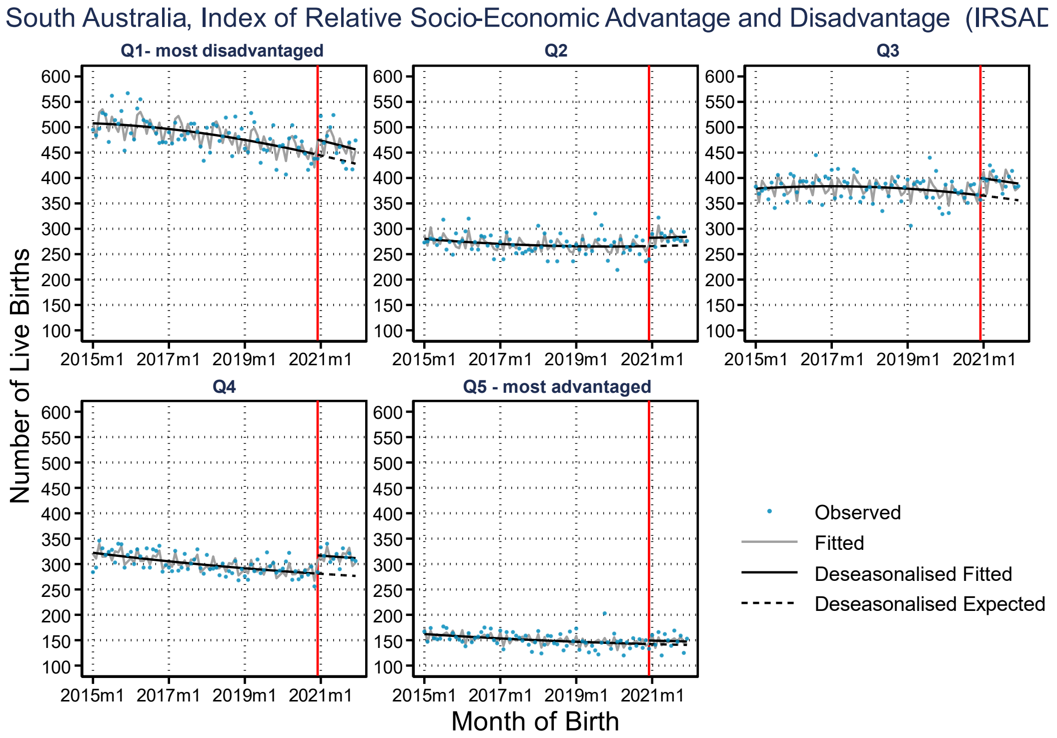

*Supplementary Figure 47: Observed and expected monthly number of live births in **South Australia** by quintile of Index of Relative Socio-economic Advantage and Disadvantage (IRSAD) of maternal residential location (primary socioeconomic indicator). Expected numbers are estimated by subgroup-specific Poisson regression modes on the full time series including an indicator variable for the exposed period (starting December 2020 to December 2021) to estimate the average effect of the COVID-19 pandemic over the entire period; a linear and a quadratic term for month of live birth to capture potential non-linearities in the secular time trends; month of the year fixed effects to account for seasonality.*

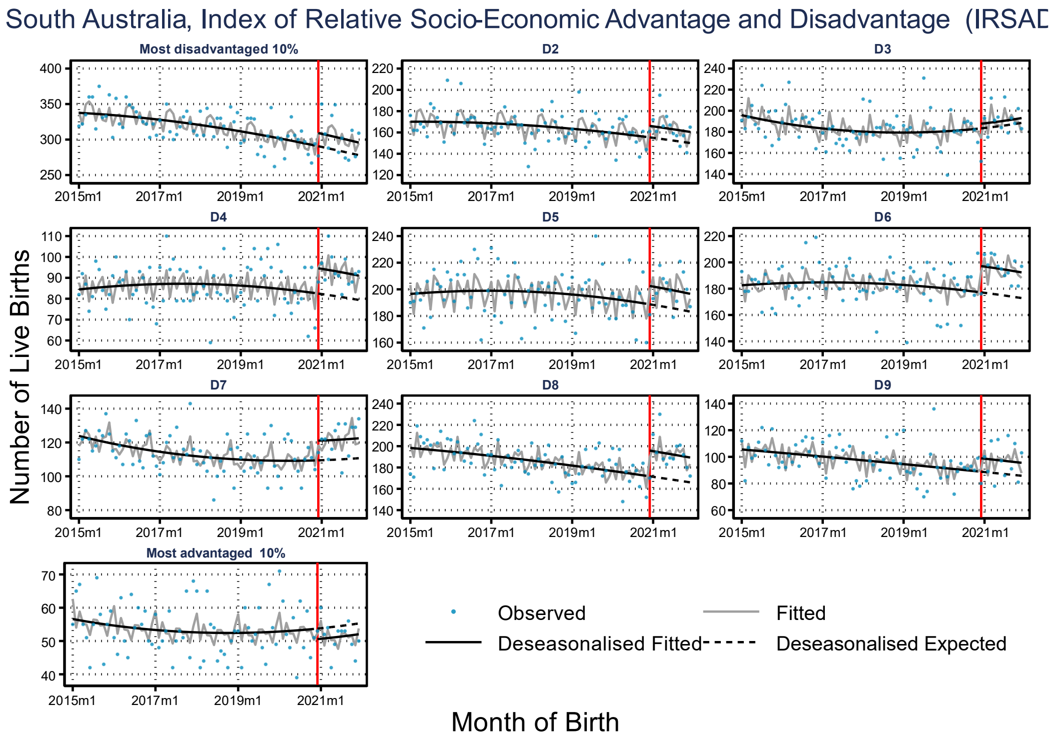

*Supplementary Figure 48: Observed and expected monthly number of live births in **South Australia** by decile of Index of Relative Socio-economic Advantage and Disadvantage (IRSAD) of maternal residential location (secondary socioeconomic indicator). Expected numbers are estimated by subgroup-specific Poisson regression modes on the full time series including an indicator variable for the exposed period (starting December 2020 to December 2021) to estimate the average effect of the COVID-19 pandemic over the entire period; a linear and a quadratic term for month of live birth to capture potential non-linearities in the secular time trends; month of the year fixed effects to account for seasonality.*

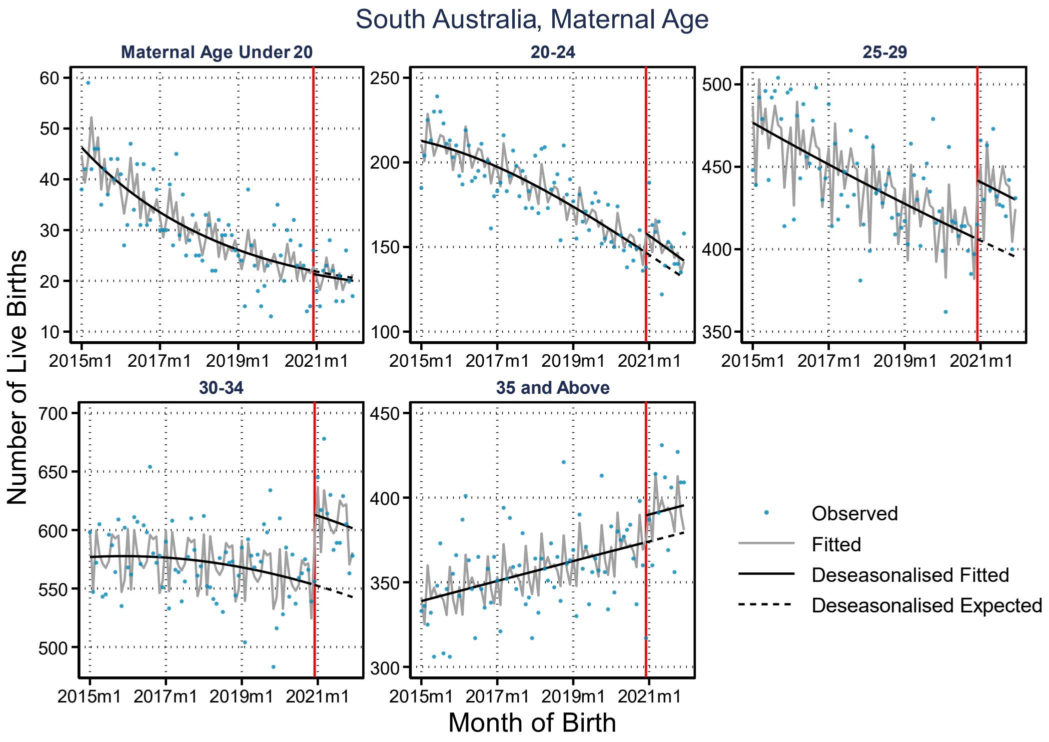

Supplementary Figure 49: Observed and expected monthly number of live births in ***South Australia*** *by maternal age. Expected numbers are estimated by subgroup-specific Poisson regression modes on the full time series including an indicator variable for the exposed period (starting December 2020 to December 2021) to estimate the average effect of the COVID-19 pandemic over the entire period; a linear and a quadratic term for month of live birth to capture potential non-linearities in the secular time trends; month of the year fixed effects to account for seasonality.*

Supplementary Table 12: Relative and Percentage Point Differences in the Composition of the December 2020 – December 2021 Birth Cohort in South Australia. "Observed" is abbreviated by "OBS" and "Counterfactual" is abbreviated by "CF". Statistical methods for the estimations are described in the main manuscript.

Characteristic	Observed (OBS) Births	Counterfactual (CF) Births	95%CI: CF Births	OBS - CF Births	95%CI: OBS - CF Births	% more/less than CF	95%CI: % more/less than CF	OBS proportion	CF proportion	OBS - CF proportion	95%CI: OBS - CF proportion
**Index of Multiple Deprivation**											
D1 - Most Deprived	3933	3697	(3493; 3901)	236	(-2; 474)	6.4	(0.8; 12.6)	18.9	19.2	-0.3	(-1.3; 0.7)
D2	2123	1985	(1834; 2136)	138	(-38; 314)	6.9	(-0.6; 15.7)	10.2	10.3	-0.1	(-0.9; 0.6)
D3	2459	2401	(2230; 2572)	58	(-138; 254)	2.4	(-4.4; 10.3)	11.8	12.5	-0.7	(-1.5; 0.2)
D4	1208	1053	(944; 1163)	155	(25; 284)	14.7	(3.8; 28.0)	5.8	5.5	0.3	(-0.2; 0.9)
D5	2592	2416	(2249; 2583)	176	(-18; 371)	7.3	(0.4; 15.3)	12.4	12.5	-0.1	(-0.9; 0.7)
D6	2529	2272	(2110; 2435)	257	(67; 446)	11.3	(3.9; 19.8)	12.1	11.8	0.3	(-0.4; 1.1)
D7	1579	1427	(1297; 1557)	152	(0; 303)	10.6	(1.4; 21.7)	7.6	7.4	0.2	(-0.5; 0.8)
D8	2498	2190	(2033; 2347)	308	(123; 493)	14.0	(6.4; 22.9)	12.0	11.4	0.6	(-0.2; 1.4)
D9	1256	1128	(1016; 1240)	128	(-4; 260)	11.3	(1.3; 23.6)	6.0	5.9	0.2	(-0.4; 0.8)
D10 - Least Deprived	668	710	(616; 803)	-42	(-148; 65)	-5.9	(-16.8; 8.5)	3.2	3.7	-0.5	(-1.0; -0.0)
**total**	20845	19280		1565		8.1		100.0	100.0	0.0	
**Maternal age**											
Below 20	270	278	(224; 331)	-8	(-70; 55)	-2.8	(-18.4; 20.4)	1.3	1.4	-0.1	(-0.4; 0.1)
20-24	1950	1809	(1673; 1945)	141	(-20; 302)	7.8	(0.3; 16.5)	9.4	9.4	0.0	(-0.7; 0.7)
25-29	5663	5204	(4961; 5447)	459	(174; 743)	8.8	(4.0; 14.2)	27.2	27.0	0.2	(-0.9; 1.3)
30-34	7877	7106	(6819; 7393)	771	(435; 1107)	10.8	(6.5; 15.5)	37.8	36.9	0.9	(-0.3; 2.1)
Above 34	5088	4881	(4636; 5126)	207	(-75; 489)	4.2	(-0.7; 9.8)	24.4	25.3	-0.9	(-2.0; 0.2)
total	20848	19278		1570		8.1		100.0	100.0	0.0	

Spain

Data

For Spain (2015-2021; n=2,636,143 live births), we used the openly available individual-level birth register data (collected from birth certificates) provided by the Instituto Nacional de Estadística (INE): (https://www.ine.es/dyngs/INEbase/en/operacion.htm?c=Estadistica_C&cid=1254736177007&menu=resultados&secc=1254736195443&idp=1254735573002#!tabs-1254736195443). We aggregated the data to monthly time series by maternal education, highest parental education, age, and parity. This data source covers the whole "universe" of births in Spain.

For the analysis of the socioeconomic composition of birth cohorts, we use data from 2016-2021 as data collection on parental educational changed for vital statistics from 2016 onwards. Since 2016, data on parental education is drawn from 11 different register-based data sources to improve data quality. They collect 12 different educational levels: 1) Illiterates, 2) Incomplete primary education, 3) Primary Education, 4) First stage of secondary education, 5) Second stage of secondary education with general orientation, 6) Second stage of secondary education with professional orientation, 7) Non-higher post-secondary education, 8) Vocational training, visual arts and design, and higher-level sports education and the equivalent; university degrees requiring a baccalaureate diploma, lasting two years or more, 9) University degrees of 240 ECTS credits, university graduates, own university expert or specialist qualifications and equivalent, 10) University degrees of over 240 ECTS credits, bachelor's degrees, 11) Specialities in Health Sciences for the residence system and similar, and 12) University PhD. (See page 22-24 in the documentation: https://www.ine.es/en/metodologia/t20/t2030301_en.pdf)

We assigned the CNED-A (National Classification of Education) levels 1,2,3,4 to lower secondary education or lower ("No Highschool Diploma" in Figure S41, S42); level 5,6 to upper secondary education; level 7 to 12 to post-secondary and tertiary education.

Additionally, we estimated compositional change regarding highest parental education (if maternal education was missing, we used paternal education), maternal age, and parity.

In the Spanish Vital Statistics, the educational level is only assigned to people over 25 years of age. Thus, our analysis of changes in socioeconomic composition only refers to mother aged 26 or older. Further, assignment of educational levels changed in 2018 causing an abrupt change in the level (not seasonality or trend) of the number of births between 2016-2017, and 2018-2021. To account for this jump in the time series, we included a binary variable indicating the years 2016 and 2017 in our models estimating level changes in the number of births by parental education.

For analyses regarding maternal age and parity, we used data covering 2015-2021 in line with analyses for other countries.

Results

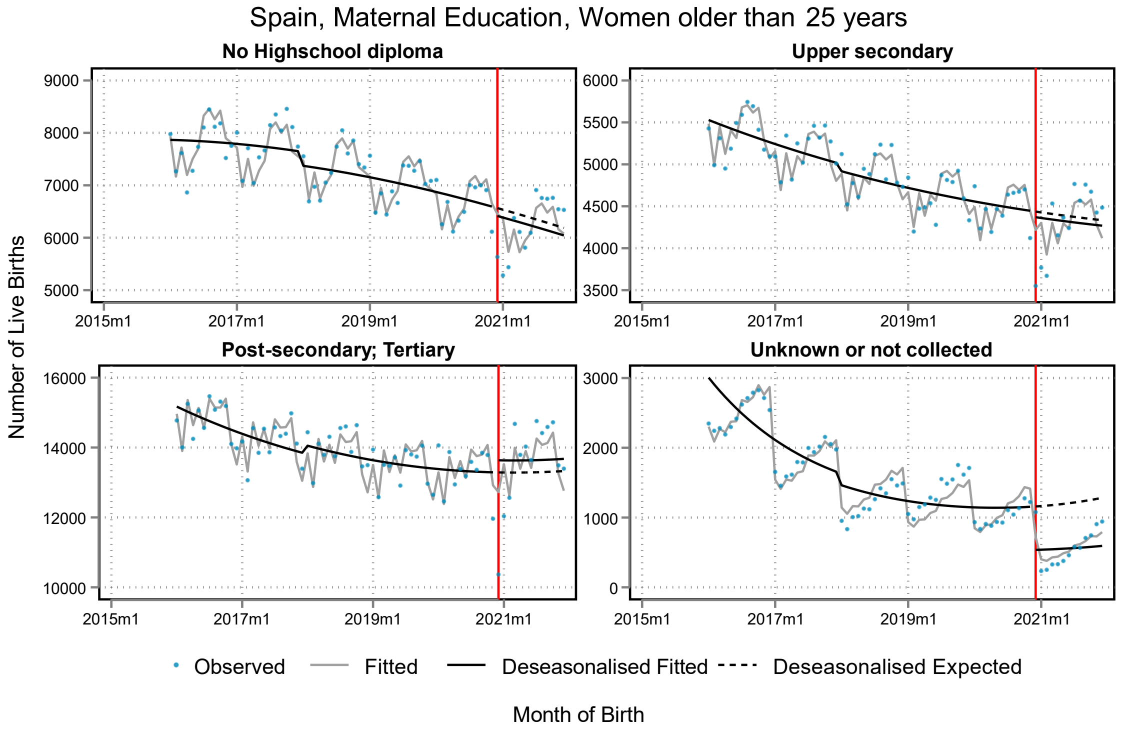

*Supplementary Figure 50: Observed and expected monthly number of live births in **Spain** by maternal education (primary socioeconomic indicator). Expected numbers are estimated by subgroup-specific Poisson regression modes on the full time series including an indicator variable for the exposed period (starting December 2020 to December 2021) to estimate the average effect of the COVID-19 pandemic over the entire period; a linear and a quadratic term for month of live birth to capture potential non-linearities in the secular time trends; month of the year fixed effects to account for seasonality; an indicator variable for 2016 and 2017 to capture changes in data collection on maternal education. Data is restricted to women older than 25 due to restricted data quality on maternal education below 26 years of age. The time series starts in 2016 because data collection on maternal education changed in 2016.*

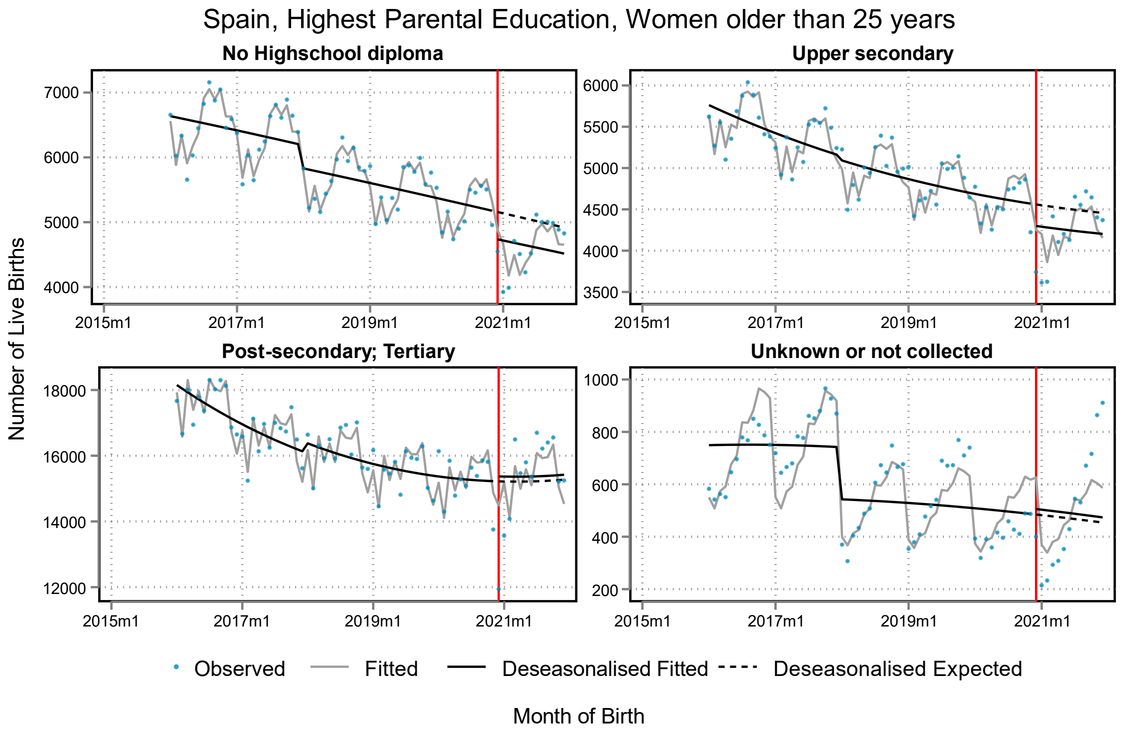

Supplementary Figure 51: Observed and expected monthly number of live births in ***Spain*** *by highest parental education. We used father's education if maternal education was not available. Expected numbers are estimated by subgroup-specific Poisson regression modes on the full time series including an indicator variable for the exposed period (starting December 2020 to December 2021) to estimate the average effect of the COVID-19 pandemic over the entire period; a linear and a quadratic term for month of live birth to capture potential non-linearities in the secular time trends; month of the year fixed effects to account for seasonality; an indicator variable for 2016 and 2017 to capture changes in data collection on maternal education. Data is restricted to women older than 25 due to restricted data quality on maternal education below 26 years of age. The time series starts in 2016 because data collection on maternal education changed in 2016.*

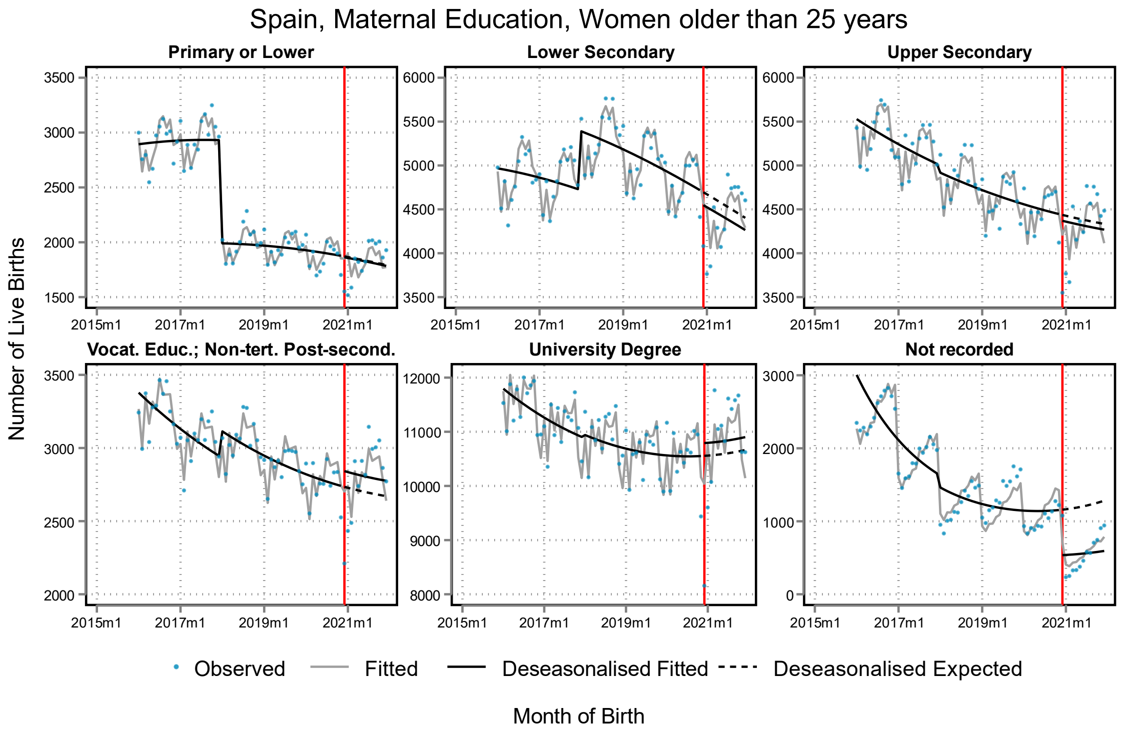

*Supplementary Figure 52: Observed and expected monthly number of live births in **Spain** by maternal in detail. Expected numbers are estimated by subgroup-specific Poisson regression modes on the full time series including an indicator variable for the exposed period (starting December 2020 to December 2021) to estimate the average effect of the COVID-19 pandemic over the entire period; a linear and a quadratic term for month of live birth to capture potential non-linearities in the secular time trends; month of the year fixed effects to account for seasonality; an indicator variable for 2016 and 2017 to capture changes in data collection on maternal education. Data is restricted to women older than 25 due to restricted data quality on maternal education below 26 years of age. The time series starts in 2016 because data collection on maternal education changed in 2016.*

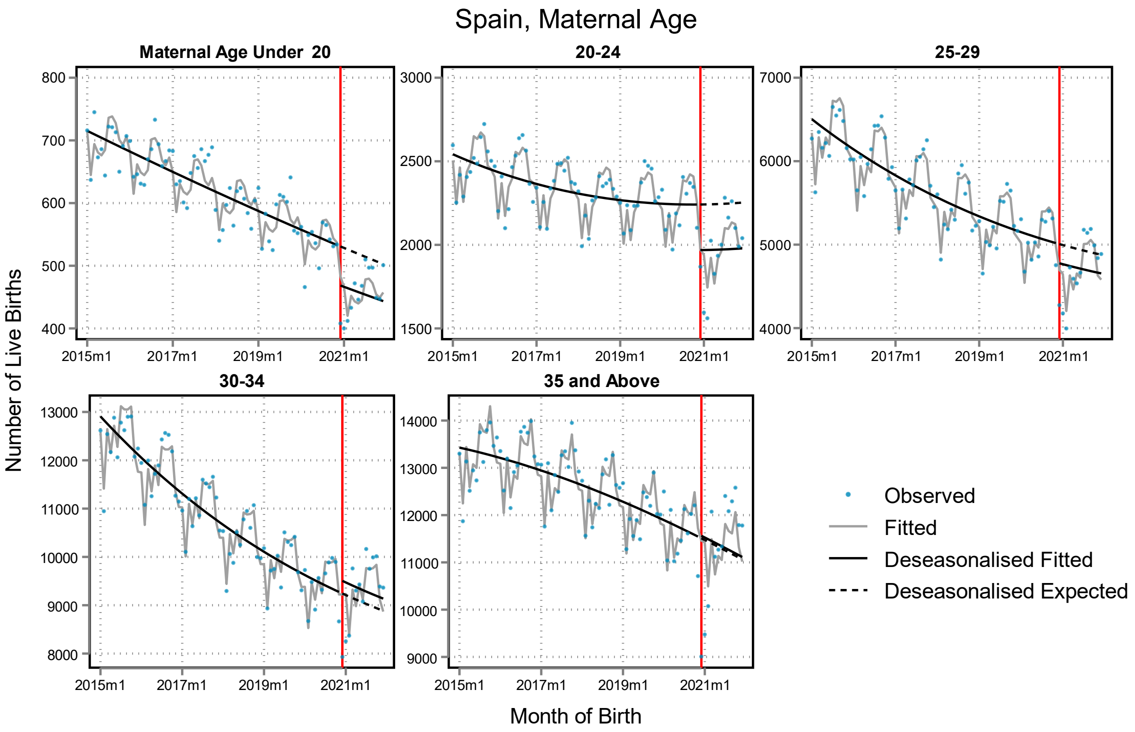

Supplementary Figure 53: Observed and expected monthly number of live births in ***Spain*** *by maternal age. Expected numbers are estimated by subgroup-specific Poisson regression modes on the full time series including an indicator variable for the exposed period (starting December 2020 to December 2021) to estimate the average effect of the COVID-19 pandemic over the entire period; a linear and a quadratic term for month of live birth to capture potential non-linearities in the secular time trends; month of the year fixed effects to account for seasonality.*

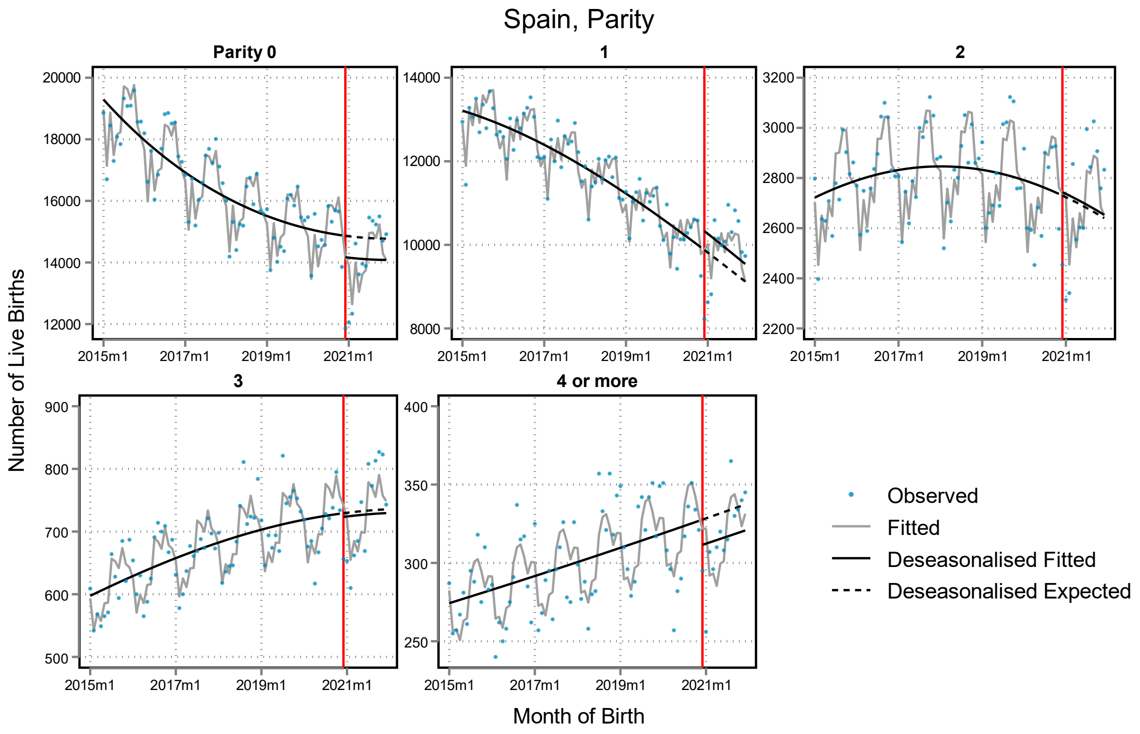

*Supplementary Figure 54: Observed and expected monthly number of live births in **Spain** by parity. Expected numbers are estimated by subgroup-specific Poisson regression modes on the full time series including an indicator variable for the exposed period (starting December 2020 to December 2021) to estimate the average effect of the COVID-19 pandemic over the entire period; a linear and a quadratic term for month of live birth to capture potential non-linearities in the secular time trends; month of the year fixed effects to account for seasonality.*

Supplementary Table 13: Relative and Percentage Point Differences in the Composition of the December 2020 – December 2021 Birth Cohort in Spain. "Observed" is abbreviated by "OBS" and "Counterfactual" is abbreviated by "CF". Statistical methods for the estimations are described in the main manuscript.

Characteristic	Observed (OBS) Births	Counterfactual (CF) Births	95%CI: CF Births	OBS - CF Births	95%CI: OBS - CF Births	% more/ less than CF	95%CI: % more/less than CF	OBS proportion	CF proportion	OBS - CF proportion	95%CI: OBS - CF proportion
**Maternal education detailed**											
Primary or Lower	23708	23813	(23223; 24403)	-105	(-768; 558)	-0.4	(-2.8; 2.1)	7.4	7.3	0.1	(-0.1; 0.3)
Lower Secondary	57285	59152	(58213; 60090)	-1867	(-2915; -818)	-3.2	(-4.7; -1.6)	17.8	18.0	-0.2	(-0.4; 0.1)
Upper Secondary	55956	56812	(55886; 57738)	-856	(-1892; 179)	-1.5	(-3.1; 0.1)	17.4	17.3	0.1	(-0.1; 0.4)
Vocational Education; Non-Tertiary Postsecondary	36343	34968	(34244; 35691)	1375	(561; 2190)	3.9	(1.8; 6.1)	11.3	10.7	0.7	(0.5; 0.9)
University Degree	140132	137070	(135605; 138536)	3062	(1423; 4700)	2.2	(1.2; 3.3)	43.7	41.8	1.9	(1.5; 2.2)
Unknown	7507	16213	(15684; 16743)	-8706	(-9262; -8150)	-53.7	(-55.2; -52.1)	2.3	4.9	-2.6	(-2.8; -2.4)
**total**	320931	328028		-7097		0.0		100.0	100.0	0.0	
**Highest Parental Education**											
No Highschool Diploma	60253	65610	(64621; 66600)	-5357	(-6457; -4257)	-8.2	(-9.5; -6.8)	18.8	20.0	-1.3	(-1.5; -1.0)
Upper Secondary	55173	58507	(57565; 59450)	-3334	(-4383; -2286)	-5.7	(-7.2; -4.2)	17.2	17.9	-0.7	(-0.9; -0.4)
Post-secondary; Tertiary	199037	197096	(195350; 198842)	1941	(-12; 3894)	1.0	(0.1; 1.9)	62.0	60.2	1.8	(1.5; 2.2)
Unknown	6468	6201	(5901; 6502)	267	(-73; 606)	4.3	(-0.5; 9.6)	2.0	1.9	0.1	(0.0; 0.2)
**total**	320931	327415		-6484		0.0		100.0	100.0	0.0	
**Maternal age**											
Below 20	5940	6730	(6457; 7003)	-790	(-1102; -478)	-11.7	(-15.2; -8.0)	1.6	1.8	-0.2	(-0.3; -0.1)
20-24	25650	29199	(28608; 29791)	-3549	(-4219; -2879)	-12.2	(-13.9; -10.3)	7.1	8.0	-0.9	(-1.1; -0.8)
25-29	61191	64132	(63280; 64984)	-2941	(-3922; -1961)	-4.6	(-5.8; -3.3)	17.0	17.6	-0.7	(-0.9; -0.4)
30-34	120804	117473	(116337; 118608)	3331	(2008; 4655)	2.8	(1.9; 3.8)	33.5	32.3	1.2	(1.0; 1.5)
Above 34	147285	146615	(145327; 147902)	670	(-821; 2161)	0.5	(-0.4; 1.3)	40.8	40.3	0.6	(0.3; 0.8)

**total**	360870	364149		-3279		0.0		100.0	100.0	0.0	
**Parity**											
0	183488	192491	(191000; 193982)	-9003	(-10714; -7292)	-4.7	(-5.4; -3.9)	50.8	52.8	-2.0	(-2.3; -1.7)
1	128705	123052	(121910; 124193)	5653	(4312; 6994)	4.6	(3.6; 5.6)	35.7	33.8	1.9	(1.6; 2.1)
2	35087	34916	(34278; 35554)	171	(-565; 907)	0.5	(-1.3; 2.4)	9.7	9.6	0.1	(-0.0; 0.3)
3	9469	9546	(9200; 9892)	-77	(-472; 318)	-0.8	(-4.3; 2.9)	2.6	2.6	0.0	(-0.1; 0.1)
4 or more	4121	4334	(4098; 4569)	-213	(-480; 54)	-4.9	(-9.8; 0.6)	1.1	1.2	0.0	(-0.1; 0.0)
**total**	360870	364339		-3469		0.0		100.0	100.0	0.0	

Sweden

Data

For Sweden (2015-2021; n= 804,502 live births), we used individual-level data from the total population register (Registret över totalbefolkningen) (births, maternal age), the multigenerational register (Flergenerationsregistret) (links between children and mothers), the tax register (Inkomst- och taxeringsregistret) (disposable income per consumption unit in the household), and the educational register (Utbildningsregistret) (maternal education). Access to Swedish register data for the purposes of this study was made possible through participation in the SWECOV project. The ethical permit was granted by the Swedish Ethical Review Authority (Permit 2024-02342-02).

Quintiles of equivalised household incomes are based on the household income distribution of women aged 15–49 and are lagged by two years before the year of birth. This lag in household income avoids that our estimated compositional change is driven by pandemic-induced change in the income distribution (due to, e.g., income losses) instead of pandemic-induced change in fertility behaviour. This, however, caused some missing values as households that have not lived in Sweden two years before birth did not receive income in Sweden that would be captured by the registers.

To protect anonymity in line with data providers' policies, we set the number of weekly births to 5 if it was positive and below 5.

For equivalisation of household income, we used the definition by statistics Sweden (see https://www.scb.se/contentassets/0521204f13e649299dec73f091e691e0/lisa-bakgrundsfakta-1990-2017.pdf on page 81). This definition uses the following weights for household members:

First adult: 1, cohabiting adult: 0.51, additional adult: 0.60 (these are adults that are not in a relationship with the first adult, i.e. parents, adult children (over 19 years) or other relatives), first child (0-19 yrs): 0.52, additional children: 0.42.

The mother's highest completed formal education was grouped into compulsory education, upper secondary education, bachelor's degree, and master's and Doctoral degree at year of birth following ISCED-classification. Maternal education is missing for women which received their education outside Sweden and have not registered their educational level in Sweden.

Apart from compositional changes in the household income composition and the maternal age, we analysed compositional changes regarding maternal age.

Results

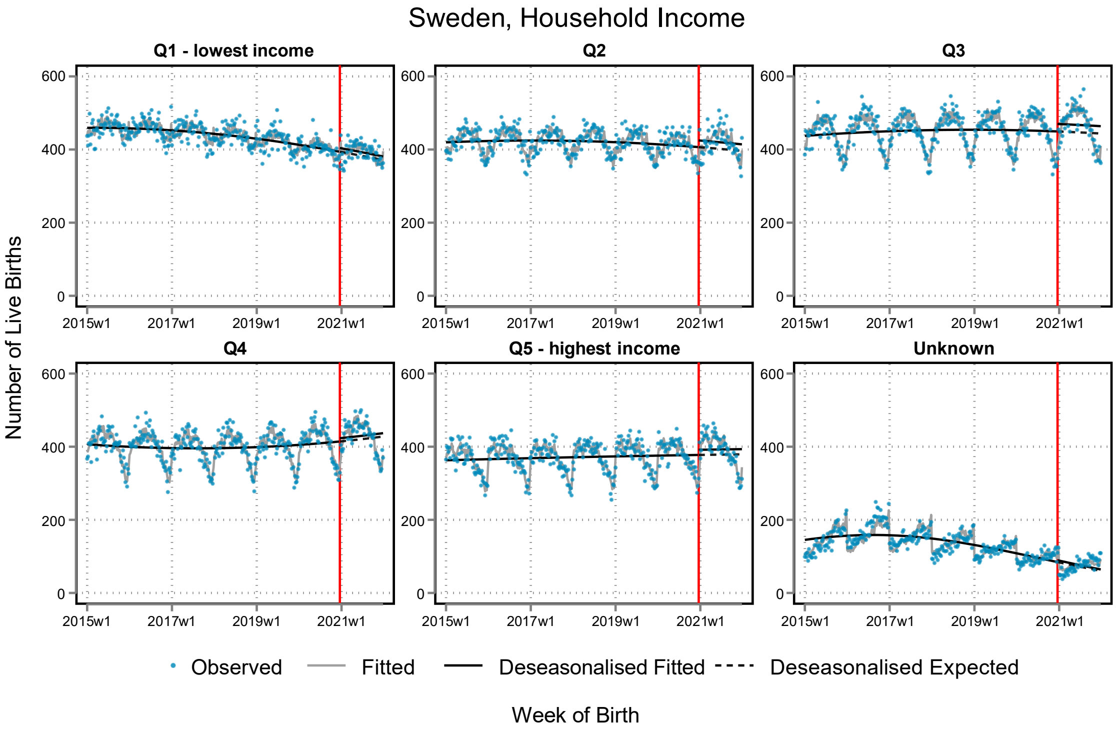

*Supplementary Figure 55: Observed and expected weekly number of live births in **Sweden** by quintile of equivalised two-year lagged household income among women aged 15-49. Expected numbers are estimated by subgroup-specific Poisson regression modes on the full time series including an indicator variable for the exposed period (starting second week of December 2020 to December 2021) to estimate the average effect of the COVID-19 pandemic over the entire period; a linear and a quadratic term for week of live birth to capture potential non-linearities in the secular time trends; week of the year fixed effects to account for seasonality. Unknown income occurs for households which were not filing tax at the time of measurement two years prior to birth.*

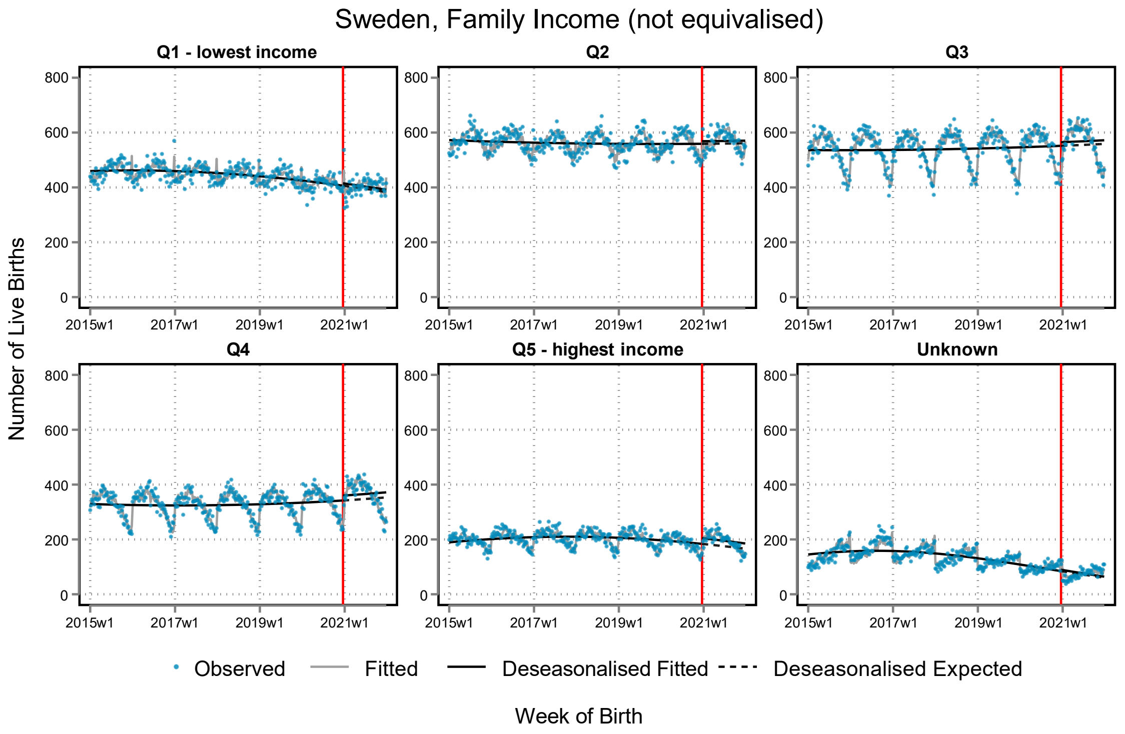

Supplementary Figure 56: Observed and expected weekly number of live births in ***Sweden*** *by quintile of non-equivalised two-year lagged household income among women aged 15-49. Expected numbers are estimated by subgroup-specific Poisson regression modes on the full time series including an indicator variable for the exposed period (starting second week of December 2020 to December 2021) to estimate the average effect of the COVID-19 pandemic over the entire period; a linear and a quadratic term for week of live birth to capture potential non-linearities in the secular time trends; week of the year fixed effects to account for seasonality. Unknown income occurs for households which were not filing tax at the time of measurement two years prior to birth.*

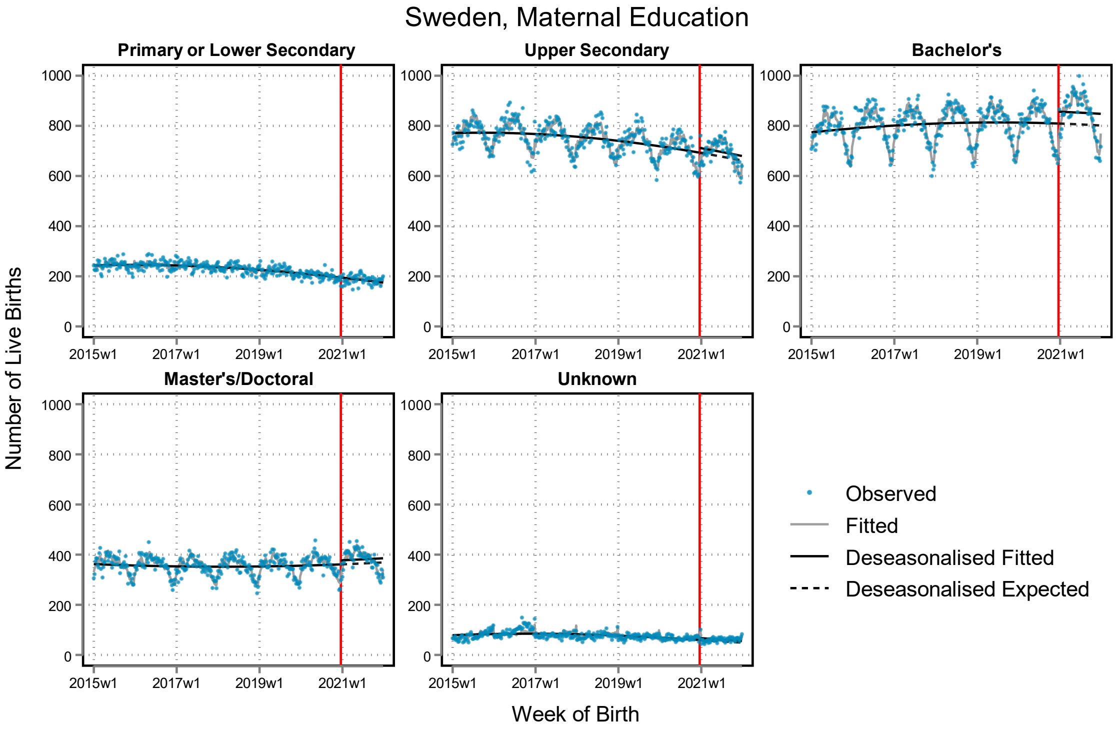

*Supplementary Figure 57: Observed and expected weekly number of live births in **Sweden** by maternal education. Expected numbers are estimated by subgroup-specific Poisson regression modes on the full time series including an indicator variable for the exposed period (starting second week of December 2020 to December 2021) to estimate the average effect of the COVID-19 pandemic over the entire period; a linear and a quadratic term for week of live birth to capture potential non-linearities in the secular time trends; week of the year fixed effects to account for seasonality. Unknown values occur if, for example, education was not received in Sweden but abroad.*

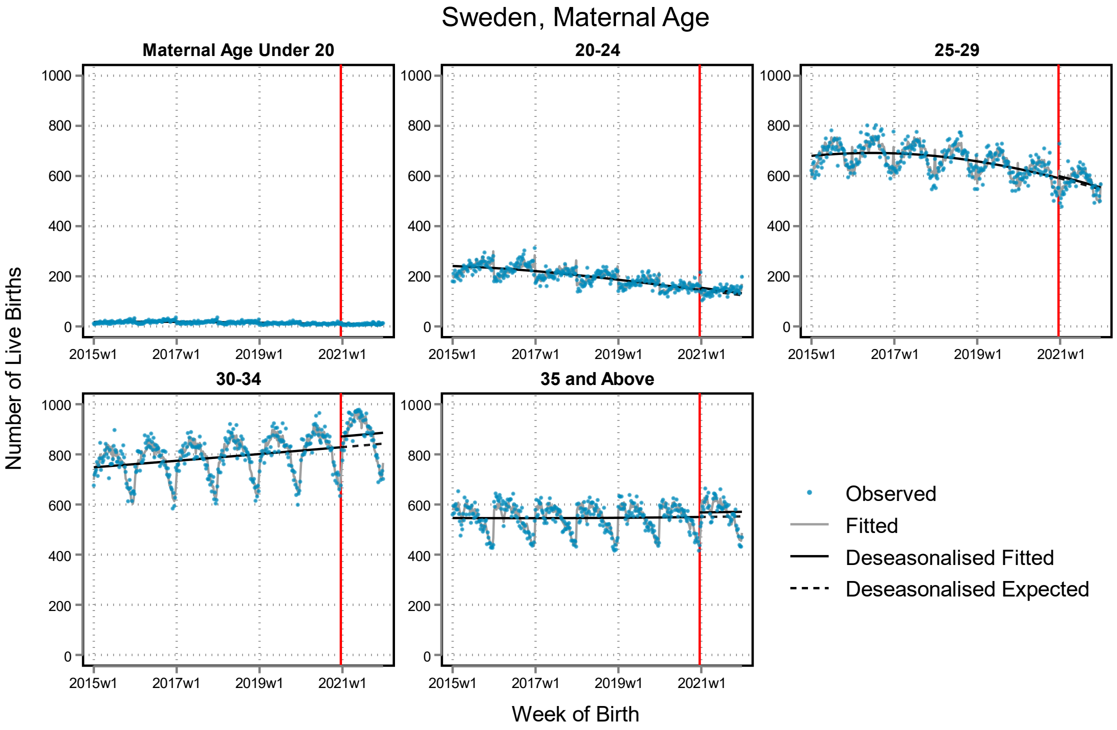

*Supplementary Figure 58: Observed and expected weekly number of live births in **Sweden** by maternal age. Expected numbers are estimated by subgroup-specific Poisson regression modes on the full time series including an indicator variable for the exposed period (starting second week of December 2020 to December 2021) to estimate the average effect of the COVID-19 pandemic over the entire period; a linear and a quadratic term for week of live birth to capture potential non-linearities in the secular time trends; week of the year fixed effects to account for seasonality.*

Supplementary Table 14: Relative and Percentage Point Differences in the Composition of the December 2020 – December 2021 Birth Cohort in Sweden. "Observed" is abbreviated by "OBS" and "Counterfactual" is abbreviated by "CF". Statistical methods for the estimations are described in the main manuscript.

Characteristic	Observed (OBS) Births	Counterfactual (CF) Births	95%CI: CF Births	OBS - CF Births	95%CI: OBS - CF Births	% more/less than CF	95%CI: % more/less than CF	OBS proportion	CF proportion	OBS - CF proportion	95%CI: OBS - CF proportion
**Household Income**											
Lowest 20%	21818	21362	(20890; 21834)	456	(-98; 1010)	2.1	(-0.1; 4.4)	18.6	18.9	-0.2	(-0.6; 0.1)
Q2	30704	30194	(29611; 30776)	510	(-165; 1186)	1.7	(-0.2; 3.7)	26.2	26.7	-0.5	(-0.9; -0.0)
Q3	30451	29744	(29163; 30325)	707	(33; 1382)	2.4	(0.4; 4.4)	26.0	26.3	-0.3	(-0.7; 0.2)
Q4	19539	18566	(18103; 19029)	973	(435; 1511)	5.2	(2.7; 7.9)	16.7	16.4	0.3	(-0.1; 0.6)
Highest 20%	10482	9417	(9109; 9724)	1065	(698; 1433)	11.3	(7.8; 15.1)	8.9	8.3	0.6	(0.4; 0.9)
Missing income information	4143	3914	(3741; 4086)	229	(15; 443)	5.9	(1.4; 10.7)	3.5	3.5	0.1	(-0.1; 0.2)
Total	117137	113196		3941		3.5		100.0	100.0	0.0	
**Maternal Education**											
Compulsory	10027	10030	(9716; 10344)	-3	(-373; 368)	0.0	(-3.1; 3.2)	8.6	8.9	-0.4	(-0.6; -0.1)
Upper Secondary	37504	36506	(35885; 37126)	998	(271; 1726)	2.7	(1.0; 4.5)	32.0	32.5	-0.5	(-0.9; -0.0)
Bachelor's Degree	45818	43302	(42607; 43997)	2516	(1704; 3327)	5.8	(4.1; 7.5)	39.1	38.5	0.6	(0.1; 1.1)
Master's / Doctorate	20459	19580	(19108; 20052)	879	(330; 1428)	4.5	(2.0; 7.1)	17.5	17.4	0.0	(-0.4; 0.4)
Unkown	3329	2956	(2793; 3118)	373	(175; 571)	12.6	(6.8; 19.2)	2.8	2.6	0.2	(0.1; 0.4)
**total**	117137	112373		4764		4.2		100.0	100.0	0.0	
**Maternal age**											
Below 20	473	414	(358; 470)	59	(-11; 129)	14.2	(0.6; 32.0)	0.4	0.4	0.0	(-0.0; 0.1)
20-24	7776	7319	(7063; 7575)	457	(148; 766)	6.2	(2.6; 10.1)	6.6	6.5	0.2	(-0.1; 0.4)
25-29	31170	30734	(30174; 31294)	436	(-223; 1094)	1.4	(-0.4; 3.3)	26.6	27.2	-0.6	(-1.0; -0.2)
30-34	47148	44837	(44118; 45556)	2311	(1475; 3146)	5.2	(3.5; 6.9)	40.3	39.7	0.5	(0.0; 1.0)
Above 34	30560	29576	(29001; 30151)	984	(314; 1654)	3.3	(1.4; 5.4)	26.1	26.2	-0.1	(-0.5; 0.3)
**total**	117127	112881		4246		3.8		100.0	100.0	0.0	

United States

Data

For the US (2015-2021; n=26,597,063 live births), we used openly available aggregated time series data on the number of monthly births by maternal and paternal education, age, and parity requested through the Centers for Disease Control and Prevention WONDER portal (https://wonder.cdc.gov/natality.html) that provides access to the Natality online databases. The data is generated by birth certificates and capture births occurring in the United States from US residents.

Since 2016, all reporting areas are using the 2003 US Standard Certification of Live Birth (see data description: https://wonder.cdc.gov/wonder/help/natality-expanded.html). Thus, our time series data for maternal and paternal education are covering 2016-2021. Parental education is assigned to 8 levels: 1) 8th grade or less, 2) 9th through 12th grade with no diploma, 3) high school graduate or GED completed, 4) some college credit, but no degree, 5) associate degree (AA, AS), 6) Bachelor's degree (BA, AB, BS), 7) Master's degree (MA, MS), 8) Doctorate (PhD, EdD) or professional degree (MD, DDS, DVM, LLB, JD). We grouped levels 1,2, to "No Highschool Diploma", levels 3,4 to "Upper Secondary", and levels 5-8 to "post-secondary and tertiary education".

We analysed change in socioeconomic composition regarding maternal and paternal education. In addition, we provide results for more detailed measures of maternal education, maternal age, and parity. Analyses regarding maternal age and parity are using data covering 2015-2021.

Results

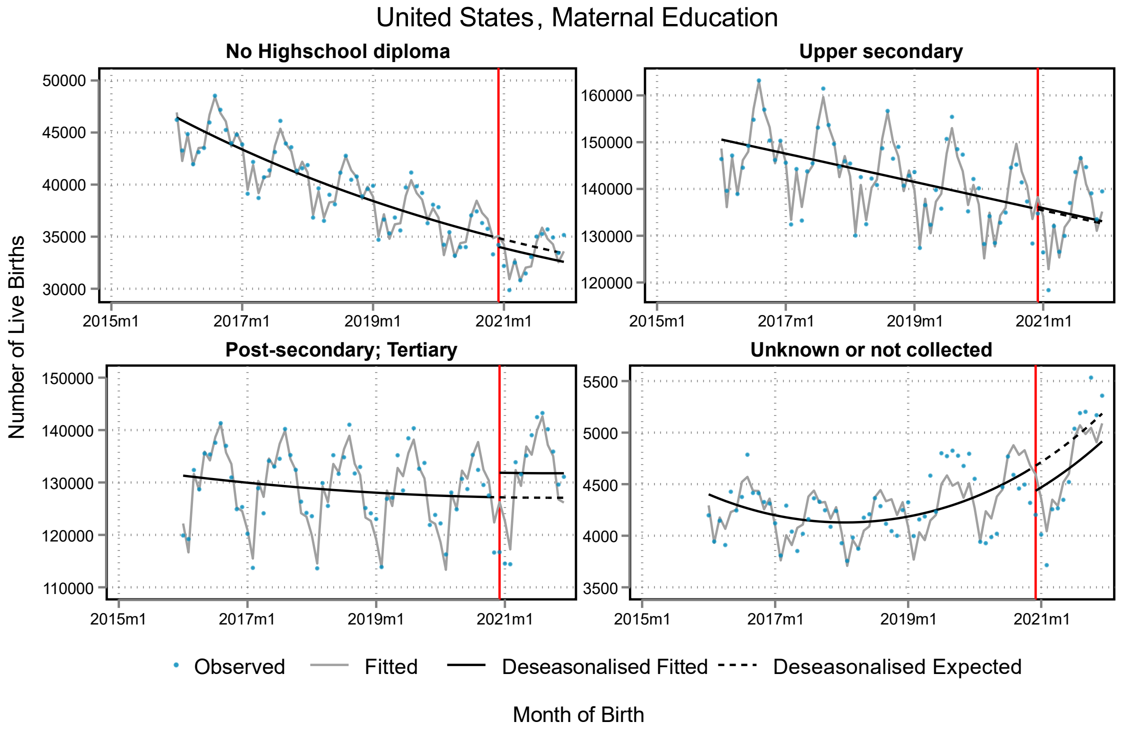

*Supplementary Figure 59: Observed and expected monthly number of live births in **the United States** by maternal education (primary socioeconomic indicator). Expected numbers are estimated by subgroup-specific Poisson regression modes on the full time series including an indicator variable for the exposed period (starting December 2020 to December 2021) to estimate the average effect of the COVID-19 pandemic over the entire period; a linear and a quadratic term for month of live birth to capture potential non-linearities in the secular time trends; month of the year fixed effects to account for seasonality. Time series is restricted to 2016-2021 as birth certificates were not standardised regarding education across all states before 2016.*

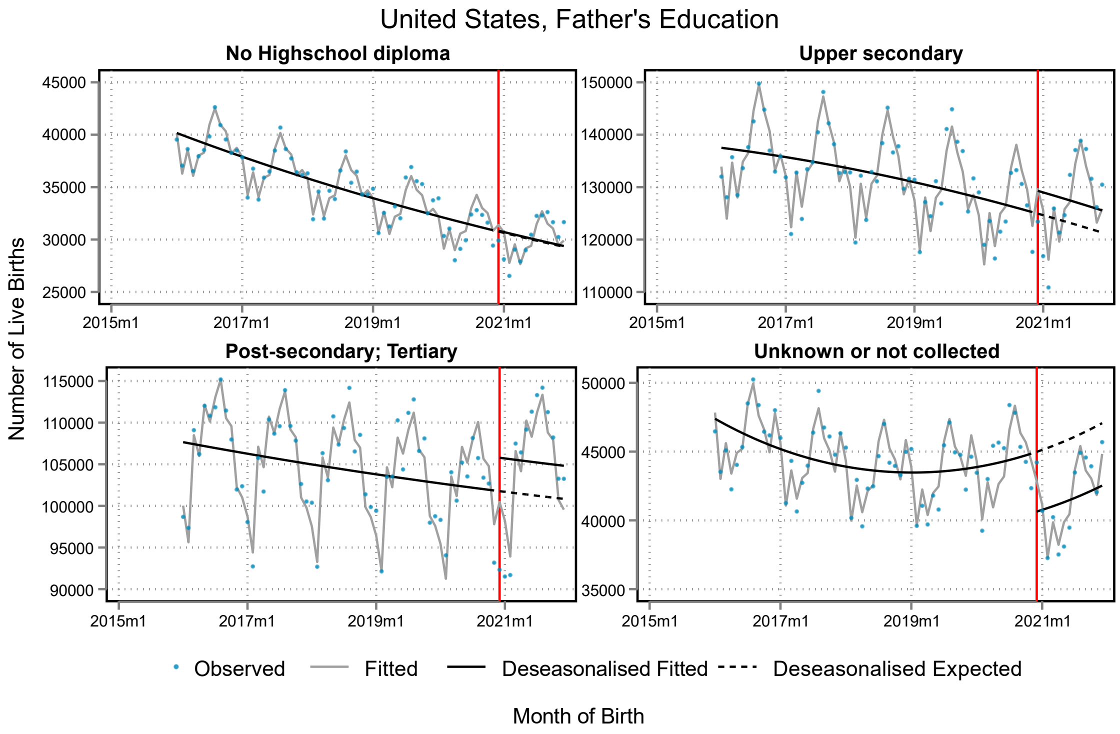

Supplementary Figure 60: Observed and expected monthly number of live births in ***the United States*** *by paternal education in detail. Expected numbers are estimated by subgroup-specific Poisson regression modes on the full time series including an indicator variable for the exposed period (starting December 2020 to December 2021) to estimate the average effect of the COVID-19 pandemic over the entire period; a linear and a quadratic term for month of live birth to capture potential non-linearities in the secular time trends; month of the year fixed effects to account for seasonality. Time series is restricted to 2016-2021 as birth certificates were not standardised regarding education across all states before 2016.*

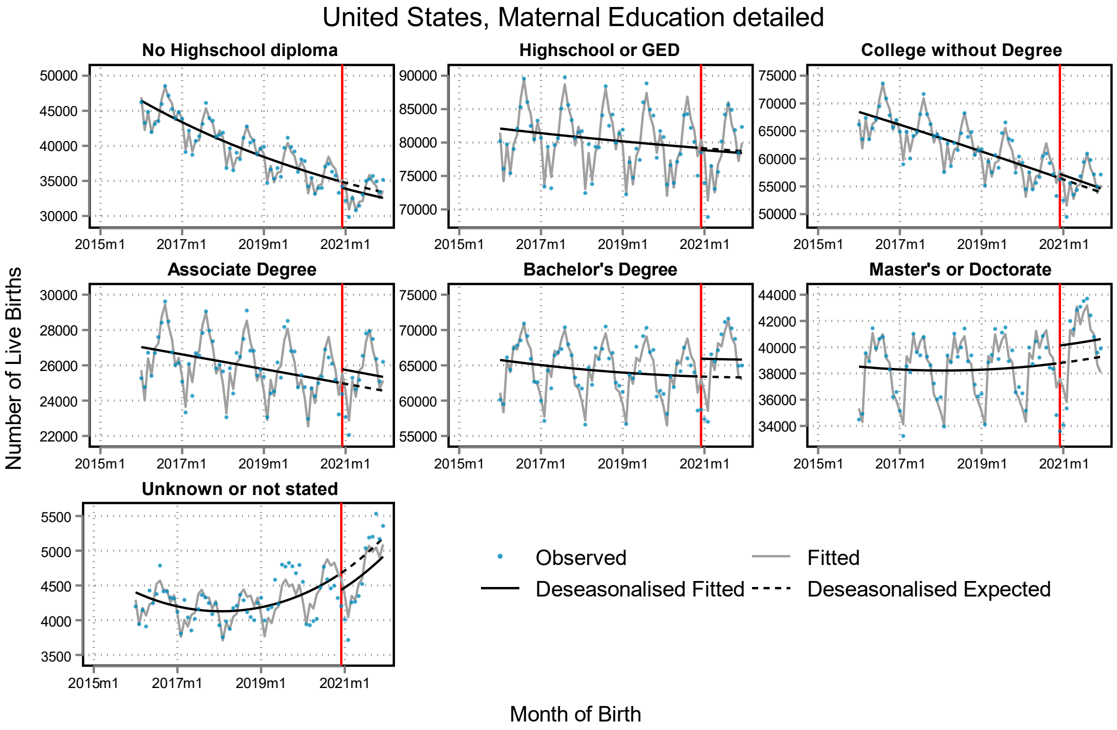

Supplementary Figure 61: Observed and expected monthly number of live births in ***the United States*** *by maternal education in detail. Expected numbers are estimated by subgroup-specific Poisson regression modes on the full time series including an indicator variable for the exposed period (starting December 2020 to December 2021) to estimate the average effect of the COVID-19 pandemic over the entire period; a linear and a quadratic term for month of live birth to capture potential non-linearities in the secular time trends; month of the year fixed effects to account for seasonality. Time series is restricted to 2016-2021 as birth certificates were not standardised across all states before 2016 regarding education.*

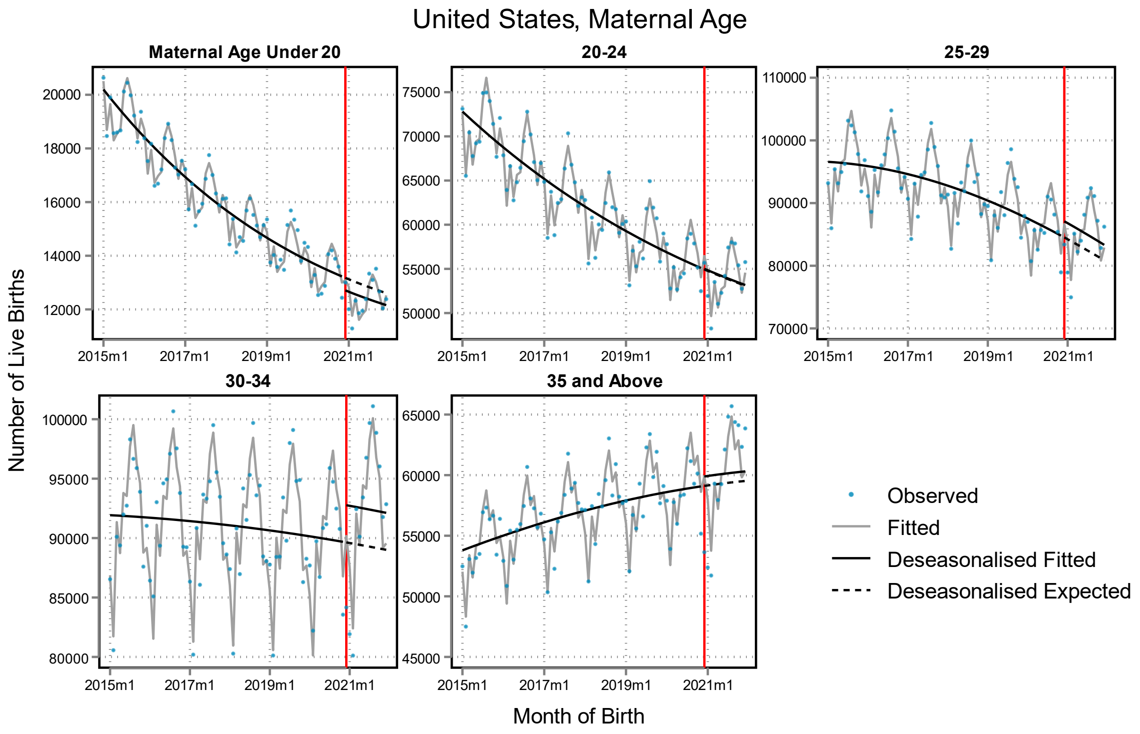

Supplementary Figure 62: Observed and expected monthly number of live births in ***the United States*** *by maternal age. Expected numbers are estimated by subgroup-specific Poisson regression modes on the full time series including an indicator variable for the exposed period (starting December 2020 to December 2021) to estimate the average effect of the COVID-19 pandemic over the entire period; a linear and a quadratic term for month of live birth to capture potential non-linearities in the secular time trends; month of the year fixed effects to account for seasonality.*

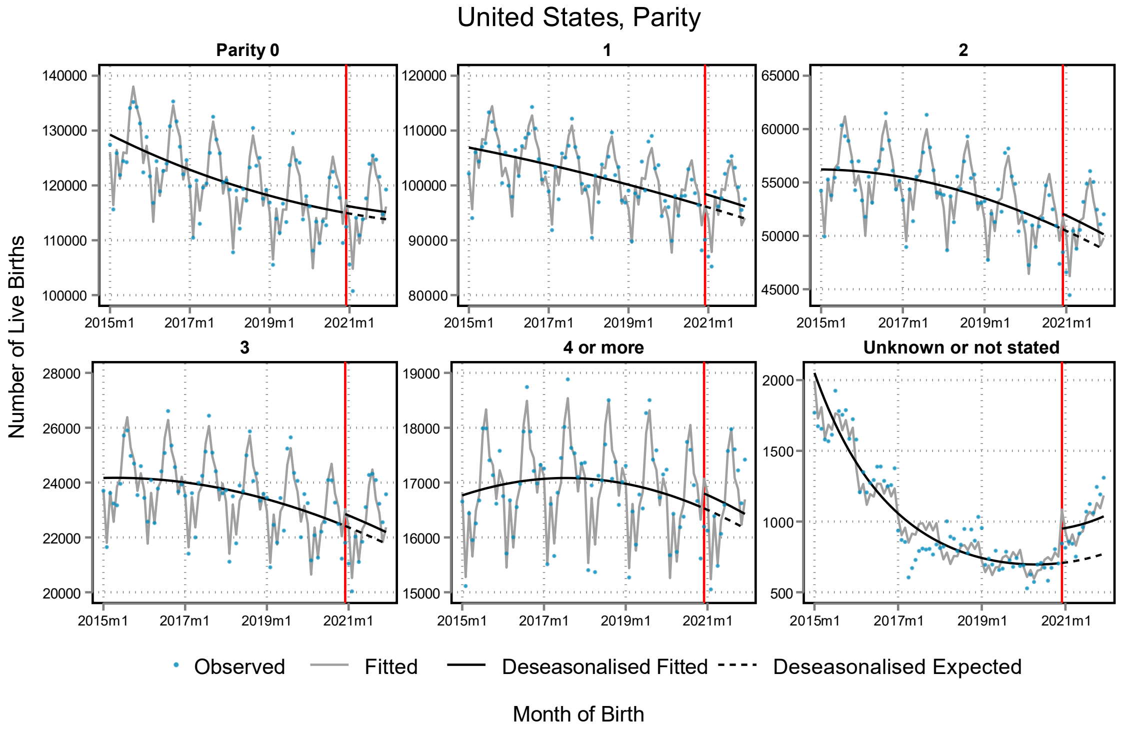

Supplementary Figure 63: Observed and expected monthly number of live births in ***the United States*** *by parity. Expected numbers are estimated by subgroup-specific Poisson regression modes on the full time series including an indicator variable for the exposed period (starting December 2020 to December 2021) to estimate the average effect of the COVID-19 pandemic over the entire period; a linear and a quadratic term for month of live birth to capture potential non-linearities in the secular time trends; month of the year fixed effects to account for seasonality.*

Supplementary Table 15: Relative and Percentage Point Differences in the Composition of the December 2020 – December 2021 Birth Cohort in the United States. "Observed" is abbreviated by "OBS" and "Counterfactual" is abbreviated by "CF". Statistical methods for the estimations are described in the main manuscript.

Characteristic	Observed (OBS) Births	Counterfactual (CF) Births	95%CI: CF Births	OBS - CF Births	95%CI: OBS - CF Births	% more/less than CF	95%CI: % more/less than CF	OBS proportion	CF proportion	OBS - CF proportion	95%CI: OBS - CF proportion
**Paternal Education**											
Primary or Lower Secondary	391727	390712	(388345; 393080)	1015	(-1652; 3681)	0.3	(-0.3; 0.9)	9.9	10.0	-0.1	(-0.2; -0.0)
Upper Secondary	1656827	1601082	(1596193; 1605971)	55745	(50244; 61246)	3.5	(3.2; 3.8)	41.9	41.0	0.9	(0.8; 1.0)
Post-secondary; Tertiary	1363477	1311741	(1307268; 1316213)	51737	(46712; 56761)	3.9	(3.6; 4.3)	34.5	33.6	0.9	(0.8; 1.0)
Unknown	542139	600088	(596954; 603222)	-57949	(-61399; -54498)	-9.7	(-10.1; -9.2)	13.7	15.4	-1.7	(-1.7; -1.6)
**total**	3954170	3903623		50548		0.0		100.0	100.0	0.0	
**Maternal Education in Detail**											
No Highschool Diploma	433594	444395	(441864; 446927)	-10801	(-13643; -7960)	-2.4	(-3.0; -1.9)	11.0	11.4	-0.4	(-0.5; -0.4)
Highschool or GED	1024110	1027800	(1023814; 1031787)	-3690	(-8143; 762)	-0.4	(-0.7; 0.0)	25.9	26.3	-0.4	(-0.5; -0.4)
College without Degree	727975	718146	(714915; 721376)	9829	(6192; 13467)	1.4	(0.9; 1.8)	18.4	18.4	0.0	(-0.1; 0.1)
Associate Degree	332040	321848	(319640; 324056)	10192	(7712; 12672)	3.2	(2.5; 3.9)	8.4	8.2	0.1	(0.1; 0.2)
Bachelor's Degree	853424	820678	(817123; 824233)	32746	(28756; 36735)	4.0	(3.5; 4.4)	21.6	21.0	0.5	(0.5; 0.6)
Master's or Doctorate	522217	504846	(502031; 507662)	17371	(14219; 20522)	3.4	(2.9; 4.0)	13.2	12.9	0.3	(0.2; 0.3)
Unknown	60810	64144	(63088; 65199)	-3334	(-4495; -2172)	-5.2	(-6.7; -3.6)	1.5	1.6	-0.1	(-0.1; -0.1)
**total**	3954170	3901857		52313		0.0		100.0	100.0	0.0	
**Maternal age**											
Below 20	161848	167798	(166447; 169149)	-5950	(-7514; -4385)	-3.5	(-4.3; -2.8)	4.1	4.3	-0.2	(-0.3; -0.2)
20-24	704171	703197	(700390; 706004)	974	(-2280; 4227)	0.1	(-0.3; 0.5)	17.8	18.1	-0.3	(-0.4; -0.3)
25-29	1107375	1074653	(1071169; 1078138)	32722	(28672; 36771)	3.0	(2.7; 3.4)	28.0	27.7	0.3	(0.2; 0.4)
30-34	1199221	1158734	(1155023; 1162444)	40487	(36201; 44774)	3.5	(3.2; 3.8)	30.3	29.9	0.4	(0.3; 0.5)
Above 34	781555	771327	(768247; 774408)	10228	(6693; 13762)	1.3	(0.9; 1.7)	19.8	19.9	-0.1	(-0.2; -0.1)

**total**	3954170	3875709		78461		0.0		100.0	100.0	0.0	
**Parity**											
0	1504918	1487844	(1483665; 1492022)	17075	(12254; 21895)	1.1	(0.9; 1.4)	38.1	38.4	-0.3	(-0.4; -0.2)
1	1262823	1233806	(1230027; 1237584)	29017	(24644; 33391)	2.4	(2.0; 2.7)	31.9	31.8	0.1	(0.0; 0.2)
2	664089	645530	(642815; 648245)	18559	(15409; 21709)	2.9	(2.4; 3.3)	16.8	16.6	0.1	(0.1; 0.2)
3	293024	287445	(285620; 289269)	5579	(3469; 7690)	1.9	(1.3; 2.6)	7.4	7.4	0.0	(-0.0; 0.0)
4 or more	216324	212833	(211250; 214416)	3491	(1664; 5318)	1.6	(0.9; 2.4)	5.5	5.5	0.0	(-0.1; 0.0)
Unknown	12992	9675	(9347; 10002)	3317	(2921; 3714)	34.3	(29.9; 39.0)	0.3	0.2	0.1	(0.1; 0.1)
**total**	3954170	3877132		77038		0.0		100.0	100.0	0.0	

Wales

Data

For Wales (2015-2021; n=216,797 live births), we purchased monthly time series (2015-2022) of the number of live births by deciles of the Index for Multiple Deprivation (IMD) from the Office for National Statistics. These data are now openly available due to our purchase.

(https://www.ons.gov.uk/peoplepopulationandcommunity/birthsdeathsandmarriages/livebirths/adhocs/1703livebirthsbymonthofoccurrenceandimddecileenglandandwales2015to2022)

These data are considered to cover 100% of live births and there are only a low number of late registrations which, for 2021, are already captured in our data.

The IMD is created for Lower Super Output Areas (LSOAs) of mothers' residence. IMD deciles for 2015 are based on 2015 census data and IMD deciles for 2016-2022 are based on 2019 census data.

We use quintiles of the IMD as primary indicator of socioeconomic circumstances but show results also for deciles.

Results

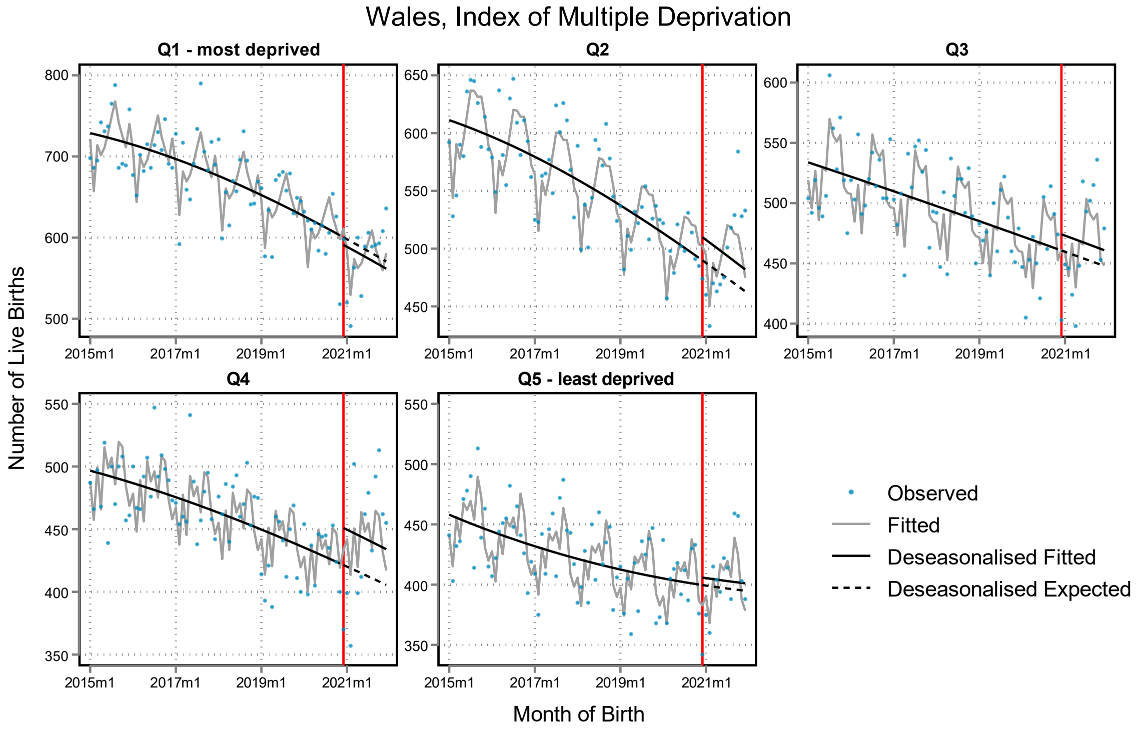

*Supplementary Figure 64: Observed and expected monthly number of live births in **Wales** by primary indicator of socioeconomic circumstances (area deprivation in Lower Super Output Areas of maternal residence). Expected numbers are estimated by subgroup-specific Poisson regression modes on the full time series including an indicator variable for the exposed period (starting December 2020 to December 2021) to estimate the average effect of the COVID-19 pandemic over the entire period; a linear and a quadratic term for month of live birth to capture potential non-linearities in the secular time trends; month of the year fixed effects to account for seasonality.*

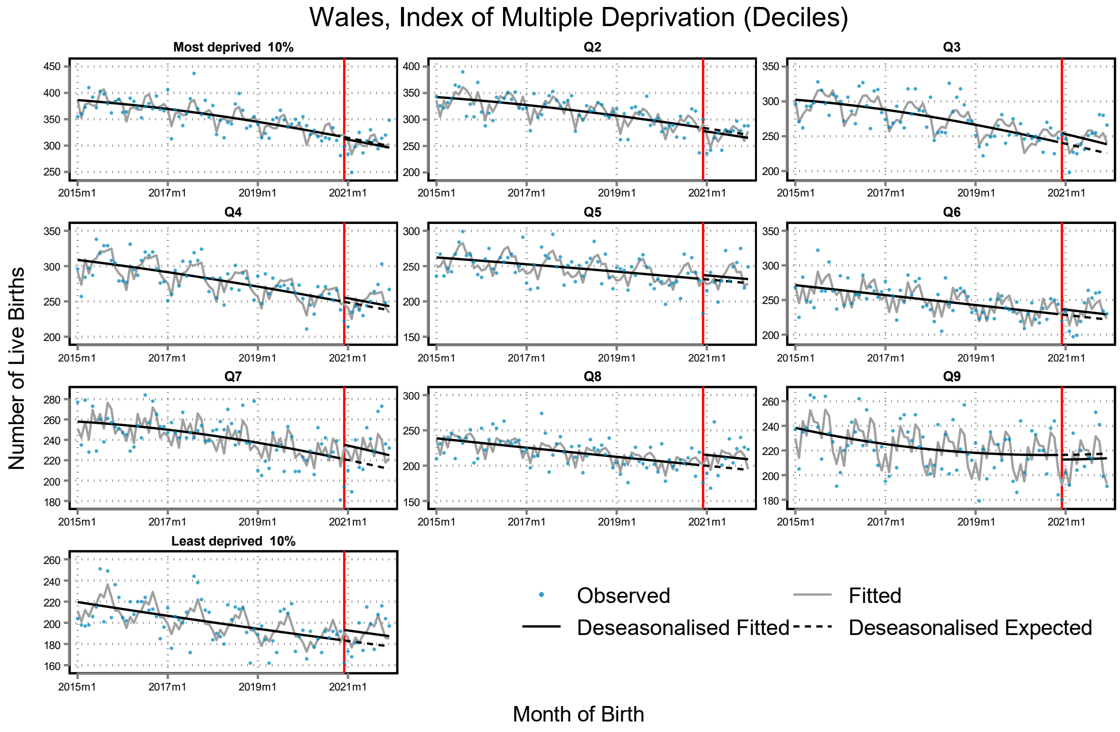

Supplementary Figure 65: Observed and expected monthly number of live births in ***Wales*** *by decile of the Index of Multiple Deprivation of Lower Super Output Area of maternal residential location. Expected numbers are estimated by subgroup-specific Poisson regression modes on the full time series including an indicator variable for the exposed period (starting December 2020 to December 2021) to estimate the average effect of the COVID-19 pandemic over the entire period; a linear and a quadratic term for month of live birth to capture potential non-linearities in the secular time trends; month of the year fixed effects to account for seasonality.*

Supplementary Table 16: Relative and Percentage Point Differences in the Composition of the December 2020 – December 2021 Birth Cohort in Wales. "Observed" is abbreviated by "OBS" and "Counterfactual" is abbreviated by "CF". Statistical methods for the estimations are described in the main manuscript.

Characteristic	Observed (OBS) Births	Counterfactual (CF) Births	95%CI: CF Births	OBS - CF Births	95%CI: OBS - CF Births	% more/less than CF	95%CI: % more/less than CF	OBS proportion	CF proportion	OBS - CF proportion	95%CI: OBS - CF proportion
**Index of Multiple Deprivation**											
D1 - Most Deprived	3965	4008	(3797; 4220)	-43	(-288; 201)	-1.1	(-6.0; 4.4)	12.8	13.3	-0.5	(-1.1; 0.2)
D2	3547	3623	(3421; 3825)	-76	(-309; 157)	-2.1	(-7.3; 3.7)	11.5	12.0	-0.5	(-1.2; 0.1)
D3	3205	3030	(2848; 3212)	175	(-38; 388)	5.8	(-0.2; 12.5)	10.3	10.0	0.3	(-0.3; 0.9)
D4	3232	3155	(2968; 3341)	77	(-140; 295)	2.5	(-3.3; 8.9)	10.4	10.4	0.0	(-0.6; 0.6)
D5	3042	2968	(2783; 3153)	74	(-140; 288)	2.5	(-3.5; 9.3)	9.8	9.8	0.0	(-0.6; 0.6)
D6	3022	2924	(2742; 3106)	98	(-114; 309)	3.3	(-2.7; 10.2)	9.8	9.7	0.1	(-0.5; 0.6)
D7	2988	2809	(2631; 2986)	179	(-28; 387)	6.4	(0.1; 13.6)	9.6	9.3	0.4	(-0.2; 0.9)
D8	2747	2550	(2381; 2720)	197	(-1; 395)	7.7	(1.0; 15.4)	8.9	8.4	0.4	(-0.1; 1.0)
D9	2749	2798	(2616; 2980)	-49	(-258; 160)	-1.7	(-7.7; 5.1)	8.9	9.3	-0.4	(-1.0; 0.2)
D10 - Least Deprived	2472	2345	(2182; 2508)	127	(-63; 317)	5.4	(-1.4; 13.3)	8.0	7.8	0.2	(-0.3; 0.7)
**total**	30969	30210		759		0.0		100.0	100.0	0.0	

References

1. StataCorp. Stata Statistical Software: Release 18. Published online 2023.

2. Szwarcwald CL, Leal M do C, Esteves-Pereira AP, et al. Avaliação das informações do *Sistema de Informações sobre Nascidos Vivos* (SINASC), Brasil. *Cad Saúde Pública*. 2019;35:e00214918. doi:10.1590/0102-311X00214918

3. Allik M, Ramos D, Agranonik M, et al. Developing a Small-Area Deprivation Measure for Brazil. doi:10.36399/gla.pubs.215898

4. Sampson RJ. *Great American City: Chicago and the Enduring Neighborhood Effect*. Univ. of Chicago Press; 2012.

5. Toro Roa JP, Iunes RF, Mills S. *Achieving Health Outcomes in Colombia*. World Bank, Washington, DC; 2019. doi:10.1596/32538

6. Peralta A, Espinel-Flores V, Gotsens M, Pérez G, Benach J, Marí-Dell'Olmo M. Developing a deprivation index to study geographical health inequalities in Ecuador. *Revista de Saúde Pública*. 2019;53:97-97. doi:10.11606/s1518-8787.2019053001410

7. Grajales-Muñiz C, Borja-Aburto VH, Cabrera-Gaytán DA, Rojas-Mendoza T, Arriaga-Nieto L, Vallejos-Parás A. Zika virus: Epidemiological surveillance of the Mexican Institute of Social Security. *PLOS ONE*. 2019;14(2):e0212114. doi:10.1371/journal.pone.0212114
