# Supplementary material for: Parental socioeconomic composition of birth cohorts changed during the COVID-19 pandemic
Source: Nat Commun. 2025 Dec 13;16:11477. doi: 10.1038/s41467-025-66264-z (PMC12749473; doi:10.1038/s41467-025-66264-z)
Supplement: Supplementary file 2 — Reporting Summary [file 41467_2025_66264_MOESM2_ESM.pdf]

nature portfolio

Corresponding author(s): Moritz Oberndorfer

Last updated by author(s): Jun 3, 2025

Reporting Summary

Nature Portfolio wishes to improve the reproducibility of the work that we publish. This form provides structure for consistency and transparency in reporting. For further information on Nature Portfolio policies, see our Editorial Policies and the Editorial Policy Checklist.

Statistics

For all statistical analyses, confirm that the following items are present in the figure legend, table legend, main text, or Methods section.

n/a	Confirmed	
☒	☐	The exact sample size (n) for each experimental group/condition, given as a discrete number and unit of measurement
☒	☐	A statement on whether measurements were taken from distinct samples or whether the same sample was measured repeatedly
☒	☐	The statistical test(s) used AND whether they are one- or two-sided<br>*Only common tests should be described solely by name; describe more complex techniques in the Methods section.*
☒	☐	A description of all covariates tested
☒	☐	A description of any assumptions or corrections, such as tests of normality and adjustment for multiple comparisons
☒	☐	A full description of the statistical parameters including central tendency (e.g. means) or other basic estimates (e.g. regression coefficient) AND variation (e.g. standard deviation) or associated estimates of uncertainty (e.g. confidence intervals)
☒	☐	For null hypothesis testing, the test statistic (e.g. F, t, r) with confidence intervals, effect sizes, degrees of freedom and P value noted<br>*Give P values as exact values whenever suitable.*
☒	☐	For Bayesian analysis, information on the choice of priors and Markov chain Monte Carlo settings
☒	☐	For hierarchical and complex designs, identification of the appropriate level for tests and full reporting of outcomes
☒	☐	Estimates of effect sizes (e.g. Cohen's d, Pearson's r), indicating how they were calculated

Our web collection on statistics for biologists contains articles on many of the points above.

Software and code

Policy information about availability of computer code

Data collection	No code was used to collect the data. The data used in this study were collected from the respective country-specific data holder.
Data analysis	Analyses were carried out in Stata v18 and all code for the statistical analysis is openly available in a repository at https://doi.org/10.5281/zenodo.15585751 and at https://github.com/MoritzOberndorfer/COVID-19-pandemic-changed-the-socioeconomic-composition-of-parents

For manuscripts utilizing custom algorithms or software that are central to the research but not yet described in published literature, software must be made available to editors and reviewers. We strongly encourage code deposition in a community repository (e.g. GitHub). See the Nature Portfolio guidelines for submitting code & software for further information.

Data

Policy information about availability of data

All manuscripts must include a data availability statement. This statement should provide the following information, where applicable:

- Accession codes, unique identifiers, or web links for publicly available datasets
- A description of any restrictions on data availability
- For clinical datasets or third party data, please ensure that the statement adheres to our policy

Data cannot be shared by us. We used openly accessible data for Brazil, Colombia, Ecuador, Mexico, Scotland, Spain, and the United States. We purchased the necessary data for England and Wales from the Office for National Statistics (ONS) which is now openly available on their website. Links to the openly available data are provided in the respective country profiles in our supplementary material.

For Austria, Denmark, Finland, the Netherlands, South Australia, and Sweden, we used restricted access individual-level data. We are not allowed to share these data, but they can be applied for through the respective data holders mentioned in the country profiles in our supplementary material.

Research involving human participants, their data, or biological material

Policy information about studies with human participants or human data. See also policy information about sex, gender (identity/presentation), and sexual orientation and race, ethnicity and racism.

Reporting on sex and gender	Sex and gender were relevant to this study insofar as we considered both maternal and paternal education of live births where possible. This is described for each country in the supplement. Further, the role of gender norms were essentials for the interpretation and discussion of cross-country differences in the results. Separate analysis for female and male babies were not carried out because we were interested in the socioeconomic composition of the birth cohort. Research on the pandemic's effect on sex ratios was not the focus of this study and could be studied in a separate paper.
Reporting on race, ethnicity, or other socially relevant groupings	All groupings in our study are based on register-data. We study how the parental composition of births changes regarding parental income, area-deprivation, formal education, maternal age, and parity. We provide details on the measurements for each country in our country profiles.<br>We discuss the limitations of our socioeconomic indicators in the manuscript.<br>Information on race or ethnicity was not used in this study because the quality of data collection as well as the kind of information collected greatly varied between country.
Population characteristics	We use birth register data from 15 countries between 2015-2021. That means we cover the full population of births of each country to the extent the birth register includes all births in a country. Although coverage is close to 100% in all included countries, some countries have lower coverage and later registrations. We discuss these in the manuscript and the country profiles. The population characteristics for each country are presented in the manuscript and the supplementary material.
Recruitment	Participants were not recruited for this study as we used population-wide register-based data on births. Self-selection in birth register data is very limited. In countries with a non-negligible number late birth registrations, self-selection is possible. We discuss this limitation in our manuscript and in the country profiles for each country separately.
Ethics oversight	Where restricted individual-level data was accessed, we mention the respective data access permits and, where applicable, the ethical approvals. Where we used openly available data, we refer to the data provider's websites which describe the terms of use.

Note that full information on the approval of the study protocol must also be provided in the manuscript.

Field-specific reporting

Please select the one below that is the best fit for your research. If you are not sure, read the appropriate sections before making your selection.

☐ Life sciences ☒ Behavioural & social sciences ☐ Ecological, evolutionary & environmental sciences

For a reference copy of the document with all sections, see nature.com/documents/nr-reporting-summary-flat.pdf

Behavioural & social sciences study design

All studies must disclose on these points even when the disclosure is negative.

Study description	This is a quantitative study using register data covering over 77.9 million live births from January 2015 to December 2021 from 15 countries (Americas: Brazil, Ecuador, Colombia, Mexico, the United States; and Europe: Austria, Denmark, England, Finland, the Netherlands, Scotland, Spain, Sweden, Wales; and the Australian state South Australia) we estimated the pandemic's effect on the socioeconomic and demographic composition of the cohort born between December 2020 and December 2021. To this end we used interrupted time series Poisson regression.
Research sample	Our study populations are the entire birth cohorts conceived before the pandemic and conceived during the pandemic in the 15 included countries/region. We use population-wide birth data. Thus our data does not represent a sample of the study population but the entire study population. Due to a few late registration of births, we may not capture all births in included countries. The limitations are discussed in the manuscript and in the supplementary material. The country specific data sources are described in the supplementary material.
Sampling strategy	Not applicable to population-wide data.
Data collection	Birth register data is collected by the responsible authorities in each country through birth certificates. Parental information is sometimes supplied by additional population registers that were linked to the birth registers. We describe the data for each country separately in the our country profiles.
Timing	Data collection was carried out by the respective national institutions; not by the authors of this study. We accessed the data used for our study during the year 2023. For countries with late registrations, register data for 2021 might still be updated by a few late registrations.

Data exclusions	Births with missing data on parental information (e.g., income) were not excluded but analysed separately. Results are presented throughout the main manuscript and the supplementary material
Non-participation	Not applicable to population-wide data. The study coves the full population of births of each country to the extent the birth register includes all births in a country.
Randomization	As this is an observational study, live births were not randomised to being conceived during the COVID-19 pandemic. We allocated live births to cohorts conceived during the pandemic based on their month or week of birth. We discuss potential misclassification of births in the discussion section of the manuscript.

Reporting for specific materials, systems and methods

We require information from authors about some types of materials, experimental systems and methods used in many studies. Here, indicate whether each material, system or method listed is relevant to your study. If you are not sure if a list item applies to your research, read the appropriate section before selecting a response.

Materials & experimental systems

n/a	Involved in the study
☒	☐ Antibodies
☒	☐ Eukaryotic cell lines
☒	☐ Palaeontology and archaeology
☒	☐ Animals and other organisms
☒	☐ Clinical data
☒	☐ Dual use research of concern
☒	☐ Plants

Methods

n/a	Involved in the study
☒	☐ ChIP-seq
☒	☐ Flow cytometry
☒	☐ MRI-based neuroimaging

Plants

Seed stocks	N/A
Novel plant genotypes	N/A
Authentication	N/A
